# Nanomaterial-enabled delivery of plant-derived bioactive metabolites for diabetic retinopathy: from evidence appraisal to preclinical translation

**DOI:** 10.3389/fphar.2026.1809139

**Published:** 2026-06-26

**Authors:** Dingmeng Zhao, Chuning Wang, Zhongshan Jiang, Yunxi Xu, Yihao Xia, Peiying Zhang, Hejiang Ye

**Affiliations:** 1 School of Ophthalmology, Chengdu University of Traditional Chinese Medicine, Chengdu, China; 2 Department of Ophthalmology, Hospital of Chengdu University of Traditional Chinese Medicine, Chengdu, China

**Keywords:** blood-retinal barrier, diabetic retinopathy, drug delivery, lipid carriers, nanomaterials, plant-derived bioactive metabolites, polymeric nanoparticles, preclinical efficacy

## Abstract

As a leading cause of preventable blindness globally, diabetic retinopathy (DR) requires innovative treatments beyond conventional anti-VEGF therapies, which face limitations in efficacy, administration burden, and multifactorial targeting. Plant-derived bioactive metabolites (e.g., quercetin, puerarin) have been reported to modulate multiple DR-relevant pathways in preclinical studies against DR pathogenesis, including modulation of angiogenesis, oxidative stress, and inflammation. However, their clinical translation is hindered by compound- and route-dependent pharmacokinetic challenges, including poor solubility, extensive metabolism, rapid ocular clearance, and restricted posterior-segment exposure. This review explores nanomaterial-enabled delivery systems to overcome these barriers, detailing platforms such as polymeric nanoparticles, lipid-based carriers, and exosomes engineered to enhance solubility, penetration, and sustained release. Key strategies including barrier-specific size optimization, surface engineering, and ligand functionalization are critically analyzed. Integrated preclinical frameworks utilizing *in vitro* models and *in vivo* rodent studies, along with pharmacokinetic and safety evaluations, are emphasized. Finally, regulatory considerations and clinical trial challenges are addressed. Nanomaterial-mediated delivery represents a paradigm shift and may provide a promising translational strategy for plant-derived bioactive metabolites in DR, but its clinical relevance will depend on rigorous ocular pharmacokinetic, efficacy, safety, manufacturing, and regulatory validation.

## 1 Introduction

Diabetic retinopathy (DR) constitutes a global health emergency, with its prevalence affecting approximately 103.12 million adults in 2020 and projected to reach 160.50 million by 2045, as established by comprehensive epidemiological meta-analyses (Teo et al., 2021). The pathogenesis involves hyperglycemia-induced microvascular damage through interconnected pathways: polyol flux, advanced glycation end-product (AGE) accumulation, protein kinase C (PKC) activation, hexosamine pathway dysregulation, and oxidative stress (Wang and Lo, 2018; Yang et al., 2024). These mechanisms collectively drive vascular leakage, inflammation, neurodegeneration, and aberrant angiogenesis—culminating in retinal ischemia and edema (Caridi et al., 2021). Current anti-VEGF therapies, while revolutionary, face significant limitations including systemic side effects (hypertension, thromboembolic events), invasive intravitreal administration requiring frequent injections, short intravitreal half-life, and high treatment burden (Klotzsche-von Ameln and Sprott, 2022; Moiseev et al., 2019). Moreover, anti-VEGF monotherapy fails to address the multifactorial pathophysiology of DR, leaving a critical therapeutic gap (Raman et al., 2022).

**FIGURE 1:**
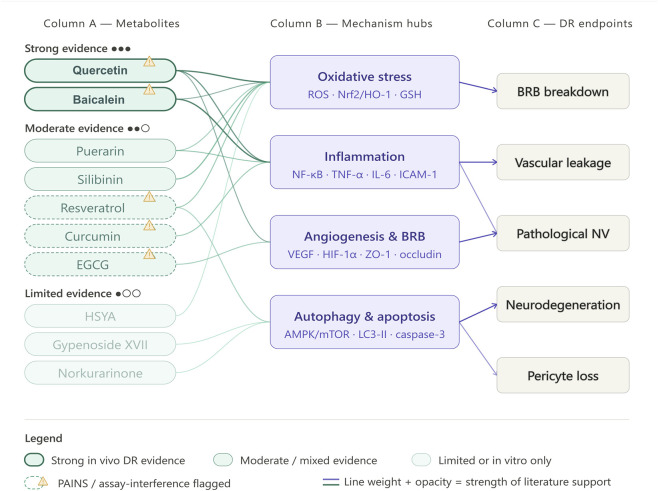
Proposed multi-target molecular mechanisms of representative plant-derived bioactive metabolites in diabetic retinopathy. This schematic summarizes the major diabetic retinopathy (DR)-relevant molecular pathways potentially modulated by representative plant-derived bioactive metabolites. These metabolites may affect oxidative stress, inflammation, angiogenesis, blood-retinal barrier (BRB) dysfunction, autophagy, and apoptosis through pathways involving ROS/Nrf2/HO-1/GSH, NF-κB/TNF-α/IL-6/ICAM-1, VEGF/HIF-1α/ZO-1/occludin, and AMPK/mTOR/LC3-II/caspase-3 signaling. The figure also indicates the relative strength of available evidence, distinguishing metabolites supported by stronger *in vivo* DR evidence from those with moderate, mixed, or mainly *in vitro* evidence. Potential PAINS or assay-interference concerns are noted for selected polyphenolic or redox-active compounds. PAINS/assay-interference triangles indicate compounds whose *in vitro* readouts warrant orthogonal validation; this annotation does not negate *in vivo* evidence and is independent of the evidence-strength category.

Plant-derived bioactive metabolites, including quercetin, puerarin, baicalein, and silibinin/silybin, have been investigated for their potential multi-target activity against DR-relevant or retinal pathogenic processes. In streptozotocin-induced diabetic rats, quercetin has been reported to exert retinoprotective effects associated with antioxidant defense and heme oxygenase-1 induction (Chai et al., 2021). Puerarin has been shown to attenuate advanced glycation end product-induced retinal pericyte apoptosis by suppressing NADPH oxidase-related oxidative stress and NF-κB activation (Kim et al., 2012). Baicalein, the aglycone of baicalin, has been reported to reduce retinal inflammatory activation, Müller-cell GFAP/VEGF expression, vascular abnormalities, and ganglion-cell apoptosis in a rodent model of diabetic retinopathy (Lv et al., 2024a; Yang et al., 2009). Silybin, the major active flavonolignan of silymarin/silibinin preparations, has been reported to reduce obliterated retinal capillaries, leukostasis, and ICAM-1 expression in experimental diabetic retinopathy rats (Gaudana et al., 2010; Zhang et al., 2014). Cochrane systematic reviews underscore the “low-certainty evidence” for Chinese botanical medicine interventions, citing inadequate placebo controls, heterogeneity in Chinese botanical medicine formulations, and insufficient reporting of composition/dosage in 60.8% of studies (Zhang et al., 2019; Hu et al., 2011). Critically, 96.6% of systematic reviews on Chinese botanical medicines exhibit “critically low” methodological quality, further obscuring efficacy validation (Cheung et al., 2022). The proposed multi-target molecular mechanisms of these representative metabolites against DR pathogenesis are summarized in Figure 1, which integrates oxidative-stress, inflammatory, apoptotic, and angiogenic nodes that are addressed in greater detail below.

Nanomaterial-based delivery systems present a transformative strategy to overcome these barriers. Polymeric nanoparticles, including poly (lactic-co-glycolic acid) (PLGA) and chitosan nanoparticles, and lipid-based carriers, including liposomes and nanostructured lipid carriers (NLCs), enhance ocular bioavailability through multiple mechanisms: (1) improved corneal adhesion via mucoadhesive polymers such as chitosan; (2) sustained release kinetics minimizing dosing frequency; (3) protection of labile plant-derived metabolites from enzymatic degradation; and (4) enhanced penetration across epithelial tight junctions via nano-scale dimensions (<100 nm) (Mahaling et al., 2023; Meza-Rios et al., 2020; Swetledge et al., 2021). For posterior segment delivery, exosomes (30–150 nm) demonstrate superior biocompatibility and barrier penetration, leveraging endogenous transport pathways to bypass static (corneal epithelium) and dynamic (choroidal blood flow) ocular barriers (Li et al., 2023; Gupta et al., 2021). Recent preclinical studies suggest potential complementary effects: co-encapsulation of lipophilic and hydrophilic metabolites such as silibinin and puerarin in nanostructured lipid carriers (NLCs) has been reported to enhance *in vitro* anti-inflammatory activity through improved biodistribution (Iordache et al., 2021). In DR-relevant rodent models, baicalin-loaded liposomes have been reported to lower retinal VEGF expression and reduce vascular leakage relative to free-drug controls, and quercetin nanoemulsions to prolong retinal residence, although the magnitude of these effects varies across individual studies and quantitative ocular pharmacokinetic confirmation is generally lacking. These representative examples are discussed in greater detail, with primary-source citations and evidence appraisal below.

In conclusion, while plant-derived bioactive metabolites show preclinically reported multi-target activities against DR pathogenesis, their clinical potential remains unrealized due to pharmacokinetic constraints and methodological limitations in existing studies. Nanomaterial-enabled delivery systems—spanning polymeric nanoparticles, liposomes, and exosomes—address these challenges by enhancing corneal/retinal penetration, enabling sustained release, and amplifying synergistic actions. Future research must prioritize rigorous preclinical validation using standardized purified-metabolite interventions, clinically relevant endpoints (e.g., vascular leakage quantification via fluorescein angiography), and adherence to Cochrane methodological standards to translate nanocarrier advantages into therapeutic breakthroughs (Sarkar et al., 2025).

## 2 Review methodology and evidence appraisal

### 2.1 Literature search strategy

A structured literature search was conducted to reduce selection bias and to avoid a purely descriptive or arbitrary compilation of studies. PubMed, Web of Science, Scopus, ScienceDirect, and Google Scholar were searched from database inception to 31 December 2025. The search combined disease-related, compound-related, and delivery-related terms using Boolean operators. A representative PubMed search string was: (“diabetic retinopathy” OR “diabetic macular edema” OR “retinal vascular leakage” OR “blood-retinal barrier”) AND (“plant-derived metabolite” OR “botanical metabolite” OR quercetin OR puerarin OR baicalin OR baicalein OR silibinin OR curcumin OR resveratrol OR berberine OR tetramethylpyrazine OR notoginsenoside) AND (“nanoparticle” OR “nanocarrier” OR liposome OR “nanostructured lipid carrier” OR “solid lipid nanoparticle” OR exosome OR hydrogel OR “ocular delivery” OR “retinal delivery” OR pharmacokinetics OR biodistribution OR toxicity). Reference lists of relevant reviews and primary studies were manually screened to identify additional eligible studies.

### 2.2 Eligibility criteria and study selection

Studies were included if they met at least one of the following criteria: (i) investigated purified plant-derived bioactive metabolites or chemically characterized botanical preparations relevant to diabetic retinopathy (DR); (ii) evaluated nanomaterial-enabled ocular, systemic, periocular, or intravitreal delivery systems; (iii) reported DR-relevant mechanisms, including oxidative stress, inflammation, VEGF signaling, blood-retinal barrier dysfunction, apoptosis, autophagy, or retinal neurodegeneration; or (iv) provided pharmacokinetic, biodistribution, ocular exposure, or safety data relevant to retinal translation.

**FIGURE 2:**
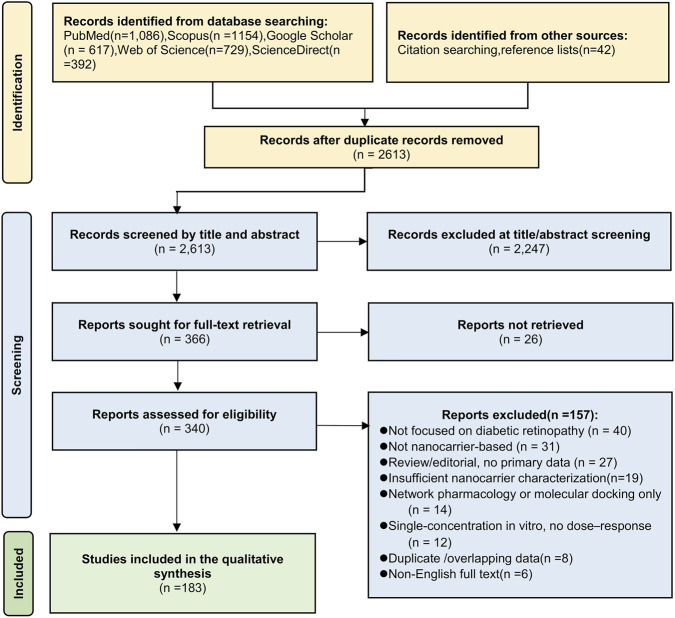
PRISMA 2020 flow diagram of study identification, screening, and selection. The flow diagram summarizes the literature identification and selection process used in this review. Records were retrieved from PubMed, Web of Science, Scopus, ScienceDirect, Google Scholar, and additional citation searching. After duplicate removal, studies were screened by title and abstract, followed by full-text eligibility assessment. Exclusion criteria included lack of DR relevance, absence of nanocarrier-based delivery, review or editorial-only publications, insufficient nanocarrier characterization, network pharmacology or molecular docking alone, single-concentration *in vitro* studies without dose-response validation, duplicated data, and unavailable full texts. The final included studies were used for qualitative synthesis and evidence appraisal.

Studies were excluded or interpreted as low-priority evidence when they relied solely on network pharmacology, molecular docking, single-concentration *in vitro* assays, poorly defined extracts, undefined formulation composition, missing particle characterization, absent dose-response analysis, or no DR-relevant model system. Clinical studies, systematic reviews, and preclinical studies were discussed separately to avoid conflating clinical efficacy with preclinical activity. The selection process followed a narrative-review adaptation of PRISMA principles: records were first screened by title and abstract for relevance to DR, plant-derived metabolites, and nanodelivery; full texts were then evaluated for formulation definition, experimental design, biological endpoints, and translational relevance. Studies were not pooled quantitatively because of substantial heterogeneity in metabolites, nanocarriers, disease models, routes of administration, and outcome measures. The complete study identification, screening, and selection process is summarized in Figure 2, presented according to PRISMA 2020 reporting principles adapted for narrative review.

### 2.3 Material classification and taxonomic verification

To clarify how pharmacological claims were attributed in this review, study materials were classified into three categories: (i) purified plant-derived bioactive metabolites (e.g., quercetin, puerarin, baicalin/baicalein, silibinin, curcumin, resveratrol, berberine, tetramethylpyrazine, notoginsenosides), for which chemical identity, purity, source, analytical confirmation, and stability were considered essential reporting items; (ii) metabolite-loaded nanoformulations, for which both the active metabolite and the carrier system had to be defined, including particle size, polydispersity index (PDI), zeta potential, morphology, encapsulation efficiency, drug loading, release kinetics, stability, and sterility/endotoxin status where relevant; and (iii) botanical preparations, extracts, or multi-component formulations, for which complete botanical composition, plant parts, processing, chemical profile, and marker compounds had to be specified before pharmacological claims were considered reliable.

For studies in category (iii), plant names were verified against the Medicinal Plant Names Services (MPNS, Royal Botanic Gardens, Kew) and Plants of the World Online (POWO). Accepted scientific names, families, and taxonomic authorities were recorded where available. When original studies used only vernacular names, incomplete Latin binomials, unresolved synonyms, or undefined “herbal” formulations, the evidence was downgraded because the pharmacological material could not be reproduced or critically evaluated. Multi-component preparations were not used to support compound-specific mechanisms unless the full botanical composition, plant parts, extraction or processing method, chemical profile, and marker compounds were reported. A taxonomic verification record for representative botanical sources discussed in this review is provided as Supplementary Table S1.

### 2.4 Critical appraisal framework and ConPhyMP compliance

The scientific quality of original studies was critically appraised using predefined translational criteria: (i) chemical definition of the active metabolite or botanical preparation; (ii) nanocarrier characterization, including size, PDI, zeta potential, morphology, drug loading, encapsulation efficiency, and release kinetics; (iii) biological model relevance to DR; (iv) use of appropriate controls, including free compound, blank carrier, disease-model control, and positive comparator where applicable; (v) presence of dose-response and time-course data; (vi) evidence of target engagement or pathway validation; (vii) ocular pharmacokinetic or biodistribution evidence; (viii) safety assessment, including cytotoxicity, ocular irritation, inflammation, and repeated-dose tolerance; and (ix) reporting of randomization, masking, sample-size justification, and statistical methods in animal studies.

Because medicinal plant extracts and botanical preparations have unique reproducibility challenges that differ from purified single compounds, studies involving extracts or multi-component preparations were additionally checked against the ConPhyMP/Best Practice reporting framework, which emphasizes transparent reporting of plant material, extraction/processing, chemical profiling, and batch-to-batch consistency (Heinrich et al., 2022). The ConPhyMP guideline aims to improve reproducibility and accurate interpretation of pharmacological, toxicological, and clinical/intervention studies using medicinal plant extracts; plant material and initial processing and analytical methods for extract type A checklists completed for this review are provided as Supplementary Tables S2, S3 (Heinrich et al., 2022). Because the present review focuses primarily on purified plant-derived bioactive metabolites rather than newly prepared crude extracts, extract-specific items were marked as not applicable where appropriate.

Evidence was graded as high priority when *in vivo* DR-relevant models were combined with defined formulations, retinal exposure data, prespecified vascular or functional endpoints, and safety readouts; as moderate priority when retinal cell or barrier models were used with dose-response data and defined formulation properties but without *in vivo* ocular exposure or functional validation; and as low priority when conclusions rested on single-concentration *in vitro* assays, network-pharmacology predictions, molecular docking alone, undefined extracts, or nanoformulations without adequate physicochemical characterization. Throughout this review, claims were therefore phrased to distinguish “preclinically reported activity” from clinically demonstrated efficacy.

Pharmacological claims were further assessed according to whether the original studies reported sufficient experimental detail to support the stated activity. For each key study, the tested dose or concentration range, the lowest effective dose or concentration where available, exposure or treatment duration, route of administration, model system, disease relevance of the model, primary pharmacological endpoints, comparator groups, and the presence of dose-response or time-course relationships were extracted. Claims based only on single-dose or single-concentration experiments, non-retinal cell models, network-pharmacology predictions, or pharmacological endpoints without ocular exposure data were downgraded and interpreted as hypothesis-generating rather than confirmatory. The corresponding study-category appraisal matrix and a study-by-study evidence map are provided as Supplementary Tables S4, S5.

A consolidated overview of the appraisal domains, the evidence considered high quality, the most common limitations identified, and the resulting interpretation in this review is presented in Table 1.

**TABLE 1:** Critical appraisal framework for included preclinical and translational studies.

Appraisal domain	High-quality evidence	Common limitations identified	Interpretation in this review
Compound definition	Purified metabolite with identity, purity, source, and analytical confirmation reported	Undefined extract, unclear purity, no batch information	Not used to support strong mechanistic or translational claims
Botanical/extract reporting	Plant species, family, authority, plant part, extraction method, chemical profile, and batch consistency reported	Missing taxonomy, unclear extraction method, no chemical profile	Evidence downgraded according to ConPhyMP principles
Nanocarrier characterization	Size, PDI, zeta potential, morphology, encapsulation efficiency, drug loading, and release kinetics reported	Only particle size reported; no stability or release data	Delivery advantage considered preliminary
Model relevance	STZ/OIR/*in vivo* retinal models or validated retinal-barrier models	Generic cell lines or non-DR models	DR relevance considered limited
Controls	Free compound, blank carrier, disease control, and positive comparator included	Missing blank carrier or free-compound control	Carrier effect cannot be separated from metabolite effect
Dose-response	Multiple concentrations, doses, or time points	Single concentration only	Treated as hypothesis-generating
PK/biodistribution	Retina/choroid/vitreous/plasma concentrations quantified over time	No ocular exposure data	Retinal targeting claims not considered established
Safety	Cytotoxicity, ocular tolerance, inflammation, and repeated-dose assessment	Only acute viability assay	Translational readiness considered low
Bias control	Randomization, masking, statistics, and sample-size rationale described	No randomization, blinding, or sample-size justification	Evidence quality downgraded
Assay interference/PAINS	Use of orthogonal assays, dose–response curves, target-engagement readouts, counter-screens for redox cycling, aggregation, fluorescence interference, and metal chelation; *in vivo* confirmation	Single-readout assays (ROS fluorescence, ELISA, viability) without orthogonal validation; high test concentrations of polyphenols (quercetin, curcumin, resveratrol, baicalin/baicalein) where PAINS-like behaviour is plausible	Effects treated as hypothesis-generating only; not interpreted as definitive pharmacological activity unless ocular exposure, target engagement, and orthogonal validation are reported

DR, diabetic retinopathy; OIR, oxygen-induced retinopathy; PDI, polydispersity index; PK, pharmacokinetics; STZ, streptozotocin.

### 2.5 PAINS and assay-interference considerations

A specific caution was applied to polyphenolic and redox-active metabolites, including quercetin, curcumin, resveratrol, baicalin/baicalein, and related flavonoids, because some members of these chemical classes may behave as pan-assay interference compounds (PAINS) or promiscuous assay interferers (Magalhães et al., 2021). PAINS may generate false-positive or non-specific readouts through redox cycling, colloidal aggregation, fluorescence interference, metal chelation, membrane perturbation, covalent reactivity, non-covalent promiscuous binding, or non-specific protein interactions (Magalhães et al., 2021; Bolz et al., 2021). Bolz et al. (2021) emphasized that PAINS are promiscuous compound classes that can produce false-positive hits in high-throughput screening, and their mechanisms may involve non-covalent as well as covalent interactions.

Accordingly, *in vitro* antioxidant, anti-inflammatory, anti-apoptotic, or anti-angiogenic effects were not interpreted as definitive pharmacological or clinically relevant activity unless supported by orthogonal assays, concentration-response relationships, target-engagement evidence, retinal or ocular exposure data, and *in vivo* validation. Studies relying only on single readouts such as ROS fluorescence, cytokine ELISA, VEGF suppression, molecular docking, or cell viability assays were treated as hypothesis-generating rather than confirmatory. This distinction is particularly important for DR research because improved cellular readouts do not necessarily demonstrate retinal delivery, blood-retinal barrier penetration, sustained ocular exposure, disease-modifying efficacy, or clinical translatability.

## 3 Key translational bottlenecks in plant-derived bioactive metabolite therapy for DR

### 3.1 Pharmacokinetic and ocular-exposure limitations

The therapeutic application of plant-derived bioactive metabolites for diabetic retinopathy (DR) is constrained by compound-specific pharmacokinetic and ocular-exposure limitations. Many representative metabolites, including flavonoids, tannins, terpenoids, and saponins, have poor aqueous solubility, limited dissolution, extensive metabolism, and insufficient posterior-segment exposure. For example, quercetin shows low and species-/formulation-dependent oral bioavailability; original pharmacokinetic studies have reported low systemic exposure after oral dosing, including an absolute bioavailability of approximately 4% in dogs (Reinboth et al., 2010), with most circulating flavonols present as conjugated metabolites (Wang et al., 2024; Jalili et al., 2023). Pharmacokinetic studies in humans and rats further confirm that orally administered quercetin is rapidly conjugated; quercetin aglycone is essentially absent from plasma, while glucuronide, sulfate, and methylated conjugates dominate, and absolute oral bioavailability of conventional aqueous suspensions is below 5% in rats (Zhu X. et al., 2024; Li H. et al., 2021). Improved dissolution from a nanocarrier therefore does not necessarily increase exposure to the pharmacologically relevant aglycone in the retina. Nanoencapsulation can improve dispersibility and apparent solubility; for instance, taxifolin dehydrate nanoparticles achieved 91% dispersibility within 30 min, mainly through particle-size reduction and loss of crystallinity (Slimane, 2017). However, improved dispersibility alone should be interpreted as formulation-enabling evidence rather than proof of retinal therapeutic efficacy.

Pharmacokinetic studies of Anemarrhena asphodeloides-derived saponins further illustrate why generalized statements about “poor bioavailability” or “hepatic extraction” should be avoided. In a Baihe–Zhimu decoction study, timosaponin AIII showed a high liver extraction ratio of approximately 73.20%, whereas timosaponin BII and timosaponin BIII showed much lower extraction ratios of 10.88% and 11.57%, respectively (Zhong et al., 2019). Timosaponin BII also shows very low absolute oral bioavailability in rats, reported at approximately 1.1%, but this limited exposure appears to involve poor intestinal absorption, gut microbiota-mediated biotransformation, and transporter-dependent hepatobiliary handling rather than simple hepatic extraction alone (Sheng et al., 2015; Dong et al., 2021). Therefore, for timosaponins and related saponins, reduced systemic exposure should be interpreted as a compound-specific pharmacokinetic problem involving absorption, metabolism, transporter activity, and biliary disposition, rather than as a uniform first-pass metabolism effect. This distinction is important for nanocarrier design: permeability-enhancing or transporter-aware delivery systems may be more rational for poorly absorbed saponins, whereas simple solubilization strategies alone may be insufficient.

Silibinin (the principal flavonolignan of silymarin) is a further example of solubility- and conjugation-limited exposure. Conventional silymarin shows poor intestinal absorption and rapid first-pass conjugation, with low plasma silybin concentrations after oral dosing; phospholipid-complex formulations (silybin–phosphatidylcholine, IdB 1016/Siliphos) increase systemic silybin exposure approximately 4.6- to 9.6-fold relative to standard silymarin tablets in healthy volunteers (Méndez-Sánchez et al., 2019; Barzaghi et al., 1990), confirming that the limiting step is dissolution and absorption rather than systemic clearance. However, these hepatic/systemic exposure improvements have not been translated into validated retinal or vitreous concentrations in DR-relevant models, so silibinin-loaded ocular nanoformulations should not be assumed to deliver therapeutically meaningful retinal levels without quantitative ocular pharmacokinetic data (Di Costanzo and Angelico, 2019).

Botanical metabolites may also influence drug-metabolizing enzymes and efflux transporters, creating potential safety and drug–drug interaction concerns. For example, hyperforin in St. John’s wort can induce intestinal P-glycoprotein and hepatic CYP3A4, illustrating that botanical exposure may alter both systemic pharmacokinetics and co-administered drug disposition (Ioannides, 2002; Dürr et al., 2000). Together, these examples show that pharmacokinetic limitations are metabolite-, formulation-, route-, and model-dependent, and should not be presented as uniform properties of all plant-derived bioactive metabolites.

Viewed comparatively, the limiting step is not the same across metabolites. Puerarin and berberine are absorption-limited examples: puerarin shows about 7% absolute oral bioavailability in rats, whereas berberine is reported at 0.68% (Anukunwithaya et al., 2018; Chen et al., 2011); therefore, increasing systemic exposure alone still may not translate into retinal efficacy unless retina-to-plasma exposure is quantified. Resveratrol follows a different pattern: human oral absorption is relatively high (about 75%), but systemic bioavailability remains below 1% because of rapid intestinal and hepatic metabolism (Walle, 2011; Walle et al., 2004). Curcumin and quercetin are primarily constrained by hydrophobicity, poor dissolution, extensive conjugation, and concentration-dependent assay-interference risks. More specifically, curcumin combines very low aqueous solubility (∼0.6 μg/mL), poor intestinal absorption, extensive intestinal and hepatic phase-II conjugation (mainly glucuronidation and sulfation), and rapid systemic elimination, so that even high oral doses yield only nanomolar parent-drug plasma concentrations in humans; under these constraints, *in vitro* antioxidant or anti-inflammatory readouts must be interpreted alongside its known PAINS-like behaviour, and retinal exposure cannot be inferred from improved dissolution alone (Tabanelli et al., 2021; Dei Cas and Ghidoni, 2019). On this basis, nanocarrier selection should be metabolite-specific rather than platform-driven: solubilizing lipid systems are most defensible for hydrophobic polyphenols, mucoadhesive or permeability-enhancing systems for poorly absorbed glycosides and alkaloids, and sustained periocular/intravitreal systems for metabolites that require posterior-segment residence.

Ocular exposure remains a separate and particularly important barrier. Static barriers, including the cornea and blood-retinal barrier (BRB), and dynamic barriers, including tear dilution, conjunctival clearance, and nasolacrimal drainage, restrict retinal delivery (Bora et al., 2023). Although nanocarriers may improve corneal retention, protect labile metabolites, and enable sustained release, claims of improved posterior-segment delivery require quantitative ocular pharmacokinetic data, including retina/choroid, vitreous, aqueous humor, cornea, and plasma concentrations over time. Therefore, the pharmacokinetic limitations of plant-derived bioactive metabolites should be discussed as formulation-, route-, and model-dependent rather than as uniform properties across all metabolites. The specific pharmacokinetic challenges of representative plant-derived bioactive metabolites are systematically compared in Table 2.

**TABLE 2:** Comparative pharmacokinetic and ocular-exposure limitations of representative plant-derived bioactive metabolites relevant to DR translation.

Metabolite	Chemical/Pharmacokinetic feature	Main systemic PK limitation	Ocular/Retinal translation gap	Nanocarrier implication
Puerarin	Isoflavone C-glycoside; relatively hydrophilic but absorption-limited	Absolute oral bioavailability in rats is approximately 7%, indicating limited systemic exposure after oral dosing	Increased plasma exposure alone does not prove retinal delivery; retina-to-plasma ratios after clinically relevant topical, periocular, or intravitreal routes remain insufficiently characterized	Mucoadhesive, permeability-enhancing, or sustained-release systems may be more defensible than simple solubilization-only platforms
Berberine	Isoquinoline alkaloid; absorption-limited and affected by intestinal transport/metabolism	Very low absolute oral bioavailability has been reported in rats, around 0.68%	Retinal exposure after systemic administration is uncertain; systemic dose escalation may increase off-target exposure without ensuring posterior-segment efficacy	Permeability-enhancing, transporter-modulating, or localized ocular delivery systems should be prioritized; systemic exposure should not be used as a surrogate for retinal efficacy
Resveratrol	Lipophilic polyphenol with rapid metabolic conversion	Human oral absorption is relatively high, about 75%, but systemic bioavailability is below 1% because of extensive intestinal and hepatic metabolism	High absorption does not equal high active retinal exposure; parent-compound versus metabolite exposure in ocular tissues should be distinguished	Protective lipid/polymeric carriers, sustained-release systems, or local delivery may help reduce rapid metabolic loss, but ocular PK confirmation is essential
Curcumin	Hydrophobic polyphenol; chemically unstable and prone to assay-interference concerns	Poor absorption, limited biodistribution, rapid metabolism, and low bioavailability restrict systemic translation	*In vitro* antioxidant or anti-inflammatory readouts may be confounded by instability, reactivity, or PAINS-like behavior; retinal exposure is usually not established	Lipid nanoparticles, polymeric nanoparticles, micelles, or nanoemulsions may improve apparent exposure, but claims require orthogonal assays, dose-response validation, and quantitative ocular PK
Quercetin	Hydrophobic flavonol; undergoes extensive phase II metabolism	Clinical use is constrained by poor water solubility, substantial first-pass metabolism, and variable bioavailability	Retinal delivery cannot be inferred from improved dissolution or plasma exposure; conjugated metabolites may differ from parent quercetin in activity	Solubilizing lipid systems, cyclodextrin-based systems, nanoemulsions, or NLCs may be rational, but retinal/choroidal exposure and active species should be measured
Silibinin/silymarin	Flavonolignan with poor aqueous solubility	Poor water solubility and low oral bioavailability limit therapeutic application; formulation approaches such as phospholipid complexes, micelles, and nanosystems have been explored	DR-specific retinal exposure data are limited; hepatic or systemic bioavailability improvement should not be directly extrapolated to ocular efficacy	Lipid-based carriers, phospholipid complexes, micelles, NLCs, or liposomes are mechanistically appropriate for solubilization and exposure enhancement
Notoginsenosides/ginsenoside-type saponins	Large glycosylated saponins with poor membrane permeability	Many ginsenosides show poor absorption and low oral bioavailability; disposition is strongly compound-specific	Limited evidence supports direct retinal targeting; systemic exposure may not reach pharmacologically relevant ocular concentrations	Permeability-enhancing carriers, local ocular depots, or targeted delivery systems should be considered, but retinal biodistribution is required

In summary, poor solubility, metabolic instability, short systemic half-lives, and ocular barrier restrictions collectively limit the translational potential of plant-derived bioactive metabolites for DR. These limitations should be interpreted as molecule-, formulation-, route-, and model-dependent rather than as uniform properties of all metabolites. Overcoming them requires integrated nanotechnological approaches that optimize drug delivery, enhance bioavailability, and ensure sustained retinal drug exposure.

### 3.2 Retinal targeting, systemic exposure, and safety risks

A major translational gap is the limited evidence for selective retinal targeting. Plant-derived bioactive metabolites such as puerarin, tetramethylpyrazine, and notoginsenosides have been investigated for ocular or systemic pharmacokinetics, but direct comparisons of retinal, vitreous, choroidal, and plasma exposure remain scarce. Oral puerarin shows poor systemic bioavailability in rats, with an absolute oral bioavailability of approximately 7%, indicating that systemic exposure alone is unlikely to ensure therapeutically meaningful posterior-segment delivery (Anukunwithaya et al., 2018). However, route and formulation design can substantially alter ocular exposure. In a rabbit microdialysis study, puerarin *in situ* gel eye drops increased aqueous-humor exposure, with Cmax and AUC0– ∞ values 3.77- and 4.40-fold higher than those of puerarin solution, respectively (Jin et al., 2023). More directly relevant to posterior-segment delivery, puerarin-loaded poly (lactic acid) microspheres administered by intravitreal injection showed sustained-release behavior and produced higher Cmax and AUC0– ∞ values in the retina and choroid than puerarin solution or intravenous injection. Nevertheless, these studies mainly support improved ocular or posterior-segment exposure rather than selective retinal targeting, and comprehensive retina/choroid-to-plasma exposure ratios after clinically relevant topical, periocular, or intravitreal delivery remain insufficiently characterized (Long et al., 2024).

For Panax notoginseng saponins, the limiting step is membrane permeability and active biliary efflux rather than metabolism: in rats, ginsenoside Rb1 (a PPD-type saponin) shows absolute oral bioavailability of approximately 0.6%–4.3% and ginsenoside Rg1 (PPT-type) approximately 18% (Han et al., 2006; Xu et al., 2003), with substantial colonic deglycosylation but low circulating concentrations of either parent saponins or their deglycosylated metabolites (Liu et al., 2009). Consequently, increasing systemic exposure of notoginsenosides through permeability-enhancing or local-depot nanocarriers is mechanistically more rational than purely solubilising platforms, but retinal biodistribution after any of these routes remains undefined.

These limitations are important because systemic dose escalation may increase off-target exposure without guaranteeing retinal efficacy. High circulating concentrations of some plant-derived metabolites or botanical preparations may affect hepatic metabolism, renal clearance, gastrointestinal tolerance, or drug–drug interaction risk. Therefore, safety claims should not be extrapolated from acute toxicity or non-ocular models alone. For multi-component botanical formulations, such as PHF-dia, reported absence of acute toxicity at high doses may be useful as preliminary safety information, but it does not establish long-term ocular safety, retinal exposure, or disease-modifying efficacy in DR.

Consequently, this review treats retinal targeting as unproven unless supported by quantitative ocular biodistribution, appropriate free-metabolite and blank-carrier controls, and DR-relevant functional endpoints. Improved systemic bioavailability, increased cellular uptake, or detectable retinal accumulation should be considered supportive delivery evidence, not definitive proof of therapeutic superiority.

### 3.3 Mechanistic uncertainty and model-dependent evidence gaps

Although plant-derived bioactive metabolites have been reported to modulate oxidative stress, inflammation, angiogenesis, apoptosis, and autophagy in DR-related models, the mechanistic evidence remains uneven across molecules, cell types, disease stages, and formulations. Autophagy illustrates this complexity. Hyperglycemia can alter AMPK/mTOR signaling and autophagic flux, but whether metabolites such as resveratrol or hydroxy saffron yellow A restore protective autophagy or suppress maladaptive autophagy depends on exposure level, disease stage, and retinal cell type (Jin et al., 2023; Sun et al., 2023; Semeraro et al., 2015; Li M. et al., 2021; Zheng et al., 2023). Similarly, reported suppression of NF-κB, IL-17A/JAK1, VEGF, or inflammatory cytokines may reflect genuine pathway modulation, but many studies rely on limited readouts such as ROS fluorescence, cytokine ELISA, VEGF expression, or cell viability assays (Wei et al., 2022; Qin et al., 2023; Razavi et al., 2022; Kocak et al., 2022). These endpoints are useful for screening but are insufficient to establish target engagement or disease-modifying efficacy.

Mechanistic interpretation should therefore move from isolated pathways toward an integrated stress–inflammation–autophagy–apoptosis axis. Quercetin is a representative example of a metabolite for which mechanistic claims are pathway-resolved in DR-relevant models: in streptozotocin-induced diabetic rats, oral quercetin reduced retinal NF-κB and caspase-3 expression, lowered TNF-α and IL-1β, and attenuated GFAP and AQP4 upregulation in Müller cells (Kumar et al., 2014); in mouse photoreceptor 661W cells, quercetin suppressed VEGF-induced inflammation by inactivating NF-κB through inhibition of upstream MAPK and Akt phosphorylation (Lee et al., 2017); and in IL-1β-stimulated ARPE-19 cells, quercetin reduced ICAM-1, IL-6, IL-8, and MCP-1 expression by inhibiting MAPK phosphorylation and NF-κB p65 nuclear translocation (Cheng et al., 2019). These three orthogonal lines of evidence justify the MAPK/NF-κB and mitochondrial/apoptosis nodes assigned to quercetin in Table 3. Hyperglycemia-driven ROS can activate NF-κB signaling and inflammatory cytokine release, disrupt tight-junction proteins such as ZO-1 and occludin, promote VEGF/ICAM-1-mediated vascular leakage, and alter AMPK/mTOR-dependent autophagic flux (Wang et al., 2022; Qu et al., 2003). Plant-derived bioactive metabolites may affect one or more of these nodes, but the strength of evidence differs substantially by metabolite, formulation, model, and endpoint. In particular, polyphenolic and redox-active metabolites require orthogonal assays and dose-response validation because some readouts may be vulnerable to assay interference.

**TABLE 3:** Representative molecular mechanisms by which plant-derived bioactive metabolites may affect DR-related oxidative stress, inflammation, autophagy, and apoptosis pathways.

Metabolite	Proposed molecular nodes	DR-relevant pathway	Reported biological effect	Main evidence limitation
Quercetin	MAPK/NF-κB, mitochondrial membrane potential, apoptosis-related markers	Oxidative stress, inflammation, apoptosis, angiogenesis	Reported anti-inflammatory, antioxidant, anti-apoptotic, and anti-angiogenic effects in preclinical DR-related models	Evidence varies by model; retinal exposure, dose-response, and target engagement are often incomplete
Resveratrol	AMPK/mTOR, Nrf2/HO-1, Beclin-1, Bcl-2/Bax	Autophagy, oxidative stress, apoptosis	Proposed to restore autophagic balance and reduce oxidative/apoptotic injury	Autophagic flux is not always rigorously quantified; ocular PK and cell-type-specific effects remain limited
HSYA	ROS-related signaling, AMPK/mTOR-related autophagy, inflammatory mediators	Oxidative stress, autophagy, inflammation	Proposed neurovascular protection under diabetic stress	Molecular evidence remains fragmented; retinal cell-specific validation is insufficient
Baicalein	Inflammatory cytokines, Müller-cell GFAP/VEGF, HIF-1α/VEGF/MMP-9, HO-1/Bcl-2	Retinal inflammation, angiogenesis-related signaling, vascular abnormality, oxidative/apoptotic injury	Reported to reduce retinal inflammatory activation, Müller-cell gliosis, VEGF upregulation, vascular abnormalities, and ganglion-cell loss in STZ-diabetic rats; also showed anti-ischemic retinal protection through HIF-1α/VEGF/MMP-9 downregulation and HO-1/Bcl-2 upregulation	Stronger *in vivo* vascular endpoints are reported, but dose-response and quantitative ocular PK remain limited
Curcumin	NF-κB, cytokine signaling, oxidative-stress markers	Inflammation, oxidative stress, apoptosis	Reported suppression of inflammatory cytokines and oxidative-stress readouts	PAINS/assay-interference concern; requires orthogonal assays and ocular exposure validation
Gypenoside XVII	AMPK/mTOR, autophagosome formation, apoptosis-related markers	Autophagy, Müller-cell stress, apoptosis	Reported modulation of autophagy and protection against high-glucose-induced cellular injury	Mainly *in vitro*; retinal biodistribution and *in vivo* DR validation are limited
Norkurarinone	PI3K/AKT, autophagy-related markers	RPE survival, autophagy, apoptosis	Reported inhibition of excessive autophagy and preservation of RPE viability	Model and formulation details are limited; ocular PK not established
EGCG/epigallocatechin	VEGF-related signaling, oxidative-stress pathways	Angiogenesis, oxidative stress, neuroprotection	Reported VEGF reduction and retinal-cell protective effects in nanoparticle systems	Nanoparticle safety, dose-response, and long-term retinal retention require further validation

Future mechanistic studies should prioritize DR-relevant retinal endothelial, RPE, Müller glial, microglial, and inner BRB models; validated *in vivo* models with vascular or functional endpoints; and spatially resolved methods such as *in vivo* imaging, single-cell or spatial transcriptomics, and pathway-specific target-engagement assays. Without these approaches, mechanistic claims should remain restricted to preclinical and hypothesis-generating interpretations. To avoid treating these pathways as generic or interchangeable mechanisms, representative metabolites and their proposed molecular nodes are summarized in Table 3.

## 4 Nanomaterial-based strategies to overcome delivery barriers

### 4.1 Nanocarrier platform selection based on metabolite properties and delivery route

**FIGURE 3:**
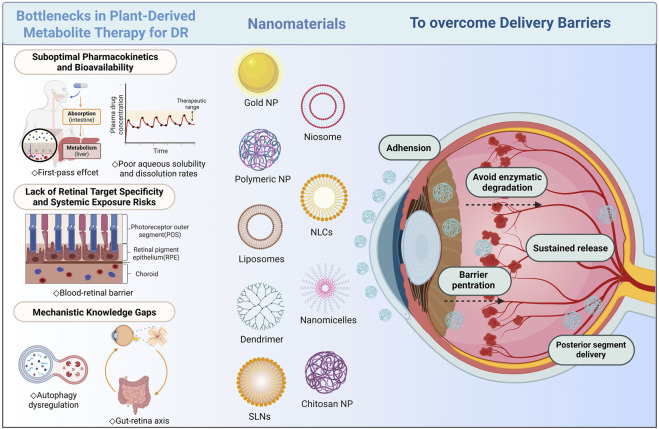
Nanocarrier-mediated strategies for overcoming pharmacokinetic and ocular delivery barriers in diabetic retinopathy. This schematic illustrates how nanomaterial-enabled delivery systems may address key translational bottlenecks of plant-derived bioactive metabolites in DR, including poor aqueous solubility, rapid metabolism, enzymatic degradation, insufficient ocular residence, limited barrier penetration, and inadequate posterior-segment exposure. Representative nanocarriers include polymeric nanoparticles, liposomes, niosomes, nanostructured lipid carriers, solid lipid nanoparticles, dendrimers, chitosan nanoparticles, and gold nanoparticles. These platforms may improve metabolite stability, enhance corneal or retinal penetration, prolong release, and support posterior-segment delivery. However, improved delivery-related properties should be interpreted as supportive evidence rather than proof of therapeutic efficacy unless confirmed by quantitative ocular pharmacokinetics, biodistribution, safety, and DR-relevant functional endpoints.

Nanomaterial-enabled strategies have emerged as transformative solutions for overcoming the multifaceted delivery barriers confronting plant-derived bioactive metabolites in diabetic retinopathy (DR) therapy (Figure 3). Polymeric nanoparticles, particularly those fabricated from poly (lactic-co-glycolic acid) (PLGA), leverage their biodegradable matrix to encapsulate hydrophobic plant-derived bioactive metabolites within their core, enabling sustained release governed by polymer erosion kinetics (Alkholief et al., 2019). The degradation rate—modulated by molecular weight and lactide-to-glycolide ratio—dictates drug release profiles, with higher lactide content extending release duration to weeks, thereby reducing dosing frequency (Imperiale et al., 2018). Concurrently, chitosan-based nanoparticles exploit inherent mucoadhesive properties via electrostatic interactions with ocular surfaces, enhancing precorneal residence time and promoting paracellular permeability through transient tight-junction modulation. Surface modifications like thiolation or quaternization further augment mucoadhesion and cellular uptake, addressing rapid clearance challenges in conventional eyedrops (Zamboulis et al., 2020).

Critically, comparative analyses reveal divergent performance metrics. Reported drug loading (DL) for paclitaxel-loaded PLGA nanoparticles is around 5%, with encapsulation efficiency (EE) commonly in the 60%–90% range depending on the lactide-to-glycolide ratio, drug–polymer compatibility, and fabrication method (Mir et al., 2017). Surface modification of such PLGA nanoparticles with low amounts of chitosan (CS/PLGA = 0.2, w/w) has been reported to increase DL from ∼5.07% to ∼6.42% and EE from ∼65.8% to ∼80.5%, while higher chitosan loading reverses this DL trend due to the increase in total nanoparticle mass (Lu et al., 2019). Release kinetics also differ: PLGA nanoparticles typically follow an erosion- and diffusion-controlled sustained-release profile, whereas chitosan-coated PLGA nanoparticles display pH-responsive release, with reported drug release approximately 45% greater at pH 5.5 than at pH 7.4, consistent with the pKa (∼6.3) of chitosan amino groups and increased solubility under acidic conditions (Khanal et al., 2016).

Comparative evidence indicates that polymer composition, surface charge, lipid matrix structure, and ligand functionalization substantially influence loading, release, ocular residence, and safety. PLGA systems are well suited for sustained release but may show burst release and acidic degradation microenvironments. Chitosan-based systems improve mucoadhesion and precorneal residence but may increase charge-dependent irritation. Liposomes, SLNs, and NLCs improve solubilization of lipophilic metabolites, whereas nanoemulsions require careful surfactant selection because ocular irritation thresholds remain incompletely defined. Dendrimers and exosomes offer targeting or barrier-crossing advantages but raise additional concerns regarding toxicity, heterogeneity, manufacturing reproducibility, and regulatory classification.

Lipid-based nanocarriers present distinct advantages for lipophilic plant-derived bioactive metabolites. Liposomes, comprising phospholipid bilayers surrounding aqueous compartments, solubilize lipophilic metabolites such as silymarin or curcumin within their lipid domains, improving dissolution and bioavailability (Ranjbar et al., 2023). Their structural similarity to cell membranes facilitates lymphatic uptake, bypassing hepatic first-pass metabolism—a critical advantage for metabolites with poor oral bioavailability (Krishnaiah, 2010). Solid lipid nanoparticles (SLNs) and nanostructured lipid carriers (NLCs) further optimize stability and payload capacity; SLNs utilize solid lipids to prolong ocular residence but face limitations like drug expulsion during crystallization, while NLCs incorporate liquid lipids to create imperfect matrices that enhance drug loading and inhibit recrystallization (Lv et al., 2024b). Both systems demonstrate enhanced corneal penetration and sustained release, with NLCs showing superior biocompatibility and reduced irritancy (Wang et al., 2024).

Beyond these platforms, nanoemulsions—thermodynamically unstable but kinetically stable oil-in-water systems—improve solubility and precorneal retention through interaction with tear-film lipids (Qamar et al., 2019). However, surfactant-induced ocular irritation and thermodynamic instability necessitate careful excipient selection (Jacob et al., 2022). Dendrimers, highly branched nanostructures with modifiable surfaces, enable precise active targeting through ligand conjugation, with internal cavities suitable for encapsulating hydrophobic metabolites such as genistein (Kesharwani and Iyer, 2015; Chanphai and Tajmir-Riahi, 2018). Higher-generation dendrimers (e.g., PAMAM G4) have been reported to enhance cellular uptake relative to lower generations through multivalent ligand presentation and increased surface charge density, although charge-dependent toxicity and dose-dependent effects remain a key concern. Functionalization strategy further influences targeting efficiency: folate-mediated conjugation can improve receptor-specific cellular accumulation in folate-receptor-positive cells (Lv et al., 2017), whereas peptide modifications enable enzyme-responsive release through cleavable linkers. However, most quantitative data supporting these effects derive from tumor or non-ocular cell models, so their translational relevance to retinal cell types and DR-specific receptor expression patterns requires direct ocular validation (Kannan et al., 2023).

Niosomes (non-ionic surfactant vesicles) enhance transcellular permeability via fusion with cell membranes or endocytosis, though enzymatic digestion of the blood-retinal barrier may be required for optimal macromolecular delivery (Durak et al., 2020). A comparative overview of the key performance metrics for these nanocarrier platforms is provided in Table 4, highlighting their distinct advantages for ocular application.

**TABLE 4:** Comparative profile of major nanocarrier platforms for plant-derived bioactive metabolite delivery in DR.

Platform	Suitable metabolite type	Main delivery advantage	Key ocular barrier addressed	Release/Stability profile	Major safety concern	Scale-up/Regulatory challenge	Translational interpretation
PLGA nanoparticles	Hydrophobic or moderately hydrophobic metabolites	Biodegradable matrix; tunable sustained release; protection from degradation	Precorneal clearance, limited residence time, enzymatic degradation	Polymer erosion enables sustained release, but burst release and acidic degradation microenvironments may occur	Polymer degradation acidity, residual solvent, inflammatory response	Solvent removal, batch reproducibility, sterilization, particle-size control	Useful for sustained delivery, but retinal targeting requires ocular PK and safety validation
Chitosan nanoparticles	Hydrophilic or moderately hydrophobic metabolites; mucoadhesive ocular formulations	Mucoadhesion, enhanced precorneal residence, transient tight-junction modulation	Tear clearance, corneal epithelial barrier	Surface charge improves adhesion but may destabilize formulation depending on pH and ionic strength	Charge-dependent irritation or cytotoxicity	Polymer variability, charge control, sterilization, viscosity control	Useful for topical delivery, but irritation and barrier integrity must be evaluated
Liposomes	Lipophilic and amphiphilic metabolites	Phospholipid bilayer improves solubilization and membrane interaction	Corneal epithelial barrier, poor aqueous solubility	Biocompatible but prone to leakage, lipid oxidation, and storage instability	Lipid oxidation products, surfactant/excipient irritation	Cold-chain/storage, sterilization, leakage control, scale-up reproducibility	Clinically familiar platform, but stability and ocular exposure remain critical
SLNs	Lipophilic metabolites	Solid lipid matrix improves stability and ocular residence	Poor solubility, rapid clearance	More stable than emulsions, but drug expulsion during lipid crystallization may occur	Lipid/excipient irritation, limited loading for some metabolites	Polymorphic transition, sterilization, particle-size consistency	Supportive for lipophilic payloads but requires release and stability testing
NLCs	Lipophilic or co-loaded lipophilic/hydrophilic metabolites	Imperfect lipid matrix improves drug loading and reduces recrystallization	Poor solubility, limited residence, formulation instability	Better loading than SLNs, sustained release possible	Surfactant irritation, lipid oxidation	Complex formulation optimization, scale-up, sterilization	Promising for co-encapsulation, but synergy claims require free-metabolite and blank-carrier controls
Nanoemulsions	Highly lipophilic metabolites	Solubilization, tear-film interaction, improved precorneal retention	Poor aqueous solubility, precorneal clearance	Kinetically stable but thermodynamically unstable; surfactant ratio is critical	Surfactant-induced ocular irritation	Droplet-size control, phase stability, sterilization, preservative compatibility	Useful for topical solubilization, but ocular irritation thresholds must be defined
Exosomes	Labile metabolites or biologically active cargo	Endogenous membrane structure; potential barrier crossing and immune compatibility	Potential BRB penetration, intracellular delivery	Biologically stable in some contexts but highly source-dependent	Immunogenicity, unwanted biological cargo, batch heterogeneity	Low yield, cargo-loading variability, source characterization, unclear regulatory pathway	High conceptual potential, but clinical translation is limited by reproducibility and regulation
Dendrimers	Hydrophobic metabolites or ligand-targeted payloads	Precise surface functionalization, multivalent ligand display, receptor-mediated targeting	Cellular uptake barriers, receptor-specific retinal delivery	Highly tunable; release depends on surface/linker design	Generation-, charge-, and dose-dependent toxicity	Complex synthesis, purification, characterization, regulatory uncertainty	Useful for active targeting, but safety margin must be carefully established
Hybrid or stimuli-responsive systems	Combination therapies or stage-specific payloads	Spatiotemporal release triggered by pH, ROS, enzymes, light, or magnetic fields	Off-target exposure, insufficient site-specific release	Potentially programmable but more complex and less standardized	Trigger-related tissue injury, unpredictable release	Complex manufacturing, validation of trigger reproducibility, regulatory complexity	Promising for precision therapy but currently early-stage

Selection of a nanocarrier should be guided by metabolite solubility, chemical stability, intended route of administration, target retinal compartment, release profile, ocular safety, sterilization compatibility, and scale-up feasibility. Therefore, improved solubility or cellular uptake alone should not be interpreted as therapeutic superiority without quantitative ocular biodistribution, DR-relevant efficacy endpoints, and repeated-dose safety data.

Despite these advances, challenges persist. Initial burst release in PLGA systems, aggregation during storage, limited drug-loading capacity, and purification hurdles demand further innovation (Lv et al., 2024b). Future efforts should prioritize surface engineering to enhance BRB penetration and stimuli-responsive designs for spatiotemporal control of plant-derived bioactive metabolite release (Prajapati et al., 2016). Collectively, nanocarriers bridge the gap between preclinical screening and therapeutic efficacy, offering a versatile toolkit for targeted, sustained delivery of plant-derived metabolite therapeutics in DR management.

### 4.2 Ocular barrier penetration enhancement

Size Optimization (<500 nm) remains a cornerstone strategy for facilitating transcorneal permeation. Nanoparticles within this size range exploit endocytic pathways in corneal epithelial cells, enabling intracellular trafficking and improved drug bioavailability (Narayana et al., 2021). Recent evidence suggests that particles below 100 nm exhibit superior diffusion through the gel-like vitreous humor toward retinal targets, though optimal size is barrier-specific: while sub-20 nm particles demonstrate multi-layer corneal penetration in some models (Blass, 2010), surface chemistry often outweighs size as a determinant for stromal permeation. For instance, 43–69 nm PEGylated silica nanoparticles penetrated corneal stroma effectively, whereas similarly sized thiolated particles adhered superficially regardless of dimension (Mun et al., 2014). This highlights that size optimization must be contextually integrated with surface engineering.

Surface Engineering, particularly PEGylation, addresses ocular clearance mechanisms by minimizing mucoadhesive entrapment and reducing opsonization. Dense PEG coatings create a hydrophilic, sterically stabilized surface that resists interactions with mucin glycoproteins, thereby prolonging precorneal residence time and enhancing transcellular transport (Moiseev et al., 2022). For example, PLA-PEG nanoparticles significantly increased acyclovir levels in aqueous humor compared to unmodified counterparts in rabbit models by mitigating mucociliary clearance. Advanced derivatives like maleimide-functionalized PEGylated liposomes further optimize mucoadhesion-retention balance, demonstrating sustained drug release while maintaining penetration efficiency. However, contradictory findings exist—some studies note reduced phagocytic uptake of PEGylated systems (Suk et al., 2016), underscoring the need for disease-specific surface tailoring.

Route-Specific Optimization balances therapeutic efficacy with patient burden through material design adaptations. Non-invasive topical delivery benefits from nanomaterials enhancing corneal retention and penetration. Hydrogel-integrated nanoparticles and cyclodextrin-modified carriers show promise in improving bioavailability for anterior segment delivery, yet posterior segment targeting remains challenging due to sequential barriers. Here, invasive routes (intravitreal, periocular) offer superior bioavailability: suprachoroidal injection of sodium fluorescein achieved approximately 25-fold higher peak choroid-retina concentrations than intravitreal administration and approximately 36-fold higher than posterior subconjunctival administration in a rat ocular fluorophotometry study (Tyagi et al., 2012). Nanocarriers further augment these approaches—PEGylated DNA nanoparticles exhibited enhanced vitreal mobility and retinal transfection without efficiency loss, while sustained-release systems (e.g., PLGA nanoparticles) reduce injection frequency (Zeng et al., 2019). The emerging paradigm favors tailoring nanocarrier properties (size, charge, ligand decoration) to administration routes: cationic nanoparticles for topical adhesion, stealth-coated variants for intravitreal durability, and stimuli-responsive systems for periocular depots.

These strategies collectively address DR’s chronic management needs. PEGylation and sub-100 nm sizing synergize to overcome vitreous diffusion barriers, while route-specific designs mitigate risks—e.g., topical nanoemulsions reduce systemic side effects versus oral drugs, and long-acting intravitreal implants decrease procedural complications. Future directions include multi-barrier penetrating designs (e.g., mucus-inert yet cornea-permeating nanoparticles) and hybrid administration protocols leveraging initial invasive loading followed by topical maintenance (Liu X. et al., 2024).

### 4.3 Targeted and sustained retinal delivery strategies

The development of nanomaterial-enabled delivery systems for plant-derived bioactive metabolites in diabetic retinopathy (DR) treatment requires sophisticated strategies to overcome ocular barriers, with active retinal targeting representing a pivotal approach. Ligand functionalization, particularly using cyclic Arg-Gly-Asp (cRGD) peptides and cholera toxin subunit B (CTB), exploits receptor-mediated endocytosis to enhance cellular uptake in specific retinal cells. cRGD peptides selectively bind to integrins αvβ3/αvβ5, which are overexpressed on retinal endothelial cells during pathological angiogenesis, facilitating targeted delivery to the retinal pigment epithelium (RPE) and neovascular sites. Preclinical studies demonstrate that cRGD-modified nanocarriers significantly improve RPE targeting efficiency and cellular internalization compared to non-functionalized counterparts, as confirmed by enhanced inhibition of VEGF expression in DR rodent models (Liu et al., 2020). Similarly, CTB leverages its high affinity for monosialotetrahexosylganglioside (GM1) receptors enriched on retinal ganglion cells (RGCs), enabling precise RGC targeting. This strategy has shown efficacy in attenuating RGC degeneration via receptor-mediated endocytosis, as evidenced by reduced excitotoxic damage and improved neuroprotection in glaucoma models, with potential applicability to DR-associated neurodegeneration (Lu et al., 2014).

Concurrently, sustained and controlled release mechanisms are integral to maintaining therapeutic efficacy while minimizing dosing frequency. Poly (lactic-co-glycolic acid) (PLGA)-based matrices exemplify this approach, leveraging their tunable degradation kinetics to prolong drug release. The lactide-to-glycolide ratio in PLGA dictates hydrolysis rates, allowing release profiles spanning days to months. For instance, bevacizumab-loaded PLGA/PCADK microspheres prepared by solid-in-oil-in-water emulsification have been reported to provide a comparatively low initial burst followed by sustained *in vitro* and *in vivo* release of bioactive bevacizumab for approximately 50 days, outperforming conventional formulations and supporting reduced intravitreal injection frequency (Liu et al., 2019). Recent advancements include hot-melt-extruded PLGA implants for dexamethasone, which offer reproducible, long-term release with reduced burst effects, further optimizing therapeutic exposure (Zhang et al., 2021). Such systems mitigate the need for frequent intravitreal injections, enhancing patient compliance and reducing iatrogenic complications.

Baicalein is supported by relatively stronger *in vivo* retinal evidence. In STZ-diabetic rats, baicalein attenuated microglial activation, reduced retinal IL-18, TNF-α and IL-1β expression, suppressed Müller-cell GFAP and VEGF upregulation, and reduced vascular abnormalities and ganglion-cell loss at 24 weeks (Yang et al., 2009); In retinal ischemia models, baicalein also downregulated HIF-1α, VEGF and MMP-9 while upregulating HO-1 and Bcl-2 (Chao et al., 2013). Curcumin is a less straightforward case: although in retinal cell models curcumin reduces NF-κB signalling, IL-6, TNF-α and oxidative-stress markers (Premanand et al., 2006; Yang et al., 2018), it is also a recognised PAINS scaffold prone to redox cycling, fluorescence interference and aggregation. The cytokine and NF-κB nodes assigned to curcumin in Table 3 should therefore be read alongside its assay-interference profile, and any DR-specific *in vitro* readout should be supported by orthogonal assays, dose–response data, and *in vivo* validation before being treated as confirmatory.

Despite these innovations, nanocarriers face significant translational challenges. Biocompatibility and toxicity concerns arise from material-specific interactions; for example, positively charged nanoparticles may disrupt negatively charged cell membranes, while smaller particles (e.g., 15 nm silica NPs) exhibit higher retinal cytotoxicity than larger variants (50 nm) due to enhanced cellular penetration (Zhao et al., 2017). Immunogenicity is another critical hurdle, as surface ligands like CTB or synthetic polymers can trigger immune recognition. Strategies to circumvent this include PEGylation to reduce opsonization and the development of “stealth” nanoparticles with surface modifications that minimize macrophage uptake (Zhang and Diamond, 2006). Long-term biodistribution and clearance issues also persist, as nanocarriers may accumulate in ocular tissues, potentially inducing chronic inflammation or oxidative stress. Comprehensive biocompatibility assessments—evaluating cell viability, inflammatory cytokine release, and histopathological changes—are essential but remain inadequately standardized for ocular applications (Perlman et al., 2008).

Scale-up complexity further impedes clinical translation. Multifunctional nanocarriers integrating targeting ligands, controlled-release polymers, and therapeutic payloads require intricate manufacturing processes. Reproducibility is compromised by batch-to-batch variability in particle size, polydispersity, and ligand orientation. Scaling bottom-up synthesis (e.g., emulsion-based PLGA NP production) necessitates stringent control over solvent removal and purification, while top-down methods face limitations in achieving nanoscale precision. Regulatory frameworks for such complex products are still evolving, with few established quality control metrics for critical parameters like drug release kinetics or *in vivo* degradation profiles (Singh and Ahmad, 2011).

In conclusion, while ligand-functionalized nanocarriers and PLGA-based sustained release systems offer promising solutions for targeted plant-derived bioactive metabolite delivery in DR, addressing biocompatibility, immunogenicity, long-term safety, and scalable production is imperative for clinical viability. Future research must prioritize standardized biocompatibility protocols, novel immunomodulatory coatings, and scalable manufacturing technologies to bridge the gap between preclinical efficacy and clinical application.

## 5 Preclinical screening and efficacy evaluation frameworks

To provide a structured overview of the preclinical screening strategy, the main *in vitro*, *ex vivo*, *in vivo*, pharmacokinetic, and safety techniques relevant to nano-enabled plant-derived metabolite therapeutics for DR are summarized in Table 5.

**TABLE 5:** Integrated preclinical screening and validation techniques for nanomaterial-enabled delivery of plant-derived bioactive metabolites in diabetic retinopathy.

Screening tier	Representative models/Techniques	Main endpoints	What the method can support	Key limitations/Interpretation
Basic formulation screening	Particle size/PDI, zeta potential, morphology, encapsulation efficiency, drug loading, *in vitro* release, colloidal stability, sterility/endotoxin testing	Formulation quality, release profile, reproducibility	Confirms whether the nanoformulation is sufficiently defined for biological testing	Does not prove ocular delivery or therapeutic efficacy
Retinal-cell *in vitro* assays	Retinal endothelial cells, RPE cells, Müller glia, retinal ganglion cells, microglia; high-glucose, hypoxia, oxidative-stress models	Cell viability, ROS, cytokines, VEGF, apoptosis, autophagy markers, tight-junction proteins	Provides early mechanistic and cytotoxicity evidence	Single-cell models cannot reproduce ocular barriers, tissue distribution, or chronic DR pathology
Barrier and permeability models	Transwell RPE/retinal endothelial monolayers, TEER, fluorescein-dextran permeability, iPSC-derived inner BRB models, microfluidic BRB chips	TEER, permeability coefficient, ZO-1/occludin integrity, carrier transport	Evaluates barrier protection and nanocarrier penetration potential	Barrier crossing *in vitro* does not necessarily predict retinal exposure *in vivo*
*Ex vivo*/alternative irritation models	HET-CAM, isolated ocular tissues, 3D corneal models, retinal organoids	Hemorrhage/coagulation/lysis score, tissue penetration, cytokine response, epithelial or retinal toxicity	Useful for acute irritation and intermediate safety screening	HET-CAM cannot model chronic retinal inflammation, repeated-dose immunogenicity, or diabetic microvascular injury
*In vivo* DR efficacy models	STZ-induced diabetic rodents, db/db or Akita models, OIR model	Vascular leakage, neovascularization, retinal thickness, pericyte loss, inflammatory markers, ERG/OCT/FFA readouts	Provides disease-relevant efficacy evidence	STZ mainly models early hyperglycemia-related injury; OIR models ischemia-driven neovascularization but not chronic diabetes
Ocular PK/biodistribution	LC-MS/MS, fluorescence imaging, radiolabeling, MALDI imaging mass spectrometry, retina/choroid/vitreous/plasma concentration-time profiling	Cmax, Tmax, AUC, half-life, retina-to-plasma ratio, tissue retention	Essential for claims of retinal targeting or improved posterior-segment exposure	Improved plasma exposure or cellular uptake alone is insufficient
Safety and translational readiness	Repeated-dose ocular toxicity, histology, ERG, IOP, inflammatory cytokines, systemic liver/kidney markers, immunogenicity, sterilization/stability after processing	Ocular tolerance, chronic toxicity, immune response, formulation stability	Supports translational readiness and route-specific safety	Acute viability or irritation assays alone cannot establish clinical safety

AUC, area under the curve; BRB, blood-retinal barrier; ERG, electroretinography; FFA, fundus fluorescein angiography; HET-CAM, hen’s egg test on the chorioallantoic membrane; IOP, intraocular pressure; LC-MS/MS, liquid chromatography–tandem mass spectrometry; MALDI-MSI, matrix-assisted laser desorption/ionization mass spectrometry imaging; OCT, optical coherence tomography; PDI, polydispersity index; TEER, transepithelial/transendothelial electrical resistance.

### 5.1 *In vitro* screening models

The preclinical screening and efficacy evaluation of nanomaterial-enabled plant-derived bioactive metabolite delivery for diabetic retinopathy (DR) requires a multi-tiered approach that integrates physiologically relevant *in vitro* models with mechanistic therapeutic assessments. Initial screening leverages cell-based assays utilizing primary or stem cell-derived retinal cell types to evaluate both biocompatibility and therapeutic mechanisms. Retinal endothelial cells (RECs), particularly those derived from human induced pluripotent stem cells (iPSCs) via Wnt/β-catenin activation, provide a renewable source for modeling the inner blood-retina barrier (iBRB) dysfunction central to DR. These iPSC-RECs exhibit transcriptomic and functional fidelity, including expression of tight junction proteins and responsiveness to diabetic stressors, enabling robust assessment of nanocarrier effects on vascular leakage and angiogenesis (Lin et al., 2025). Co-culture systems incorporating Müller glia are critical, as these cells secrete neuroprotective factors (e.g., osteopontin/SPP1, basigin/BSG) that enhance retinal ganglion cell (RGC) survival. Conditioned media from Müller glia or direct co-culture with RGCs significantly reduces apoptosis and promotes neurite outgrowth *in vitro*, providing a platform to evaluate nanocarrier-delivered metabolites like berberine or epigallocatechin-3-gallate (EGCG) for neuroprotection (Ruzafa et al., 2018).

Comprehensive cytokine modulation profiling is integral to anti-inflammatory evaluation. Multiplex bead array assays quantify shifts in key cytokines (IL-1β, IL-6, IL-8, TNF-α, VEGF) within retinal cell supernatants after metabolite-loaded nanocarrier treatment. Comprehensive cytokine profiling, including multiplex bead array assays, can quantify DR-relevant inflammatory mediators such as IL-1β, IL-6, IL-8, TNF-α, and VEGF in ocular samples or retinal-cell supernatants. Clinical studies of proliferative diabetic retinopathy have confirmed altered aqueous and vitreous inflammatory cytokine profiles, supporting the relevance of these endpoints for preclinical evaluation (Mason et al., 2022). In nanocarrier-based screening studies, cytokine suppression by curcumin- or resveratrol-loaded polymeric systems and VEGF/HIF-1α modulation by quercetin-loaded lipid systems should be interpreted as formulation-level evidence unless supported by primary dose-response, ocular pharmacokinetic, and *in vivo* validation data (Ye et al., 2024; Wei et al., 2020).

Autophagy modulation screening employs LC3-II immunoblotting and fluorescent microscopy (e.g., GFP-LC3 transfection) to track autophagic flux. Natural products like gypenoside XVII (Gyp-17) in chitosan-alginate nanocarriers activate AMPK/mTOR pathways in Müller cells, increasing autophagosome formation by 40% and mitigating high glucose-induced apoptosis, while metabolites such as norkurarinone inhibit excessive autophagy via PI3K/AKT to preserve RPE viability (Liu F. et al., 2024). Resveratrol is the most extensively profiled DR-relevant example: in STZ-diabetic mice and primary retinal ganglion cells under high-glucose stress, resveratrol activated the Nrf2/HO-1 antioxidant axis, reduced MDA, and improved electroretinographic responses (Chen et al., 2020); in rat retinal Müller cells, resveratrol restored autophagic flux through the miR-29b/SP1 axis with concurrent upregulation of Beclin-1 and LC3-II and suppression of apoptosis (Wan et al., 2025); and across *in vitro* and *in vivo* DR studies, resveratrol consistently shifted Bcl-2/Bax balance toward survival in parallel with SIRT1/AMPK and Nrf2/HO-1 activation (Kaštelan et al., 2025). These pathway-level findings underpin the molecular nodes assigned to resveratrol in Table 3, but autophagic flux is rarely quantified using paired bafilomycin/chloroquine controls and ocular pharmacokinetic data are limited, so the strength of evidence remains preclinical. Apoptosis assays (TUNEL, caspase-3/9 activity) complement these studies and have been used to characterize how nanocarrier-delivered metabolites may modulate the balance between pro-survival autophagy and apoptosis, although direct evidence that these readouts predict therapeutic-window improvement in DR remains limited (Hu et al., 2023).

To model physiological barriers, corneal/retinal barrier penetration is evaluated using Transwell assays with optimized pore sizes (3.0 μm) and TEER measurements. Polymeric nanocarriers functionalized with cell-penetrating peptides (e.g., penetratin) or adenosine triphosphate (ATP) disrupt tight junctions (ZO-1, occludin) in RPE monolayers, enhancing paracellular transport 9-fold. ATP-co-administered chitosan-pluronic nanocarriers achieve deeper retinal penetration in *ex vivo* models by reducing electrostatic interactions with vitreal glycosaminoglycans (Kwon et al., 2021; Gabai et al., 2023). Advanced microphysiological systems incorporating iPSC-RECs, pericytes, and Müller glia under fluidic shear stress further replicate the iBRB, allowing real-time quantification of nanocarrier permeability and retention (Cimino et al., 2024).

Integrated preclinical efficacy frameworks bridge *in vitro* screening with *in vivo* relevance. Nanoparticles are prioritized based on: (1) cytotoxicity thresholds (IC50 > 100 μg/mL in retinal cells); (2) anti-angiogenic potency (VEGF suppression >60%); (3) barrier penetration (Papp > 1 × 10^−6^ cm/s); and (4) cytokine/autophagy modulation specificity. Gold nanoparticles conjugated with epigallocatechin (AuNP-EGCG) exemplify this, demonstrating 80% VEGF reduction in RECs, sustained RGC protection in co-culture, and >12-h retinal retention in translational models (Tang et al., 2022). Such multi-parametric validation ensures metabolite-loaded nanocarriers advance toward *in vivo* testing with mechanistic rigor and therapeutic promise.

### 5.2 *In vivo* disease models (rodents)

The preclinical screening and efficacy evaluation of nanomaterial-enabled plant-derived bioactive metabolite delivery for diabetic retinopathy (DR) relies heavily on robust *in vivo* rodent models that recapitulate distinct pathological phases of the disease. Two cornerstone models—Streptozotocin (STZ)-induced diabetic rodents and Oxygen-Induced Retinopathy (OIR)—serve complementary roles in modeling hyperglycemia-driven neurodegeneration/vascular leakage and hypoxia-triggered pathological neovascularization, respectively. The STZ model, induced by intraperitoneal administration of 40–60 mg/kg STZ, selectively destroys pancreatic β-cells via GLUT2 transporter-mediated uptake, leading to insulin-dependent diabetes mellitus (T1DM) and subsequent retinal pathology (Polewik et al., 2023). This model faithfully mirrors early non-proliferative DR (NPDR) in humans, exhibiting hallmark features such as blood-retinal barrier (BRB) breakdown, increased vascular permeability, pericyte loss, capillary degeneration, microaneurysm formation, retinal thinning (particularly in the inner plexiform and nuclear layers), and ganglion cell apoptosis (Amadio et al., 2010). Molecular alterations include upregulated vascular endothelial growth factor (VEGF), intercellular adhesion molecule-1 (ICAM-1), and aldose reductase activity, alongside downregulated pigment epithelium-derived factor (PEDF) and occludin expression, confirming multifaceted retinal damage (Duan et al., 2013). While STZ rats develop consistent NPDR features within 3–6 months of hyperglycemia (>150 mg/dL), limitations include its primary representation of T1DM (lacking insulin resistance typical of T2DM), instability/reversibility of diabetes with insulin therapy, gender-based susceptibility variations (female mice exhibit resistance), and an inability to progress to proliferative DR (PDR) due to the model’s short lifespan and the protracted nature of human PDR development (Lai and Lo, 2013).

In contrast, the OIR model, particularly the murine variant, is indispensable for studying ischemia-driven proliferative stages of DR. Neonatal mice (postnatal day 7, P7) are exposed to hyperoxia (75% O_2_ for 5 days), causing vaso-obliteration (VO), followed by a return to normoxia (room air at P12), inducing profound retinal hypoxia and robust preretinal neovascularization (NV) peaking around P17. This hypoxia triggers molecular cascades analogous to human PDR and retinopathy of prematurity (ROP), including VEGF upregulation, inflammatory cell infiltration, and aberrant vascular growth (Vähätupa et al., 2020; Maurya et al., 2024). Recent refinements include the accelerated OIR protocol (85% O_2_ from P8-P11), which induces comparable NV but greater vascular regression and faster recovery, with better-preserved neuroretinal function assessed by electroretinography (ERG), offering a more rapid and reproducible alternative (Villacampa et al., 2017; Mitton et al., 2019). Advanced quantification techniques, such as fully automated deep learning segmentation of VO/NV areas and machine learning-based vascular assessment, enhance objectivity and throughput in evaluating therapeutic efficacy (Xiao et al., 2017; Mazzaferri et al., 2018). Furthermore, longitudinal monitoring using fluorescein angiography (FFA) in live OIR mice allows dynamic assessment of vascular remodeling during recovery phases, providing deeper insights into revascularization kinetics (Ma and Li, 2021). While OIR excels in modeling PDR-like neovascularization and ischemia, it does not replicate the chronic hyperglycemic milieu or metabolic dysregulation inherent to DR (Cammalleri et al., 2024).

The integration of nanomaterial-based delivery systems (e.g., polymeric nanoparticles, lipid carriers, hydrogels) into these preclinical frameworks aims to overcome the poor bioavailability, solubility, and retinal targeting limitations of plant-derived bioactive metabolites. Nanocarriers facilitate sustained release, enhanced corneal/retinal penetration, and targeted delivery to pathological sites (e.g., neovascular tufts in OIR or leaky vasculature in STZ models) (Cheng et al., 2017). For instance, PLGA-PEG-PLGA thermosensitive hydrogels and cell membrane-coated nanoparticles have shown promise in improving ocular residence time and biocompatibility (Chen Q. et al., 2024). Efficacy evaluation in STZ models focuses on nanocarriers’ ability to mitigate hyperglycemia-induced oxidative stress, inflammation (e.g., reducing leukostasis and ICAM-1), and BRB dysfunction, while in OIR models, success is measured by the suppression of pathological NV and promotion of physiological revascularization (Wu J. H. et al., 2020). Notably, dual-targeting approaches leveraging both models, such as the ROCK inhibitor AMA0428, demonstrate efficacy in reducing vascular leakage (STZ) and neovascularization (OIR), highlighting the synergistic value of these frameworks in comprehensive DR drug development (Hollanders et al., 2017). However, translational challenges persist, including nanocarrier-specific toxicity, insufficient drug-loading capacity, and the need for GLP-compliant ocular toxicity studies under evolving regulatory guidance emphasizing rigorous safety, species-specific suitability, and advanced functional/imaging endpoints (Fosse et al., 2023; Vezina, 2013).

### 5.3 Advanced efficacy assessment methods

The preclinical screening and efficacy evaluation frameworks for nanomaterial-enabled delivery of plant-derived bioactive metabolites in diabetic retinopathy (DR) leverage advanced multimodal methodologies to comprehensively assess therapeutic outcomes. Functional evaluations employ electroretinography (ERG) to quantify neuronal dysfunction, with recent advances demonstrating the clinical utility of flicker ERG for its portability and rapid assessment capabilities in detecting early photoreceptor and bipolar cell impairment (McAnany et al., 2022). Multifocal ERG (mfERG) has emerged as a predictive tool for vascular abnormalities, while deep learning-powered automated analysis systems (e.g., 2024 models achieving 77.59% F1-scores) significantly enhance throughput and objectivity in detecting retinal functional anomalies (Giap et al., 2024). Complementing ERG, fundus fluorescein angiography (FFA) remains indispensable for evaluating vascular leakage dynamics. Innovations like FFA-GPT (2024), which integrates multimodal transformers and large language models, automate image interpretation and enable real-time quantification of dye leakage patterns, thereby enhancing sensitivity for microaneurysm detection in DR (Chen X. et al., 2024). Furthermore, super-resolution networks address FFA’s spatial resolution limitations, facilitating precise visualization of nanomaterial-targeted vascular repair (Velumani et al., 2021). Optical coherence tomography (OCT), with axial resolutions now reaching 2 μm, provides high-fidelity cross-sectional imaging of retinal thickness alterations. Spectral-domain OCT protocols exhibit > 92% reproducibility in macular thickness measurements, enabling longitudinal tracking of nanoparticle-mediated edema resolution and neural layer preservation (Sull et al., 2010).

Morphometric and histopathological analyses deliver granular structural insights. Acellular capillary quantification, standardized via masked microscopy across mid-retinal fields (×40–400 magnification), calculates capillary-sized tubes lacking nuclei per mm^2^ retinal area, with recent protocols excluding strands <20% of adjacent capillary diameter to minimize artifacts (Bapputty et al., 2019). Pericyte loss, a hallmark of DR, is evaluated using COL4 immunostaining—validated as a robust marker in 2023—with density reported per mm^2^ capillary area (Ding et al., 2020). Neovascular nuclei are quantified via OCT angiography (OCTA), where widefield swept-source OCTA (12 × 12 mm scans) achieves 73%–100% detection rates for pathological angiogenesis, outperforming fluorescein angiography in spatial resolution (Lu et al., 2022). Ganglion cell density mapping employs deep learning tools like GanglionNet, which automates cell counting in histological sections with 97.49% accuracy using OCT-derived retinal ganglion layer thickness and spatial summation algorithms (Alom et al., 2021). Inflammatory infiltrates are spatially resolved via z-score normalized retinal maps, integrating OCT thickness measurements and semi-quantitative vitreal infiltration scoring to correlate nanoparticle efficacy with leukostasis reduction (Bradley et al., 2021).

Molecular biomarker profiling elucidates mechanistic pathways modulated by nanoformulations containing plant-derived bioactive metabolites. VEGF inhibition efficacy is assessed through ELISA of retinal homogenates, with recent studies confirming its upstream regulation of the PKC/ET/NF-κB/ICAM-1 cascade; GFI09203X (PKCβ2 inhibitor) reduces VEGF-driven vascular leakage by 85.6% in STZ-diabetic models (Zhang et al., 2022). ICAM-1 overexpression, quantified via RNase protection assays and immunofluorescence, is directly induced by VEGF and attenuated by targeted nano-delivery, suppressing leukocyte adhesion (Lu et al., 1999). TNF-α′s role in caspase-mediated apoptosis is validated in TNF-R1-deficient mice, where etanercept (TNF-α inhibitor) reduces pericyte loss by >48% via suppression of caspase-3/8 activation (Joussen et al., 2009). Caspase activity assays (e.g., fluorometric substrate cleavage at 490/525 nm) in retinal ganglion cells quantify anti-apoptotic effects, while caspase-7 ablation studies reveal its modulation of unfolded protein responses in neurodegeneration (Ma et al., 2022). LC3-II autophagy flux is monitored via immunoblotting of the LC3-II/LC3-I ratio, where phosphatidylethanolamine conjugation serves as a definitive marker of autophagosome formation; dysregulation correlates with DR progression and light-induced degeneration (Chen et al., 2013). These integrated frameworks thus provide a rigorous, multidimensional foundation for validating nanomaterial-enabled plant-derived metabolite therapeutics in DR preclinical models.

### 5.4 Pharmacokinetic and biodistribution studies

In the preclinical screening and efficacy evaluation of nanomaterial-enabled plant-derived bioactive metabolite delivery for diabetic retinopathy (DR), pharmacokinetic (PK) and biodistribution studies constitute a critical framework for understanding therapeutic behavior *in vivo*. These investigations employ sophisticated bioimaging-guided tracking methodologies, where nanocarriers are conjugated with fluorescent probes (e.g., silicon nanoparticles functionalized with RGD peptides (Ganapathy et al., 2020)), isotope labels, or photoacoustic agents (e.g., gold nanoparticle-enhanced nanodiamonds (Zhang et al., 2012)). This enables real-time, non-invasive spatial and temporal monitoring of ocular distribution and retention using multimodal imaging platforms such as fluorescence imaging, MRI, PET, and CT (Zhang et al., 2024). For instance, recent advancements include the development of biodegradable adhesive fluorescent nanoprobes targeting molecular markers like VEGFR-2 in the retinal microvasculature. These probes facilitate early DR detection by crossing the blood-retinal barrier, binding specifically to endothelial cells, and adhering to leukocytes, allowing dynamic visualization of pathological changes (Wang et al., 2024). Such techniques provide invaluable insights into the ability of nanocarriers to overcome anatomical barriers and achieve targeted delivery to posterior segment tissues like the retina and choroid, a significant challenge in DR therapy.

Complementing these imaging studies, rigorous quantitative analysis of drug concentrations in specific ocular substructures is imperative. State-of-the-art bioanalytical methods, particularly high-throughput liquid chromatography-tandem mass spectrometry (LC-MS/MS), allow simultaneous quantification of multiple drug candidates across diverse ocular matrices (cornea, aqueous humor, vitreous, retina/choroid) and plasma. Cassette dosing strategies (e.g., N-in-one assays) enable efficient screening of up to 27 drugs with varying physicochemical properties (from hydrophilic sotalol to hydrophobic triamcinolone hexacetonide) within a single analytical run of approximately 18 min. This significantly reduces animal usage while providing sensitive detection limits (e.g., LLOQ of 0.5 fmols for sotalol) and robust reproducibility (%RSD < 20%) (Matter et al., 2022). Tissue-specific drug extraction protocols are optimized to minimize artifacts, ensuring accurate PK parameter determination (e.g., C ∼ max∼, T ∼ max∼, AUC, t∼1/2∼) and calculation of vitreal/plasma concentration ratios, which are crucial for assessing localized therapeutic efficacy and minimizing systemic exposure (Rimpelä et al., 2020). Furthermore, techniques like MALDI imaging mass spectrometry offer spatially resolved quantification directly within tissue sections, correlating drug distribution with histological features, though adaptation for complex ocular matrices remains an active area of refinement (Lynch et al., 2019).

Integrating biodistribution and PK data into preclinical efficacy frameworks allows for meaningful comparisons between nanodelivered plant-derived bioactive metabolites and conventional DR therapeutics. Emerging evidence suggests certain plant-derived bioactive metabolites (e.g., those modulating autophagy and inhibiting apoptosis, such as metabolites derived from *Panax notoginseng*) may achieve favorable retinal bioavailability and sustained retention when encapsulated in nanocarriers like polymeric micelles or liposomes. For example, comparative studies using quantitative LC-MS/MS can reveal whether nanodelivered metabolites like berberine or curcumin analogs exhibit superior retinal accumulation and prolonged residence times compared to standard anti-VEGF biologics or corticosteroids. This integrated approach also elucidates structure-activity relationships (SAR) and structure-property relationships (SPR), guiding the rational design of next-generation nanocarriers. Physiologically based pharmacokinetic (PBPK) modeling, incorporating preclinical biodistribution and clearance data, further aids in predicting human PK profiles and efficacious doses, bridging the gap between animal studies and clinical translation (Rode et al., 2018). While promising, rigorous validation of these frameworks is essential, especially given the current limited clinical evidence for plant-derived bioactive metabolites in DR (Zhang et al., 2018). Future efforts must focus on correlating quantified ocular PK parameters with functional efficacy endpoints (e.g., reduction in retinal vascular leakage, inhibition of neovascularization in DR models) and long-term safety profiles to establish robust preclinical proof-of-concept for nanomaterial-plant-derived bioactive metabolite combinatory therapies.

### 5.5 Safety and toxicity profiling

The preclinical safety and toxicity profiling of nanomaterial-enabled plant-derived bioactive metabolite delivery systems for diabetic retinopathy (DR) demands comprehensive frameworks that address ocular-specific biocompatibility challenges. Ocular irritation and tolerance assessment has evolved significantly beyond traditional Draize testing, with the Hen’s Egg Test on Chorioallantoic Membrane (HET-CAM) emerging as a robust alternative endorsed by European regulatory bodies. This assay leverages the vascular complexity of the chorioallantoic membrane to evaluate hemorrhage, coagulation, and lysis, providing a semiquantitative irritation score while circumventing ethical concerns associated with live-animal testing. Recent studies demonstrate its utility in nanoformulation screening: Smail (2024) validated a brimonidine nanoemulsion (NE) using HET-CAM, revealing that surfactant-propylene glycol combinations caused moderate vascular damage (score: 1.2 ± 0.10), while optimized NEs showed negligible irritation comparable to commercial eye drops. Similarly, Rawat et al. (2024) confirmed the non-irritant nature of nebivolol-loaded hybrid nanoparticles via HET-CAM, emphasizing its predictive correlation with human ocular response. Nevertheless, HET-CAM limitations persist for insoluble or pigmented nanomaterials, driving innovation in 3D corneal epithelium models like Skinovo-Ocular™, which achieved 100% sensitivity in detecting irritants per OECD standards, thereby complementing traditional assays (He et al., 2025).

Although HET-CAM is valuable for rapid screening of acute vascular irritation, it cannot fully model chronic retinal inflammation, repeated-dose immunogenicity, diabetic microvascular dysfunction, or long-term neuroretinal toxicity. Modern 3D corneal/retinal organoids and iPSC-derived blood-retinal barrier models can complement HET-CAM by enabling longer exposure windows, cytokine profiling, barrier-integrity assessment, and cell-type-specific toxicity readouts.

For quantitative toxicodynamic monitoring, the SPOTS (Standardized Preclinical Ocular Toxicology Scoring) system provides critical semiquantitative metrics tailored to DR pathophysiology. This framework integrates evaluations of aqueous flare (indicating blood-retinal barrier disruption), pupillary light reflex impairment (neurovascular compromise), conjunctival swelling (inflammatory response), and intraocular pressure fluctuations (relevant to glaucoma comorbidity). While SPOTS itself requires broader validation in nanotherapy contexts, algorithmic scoring advancements now enhance its precision. Deep learning systems reported by Ting et al. (2017) achieved AUCs >0.93 for referable DR detection, while Slakter’s digital algorithmic severity scoring system quantifies microvascular feature progression—enabling granular tracking of nanoparticle efficacy in reversing capillary non-perfusion or microaneurysms (Slakter et al., 2015). Recent multi-stage classification models further refine this approach; ensemble architectures combining VGGNet and ResNet outputs optimize sensitivity-specificity tradeoffs for early intervention thresholds (Bidwai et al., 2022).

Sterility testing remains non-negotiable for intravitreal/periocular nanoformulations, where microbial contamination risks vision-threatening endophthalmitis. Regulatory-compliant protocols now integrate filtration sterilization (0.22 µm) with challenge testing to validate microbial robustness. Beirampour et al. (2024) demonstrated this for baricitinib-loaded PLGA nanoparticles, confirming resistance to Staphylococcus aureus and Candida albicans after filtration. However, nanomaterial complexity necessitates method adaptations: lipid nanoparticles require thioglycolate/soybean-casein media inoculation per pharmacopeial standards (Satyanarayana et al., 2023), while thermosensitive gels demand aseptic processing from synthesis to packaging (Zielińska et al., 2020). Crucially, sterility validation must accompany stability studies, as nanoparticle aggregation post-sterilization could alter drug release kinetics.

## 6 Regulatory considerations and future translation

### 6.1 Quality control and standardization

The translation of nanomaterial-enabled delivery systems for plant-derived bioactive metabolites for diabetic retinopathy (DR) into clinically viable therapeutics necessitates rigorous adherence to evolving regulatory frameworks, with quality control (QC) and standardization serving as pivotal pillars for ensuring efficacy, safety, and batch-to-batch consistency. Unlike conventional small-molecule drugs, the therapeutic performance of nanoformulations is exquisitely sensitive to physicochemical attributes such as particle size, polydispersity index (PDI), zeta potential, drug loading efficiency, and release kinetics. These parameters directly influence ocular bioavailability, biodistribution, cellular uptake, and ultimately, clinical outcomes. Consequently, comprehensive characterization must be embedded early in formulation design, employing narrow specifications aligned with Quality by Design (QbD) principles endorsed by the FDA and ICH to mitigate costly late-stage reformulations (Jarmila et al., 2024). For nanoformulations containing plant-derived metabolites specifically, the inherent complexity and variability of plant-derived metabolites—even within a single plant species—amplify standardization challenges. Unlike synthetic drugs with well-defined structures, plant-derived metabolites exhibit natural variations in composition and potency, demanding advanced analytical techniques like HPLC and mass spectrometry for authentication, purity verification, and quantification of encapsulated metabolites (Metselaar and Lammers, 2020).

Batch-to-batch reproducibility remains a critical bottleneck in nanomedicine translation. Variations in synthesis conditions during scale-up—such as reactor volume, stirring dynamics, or energy input—can alter particle size distribution, surface morphology, and colloidal stability, leading to inconsistent drug release profiles and therapeutic effects (Shan et al., 2022). This is particularly problematic for complex systems like chitosan-based nanoparticles, where interactions between the polymer and plant-derived bioactive metabolites may unpredictably modify pharmacokinetics and biodistribution. Microfluidic-assisted nanocarrier synthesis has emerged as a promising solution, offering superior control over mixing dynamics, scalability, and reproducibility compared to bulk methods, thereby reducing structural heterogeneity (Padule et al., 2022). Nevertheless, robust manufacturing under Good Manufacturing Practice (GMP) guidelines, coupled with stringent in-process controls, is indispensable to ensure inter-batch conformity (Peltonen, 2018).

For nano-enabled botanical or plant-metabolite products, regulatory readiness requires more than efficacy in animal models. Critical quality attributes should include particle size distribution, PDI, zeta potential, morphology, encapsulation efficiency, drug loading, residual solvent, sterility/endotoxin, in-use stability, release kinetics in biorelevant ocular media, and batch-to-batch consistency. For ophthalmic products, terminal sterilization may be incompatible with some nanocarriers; therefore aseptic processing or filtration must be validated without altering size, aggregation state, or release behavior (Wu C. et al., 2020; Wu et al., 2024).

Regulatory agencies like the EMA and FDA provide frameworks for nanomedicine QC, emphasizing maintenance of particle properties, stability, and bioavailability. However, specific guidelines for nanoformulations containing plant-derived metabolites remain underdeveloped, with existing regulations often tailored to traditional botanical preparations rather than advanced nanotechnological iterations (Arsude et al., 2023). Harmonized standards encompassing critical quality attributes (CQAs) are urgently needed. Key assays must include dynamic light scattering (DLS) for size/PDI, electrophoretic light scattering for zeta potential (critical for ocular retention and corneal penetration), chromatography for drug loading quantification, and biorelevant release kinetics testing under physiological conditions (e.g., simulated vitreous humor) (Gawin-Mikołajewicz et al., 2021). Traditional dialysis-based release methods often fail to predict *in vivo* behavior due to oversimplified conditions; emerging techniques like surface plasmon resonance or diffusion-ordered NMR spectroscopy offer more physiologically relevant insights into real-time drug release.

Looking ahead, artificial intelligence (AI) integrated with QbD paradigms accelerates optimization of formulation parameters and predicts release kinetics, enhancing QC efficiency (Alam, 2023). Concurrently, advanced characterization technologies—such as single-molecule detection and AI-driven data analytics—are poised to deepen understanding of nanoparticle behavior in biological matrices, improving batch consistency monitoring. International standardization efforts led by ISO (e.g., ISO/TS 4958 for liposomal drug quantification) and pharmacopeial bodies (e.g., USP <797> for sterile compounding) provide foundational guidelines, though adaptation for botanical nano-ophthalmics products is ongoing (Bhattacharjee, 2022). Ultimately, overcoming QC and standardization hurdles through interdisciplinary collaboration, innovative analytics, and adaptive regulation is paramount to unlocking the clinical potential of nanomaterial-delivered botanical-metabolite therapies for diabetic retinopathy.

### 6.2 Safety assessment

The translation of nanomaterial-enabled plant-derived bioactive metabolite delivery systems for diabetic retinopathy (DR) into clinical practice necessitates rigorous and multifaceted safety assessments, encompassing both the plant-derived bioactive metabolites and their nanomaterial carriers. This comprehensive evaluation is paramount to address potential acute and chronic toxicity, immunotoxicity, and genotoxicity, which could arise from the complex interactions between nanomaterials, plant-derived bioactive metabolites, and the unique ocular microenvironment. Current preclinical protocols emphasize a tiered approach: initial *in vitro* screening using corneal epithelial cell lines (e.g., human corneal epithelial cells) assesses acute cytotoxicity and irritation potential, often employing standardized tests like the Draize test on rabbit models for topical formulations to evaluate short-term ocular surface reactions such as redness, swelling, or epithelial damage (Rajan et al., 2024). For chronic toxicity, longitudinal *in vivo* studies in diabetic animal models (e.g., streptozotocin-induced rodents) are indispensable, monitoring not only systemic parameters like body weight, organ function (liver enzymes, renal biomarkers), and hematological profiles but also ocular-specific endpoints including intraocular pressure, electroretinogram (ERG) changes, and histological integrity of retinal layers over extended periods (Pei et al., 2024). These studies must account for the altered metabolic state in diabetes, which may influence drug metabolism and nanocarrier biodistribution, potentially exacerbating toxicity risks.

Immunotoxicity evaluation requires specialized assays due to the immune-privileged nature of the eye. Nanocarriers can inadvertently trigger inflammatory cascades or adaptive immune responses, particularly with repeated administration. Key assessments include measuring cytokine profiles (e.g., TNF-α, IL-6) in ocular tissues or vitreous fluid, flow cytometry analysis of immune cell infiltration (e.g., microglia activation in the retina), and hypersensitivity testing. For instance, dendrimers and liposomes have been documented to induce allergic reactions in ocular models, underscoring the need for carrier-specific immunogenic profiling. Genotoxicity remains a critical concern, especially given the potential for nanomaterials to induce oxidative DNA damage. Standard assays like the comet assay (single-cell gel electrophoresis) and micronucleus test should be performed on retinal cell lines *in vitro* and ocular tissues *in vivo*, with particular attention to nanoparticles’ ability to generate reactive oxygen species (ROS) – a known mechanism for retinal neuronal toxicity observed with metal nanoparticles like gold and silver (Scheive et al., 2021). The combined nano-enabled botanical-metabolite system adds complexity, as plant-derived bioactive metabolites (e.g., puerarin, berberine) may possess intrinsic genotoxic or immunomodulatory properties that could synergize or antagonize carrier effects, necessitating integrated testing rather than isolated assessments (Zhang et al., 2018).

Regulatory harmonization between agencies (FDA, EMA, WHO) presents both challenges and evolving frameworks for such multifaceted products. While the FDA’s “Final Guidances” on nanotechnology and EMA’s reflection papers provide foundational principles for nanomedicine characterization (size, surface charge, drug release kinetics), botanical therapeutics face additional scrutiny due to variability in active compound composition, pharmacokinetics, and potential botanical drug–drug interactions (Rodríguez et al., 2022). The EMA’s Committee on Herbal Medicinal Products (HMPC) and FDA’s Botanical Drug Development Guidance offer pathways but lack specific integration for nano-enabled botanical products (Zeng et al., 2023). Recent efforts, such as the EMA/FDA ATMP cluster and parallel scientific advice initiatives, aim to bridge gaps in evaluating “complex generics” or hybrid products, yet inconsistencies persist in requirements for demonstrating therapeutic equivalence, batch-to-batch reproducibility of nanocarriers, and long-term safety data (Iglesias-López et al., 2019; Hertig et al., 2021). Case studies like the clinical translation of liposomal cyclosporine (Verkazia®) for vernal keratoconjunctivitis illustrate the importance of robust chronic toxicity data, while failures in some polymeric micelle systems highlight the pitfalls of inadequate immunogenicity screening (Tewari et al., 2022).

To strengthen safety evaluation, several gaps remain to be addressed: 1) Developing standardized, biorelevant *in vitro* 3D retinal models (e.g., stem cell-derived “mini-retinas”) that better recapitulate the human blood-retinal barrier and neuronal microenvironment for chronic toxicity prediction (Chen et al., 2019); 2) Implementing multi-omics approaches (proteomics, metabolomics) in preclinical studies to identify novel toxicity biomarkers; 3) Establishing international registries for safety data for nano-enabled botanical-metabolite combinations to facilitate regulatory convergence (Musazzi et al., 2023). Only through such rigorous, integrated safety science can the promise of nanomaterial-enhanced botanical-metabolite therapies for DR be responsibly realized in clinical practice.

### 6.3 Regulatory frameworks

The regulatory landscape governing nanomaterial-enabled delivery of plant-derived bioactive metabolites for diabetic retinopathy (DR) therapy is characterized by evolving frameworks that struggle to reconcile the complexities of nanoformulations with traditional botanical medicine paradigms. Regulatory agencies like the U.S. FDA and European EMA have established guidelines for nanomedicines, emphasizing rigorous physicochemical characterization (e.g., particle size distribution, zeta potential, crystallinity via XRD) and *in vitro*/*in vivo* safety-efficacy profiling (Ramos et al., 2022). However, these guidelines—primarily designed for synthetic nanomaterials—face significant challenges when applied to nanoformulations containing plant-derived metabolites. Plant-derived bioactive metabolites (e.g., flavonoids, terpenoids) often exhibit variable composition and natural heterogeneity, complicating the standardization required for nanocarrier-based products (Xu et al., 2025). For instance, FDA’s 2014 guidance on nanotechnology products and EMA’s 2011 reflection paper outline quality control metrics but lack specificity for multi-component botanical nanosystems, where batch-to-batch consistency in active plant-derived metabolite loading remains problematic (Padule et al., 2022).

The discordance between regulatory frameworks is particularly acute in safety assessment. conventional botanical-medicine regulations, such as the EMA’s simplified registration for Chinese botanical medicines, assume predictable pharmacokinetics—an assumption invalidated by nano-encapsulation. Nanoformulations alter biodistribution, potentially enhancing retinal targeting but introducing novel toxicological profiles. Nanoparticles may accumulate in off-target organs (e.g., liver, spleen) or generate reactive oxygen species due to high surface-area reactivity, risks inadequately addressed by existing botanical-product safety protocols (Ambwani et al., 2018). Recent studies highlight oxidative stress and unintended immunogenicity from botanical nanoformulations, underscoring the need for nano-specific toxicology frameworks (D’Avenio et al., 2024). Compounding this, no standardized guidelines exist for systemic toxicological analysis of nano-enabled botanical medicines, leaving developers reliant on case-by-case evaluations that delay clinical translation (Jahangir, 2022; Zingg and Fischer, 2019).

Regulatory harmonization is therefore central to clinical translation. International initiatives like the REFINE project and EU-NCL (European Nanomedicine Characterization Laboratory) aim to standardize testing methods, yet gaps persist in defining “critical quality attributes” for nanoformulations containing plant-derived metabolites (Guo et al., 2014). Recent regulatory discussions and existing FDA/EMA guidance documents on nanotechnology-containing drug products, liposomal products, and complex nanomedicines highlight the need for case-by-case characterization of particle size, surface properties, release behavior, biodistribution, immunogenicity, sterility, and manufacturing reproducibility (Lv et al., 2024c). Moreover, intellectual property barriers arise when patenting nano-enhanced plant-derived metabolites, as regulatory agencies demand unprecedented characterization depth, conflicting with proprietary protection of traditional formulations (Zhu J. et al., 2024).

For diabetic retinopathy applications, regulatory pathways must adapt to nano-enabled advantages—like enhanced blood-retinal barrier penetration—while mitigating risks. Recent preclinical trials of resveratrol-loaded transferrin-modified liposomes (TF-RES-L) demonstrate improved neuroprotection in DR models but face scrutiny over long-term nanoparticle retention (Li et al., 2022). The absence of DR-specific guidelines for nano-enabled botanical products necessitates leveraging oncology precedents, such as nab-paclitaxel, where albumin nanoparticles improved solubility but required extensive safety re-evaluation (van Eerden et al., 2020; Sun et al., 2024). Future frameworks should incorporate real-time release monitoring and computational modeling to address dynamic interactions between plant-derived bioactive metabolites and nanocarriers, ensuring fidelity to both traditional efficacy and modern safety standards (Eder et al., 2022).

A translationally balanced regulatory discussion should also distinguish isolated purified metabolites from complex botanical drug substances. If a product contains a purified molecule, conventional small-molecule CMC and toxicology expectations may dominate; if it contains an extract or multi-component botanical preparation, botanical-source authentication, GACP, chemical profiling, and therapeutic consistency become central. For nano-enabled products, both layers must be integrated because nanoencapsulation can change biodistribution, exposure duration, immunogenicity, and ocular tissue retention (Wu et al., 2024).

In conclusion, bridging botanical medicine traditions with nanomedicine innovation demands agile, evidence-based regulations. Collaborative efforts among agencies, academia, and industry—guided by emerging data from clinical trials (e.g., NCT04809419 on curcumin nanoemulsions)—will be pivotal in unlocking the therapeutic potential of nano-enabled botanical products for diabetic retinopathy while safeguarding patient welfare (Hossen et al., 2019).

### 6.4 Bridging preclinical-clinical gap

The translation of nanomaterial-enabled plant-derived bioactive metabolite delivery systems for diabetic retinopathy (DR) from preclinical research to clinical application faces a significant challenge: the anatomical and physiological disparities between rodent models and humans. While rodents remain indispensable for initial efficacy screening due to their cost-effectiveness, genetic manipulability, and short reproductive cycles (Robinson et al., 2012), their utility in reliably predicting human ocular responses is fundamentally limited. Critically, rodents lack a macula—the central retinal region most severely affected in human DR pathologies like diabetic macular edema (DME). Their retinas are also avascular in the periphery (merangiotic pattern), contrasting sharply with the holangiotic vascularization of primates and humans (Wu et al., 2022). Consequently, rodent models consistently fail to develop advanced proliferative DR stages, including preretinal neovascularization and significant macular thickening, which are hallmarks of human disease progression. Additionally, their nocturnal nature, shorter lifespan, and inability to report visual function further restrict the translatability of safety and efficacy data (Kern et al., 2019).

This translational gap necessitates the strategic integration of higher mammalian models, particularly non-human primates (NHPs), in late-stage preclinical development. NHPs share over 95% genetic homology with humans and possess near-identical ocular anatomy, including a cone-rich macula, foveal pit, layered retinal vascular plexuses, and vitreous composition (Picaud et al., 2019). Recent studies leveraging advanced imaging techniques like optical coherence tomography angiography (OCTA) in macaques have demonstrated retinal microvascular changes under hyperglycemic conditions that closely mirror early human DR lesions—features impossible to replicate in rodents (Malek et al., 2018). Furthermore, the thicker internal limiting membrane (ILM) and larger vitreous volume in NHPs critically influence nanoparticle diffusion kinetics and retention times, making them indispensable for assessing the *in vivo* behavior of nanocarriers like polymeric nanoparticles or lipid-based systems intended for intravitreal delivery (Du et al., 2015; Gouin et al., 2022). Regulatory agencies increasingly recognize this; the FDA and EMA emphasize NHP data for complex ocular therapies where rodent-to-human extrapolation is unreliable, especially concerning biodistribution, long-term biocompatibility, and off-target effects of nanocarriers (Hines et al., 2020).

However, NHP studies present substantial ethical, logistical, and economic hurdles. Their use costs 10–20 times more than rodent studies, requires specialized facilities, and demands rigorous justification under the “3Rs” (Replacement, Reduction, Refinement) framework (Loiseau et al., 2023). To address this, a multidisciplinary approach is paramount: 1) Early rodent-NHP comparative studies should establish pharmacokinetic/pharmacodynamic (PK/PD) correlations using shared nanoformulations (e.g., nanoemulsions or dendrimers validated in both species) to refine dosing regimens (Rajan et al., 2024); 2) Advanced in vitro models (e.g., 3D retinal organoids with macula-like structures) and *in silico* simulations can partially replace NHPs for preliminary toxicity screening (Younis et al., 2022; de La Vega et al., 2024); and 3) Harmonized regulatory dialogues via EMA/FDA parallel scientific advice programs can align preclinical requirements, reducing redundant animal testing while ensuring robust safety datasets (Hines et al., 2020). Initiatives like the Nanotechnology Characterization Laboratory (NCL) exemplify successful academia-industry-regulatory partnerships, providing standardized protocols for nanocarrier characterization and bridging gaps in reproducibility (Tsai-hsuan Ku, 2012).

In conclusion, while rodent models retain value for mechanistic studies and initial nanoformulation screening, the anatomical fidelity of NHPs is irreplaceable for de-risking clinical translation of nanomedicines for DR. Strategic investment in NHP studies, coupled with innovative *in vitro* alternatives and cross-sector collaboration, is essential to overcome the preclinical-clinical gap and deliver effective, safe nanotherapies to patients. As emphasized by recent regulatory science strategies (EMA 2020–2025), such integrated approaches are not merely beneficial but imperative for accelerating the approval of complex ocular nanomedicines (Hines et al., 2020).

### 6.5 Clinical trial design considerations

The advancement of nanomaterial-enabled delivery systems for plant-derived bioactive metabolites in diabetic retinopathy (DR) necessitates rigorous regulatory frameworks and innovative clinical trial designs to ensure both safety and efficacy during translation. Current regulatory pathways for ocular nanomedicines must address unique challenges posed by the convergence of botanical metabolites and nanotechnology. Plant-derived bioactive metabolites often suffer from poor bioavailability, chemical instability, and potential toxicity at high doses—issues exacerbated by nanocarrier complexity (Kumar et al., 2022; Arifin et al., 2019). Regulatory agencies like the FDA and EMA lack harmonized guidelines for these hybrid products, leading to inconsistencies in quality control, safety assessment, and manufacturing standards (Bremer-Hoffmann et al., 2018). For instance, the EMA’s Committee on Herbal Medicinal Products and the FDA’s Nanotechnology Task Force have yet to establish unified protocols for characterizing nanoscale Chinese botanical medicine formulations, particularly concerning batch-to-batch variability and long-term biodistribution (Zeng et al., 2023). The FDA–EMA parallel scientific advice pilot program for complex generic/hybrid products may provide a useful regulatory dialogue model, although it was not designed specifically for botanical nanomedicines or nano-enabled plant-derived metabolite products (Halamoda-Kenzaoui et al., 2022). Moreover, intellectual property complexities arise from overlapping patents in nanotechnology and botanical medicine, further impeding commercialization.

In clinical trial design, defining appropriate endpoints for nano-enabled plant-derived bioactive metabolites requires balancing functional (patient-centered) and anatomical (objective biomarker-based) metrics. Functional endpoints, such as visual acuity and patient-reported outcome measures (PROMs), directly reflect therapeutic impact on quality of life. The National Eye Institute Visual Function Questionnaire (NEI-VFQ-25) and Patient-Acceptable Symptom Status (PASS) scales have validated sensitivity in DR trials, capturing nuances like reading ability and mobility (Jelin et al., 2019; Chakravarthy et al., 2019). However, their subjectivity necessitates complementary anatomical endpoints. Advances in imaging technologies now enable precise quantification of DR progression: ultra-wide-field fluorescein angiography can measure retinal capillary nonperfusion area—a surrogate marker strongly correlated with proliferative DR risk. Optical coherence tomography angiography (OCT-A) further provides high-resolution visualization of microvascular abnormalities, while adaptive optics scanning laser ophthalmoscopy (AO-SLO) tracks cellular-level changes in photoreceptors. Recent AI-integrated tools, such as the RETFound model (2023), enhance endpoint reliability by predicting DR progression from retinal images, potentially reducing trial durations (Vujosevic et al., 2024). Nevertheless, endpoint validation remains iterative; anatomical metrics must demonstrate consistent correlation with functional outcomes across diverse cohorts to gain regulatory acceptance as primary endpoints (Nair et al., 2016).

Preclinical studies from 2023 to 2025 underscore the efficacy of nanoformulated plant-derived bioactive metabolites in DR models, informing endpoint selection. For example, selenium nanoparticles (SeNPs) outperformed free selenium in streptozotocin-induced diabetic rats, restoring retinal thickness and downregulating VEGF and NF-κB expression. Functional recovery was evidenced by improved electroretinography readings, while anatomical improvements included reduced vascular leakage and glial activation (Ashraf Mahmoud Khashaba et al., 2025). Similarly, lutein-loaded nanoemulsions (Lutein-NEL) and baicalein nanodrops enhanced retinal antioxidant capacity and reduced GFAP overexpression, with effects quantifiable via both biochemical assays and histopathology (Ye et al., 2024). Chitosan-based nanocarriers improved sustained release of plant-derived bioactive metabolites like berberine, demonstrating 2.3-fold higher retinal bioavailability than oral administration in rabbit models—a metric measurable through HPLC analysis of vitreous samples. These studies advocate for composite endpoints in trials: functional metrics (e.g., contrast sensitivity) combined with anatomical biomarkers (e.g., macular thickness on OCT) and molecular indicators (e.g., aqueous humor VEGF levels) (Kąpa et al., 2025).

Realising this potential will require coordinated efforts across regulators, academia, and industry. QbD (Quality by Design) principles should be leveraged to standardize nanocarrier characterization (size, surface charge, drug release kinetics) (Halamoda-Kenzaoui et al., 2022). Clinical trials may also integrate AI-supported workflows for endpoint monitoring and patient stratification; for example, the B-PRODUCTIVE cluster-randomized trial reported approximately 40% higher specialist clinic productivity (1.59 vs. 1.14 completed encounters per hour) when an FDA-authorized autonomous AI system for diabetic eye exams was used compared with control (Abramoff et al., 2023). Concurrently, safety protocols must address nano-specific risks, including chronic inflammation from nanocarrier accumulation and botanical drug–drug interactions—requiring extended-phase IV surveillance post-approval (Yang and Merlin, 2023). The convergence of nanotechnology and botanical medicine holds transformative potential for DR management, yet its realization demands meticulous validation of clinically relevant endpoints and globally aligned regulatory rigor.

These considerations are integrated into the evidence-driven translation roadmap below, in which future studies should prioritize chemically defined materials, complete nanoformulation characterization, dose-response and time-course validation, DR-relevant retinal and *in vivo* models, appropriate blank-carrier and free-metabolite controls, quantitative ocular PK/biodistribution, and repeated-dose ocular and systemic safety assessment.

### 6.6 Evidence-driven translation roadmap

Future translation should move beyond platform novelty and be guided by evidence-driven product development. For ocular nanomedicines, regulatory success depends on reproducible GMP-compatible manufacturing, robust physicochemical characterization, sterile product presentation, predictable intraocular behavior, and standardized safety evaluation, because nanocarrier size, surface charge, drug loading, release kinetics, sterilization method, and ocular retention can directly affect biodistribution, efficacy, and toxicity (Iqbal et al., 2024; Coty and Vauthier, 2018). Therefore, future studies should define explicit go/no-go criteria before clinical advancement rather than relying solely on improved *in vitro* potency or formulation performance.

Combination delivery is a rational future direction because DR involves oxidative stress, inflammation, vascular leakage, neurodegeneration, and pathological angiogenesis. Co-encapsulation of plant-derived bioactive metabolites with established agents, such as anti-VEGF drugs, corticosteroids, or anti-inflammatory compounds, may provide broader pathway coverage than single-agent therapy (Kern et al., 2019). However, such combinations should be advanced only when free-metabolite, blank-carrier, single-agent, and combination controls demonstrate additive or synergistic benefit in DR-relevant models. Improved drug loading, cellular uptake, or cytokine suppression alone should not be interpreted as sufficient evidence of therapeutic superiority.

Hybrid and stimuli-responsive nanoplatforms also deserve further investigation. Nanoparticles embedded in electrospun nanofibers, hydrogel-integrated nanoparticles, lipid-polymer hybrid carriers, and stimuli-responsive systems triggered by pH, reactive oxygen species, enzymes, light, or magnetic fields may prolong ocular residence and enable spatiotemporally controlled release within diseased retinal microenvironments (Dludla et al., 2022). Nevertheless, these systems increase formulation complexity and regulatory burden. Future studies should therefore evaluate release reproducibility, trigger specificity, excipient safety, sterilization compatibility, and long-term ocular tolerance before claiming translational superiority.

Theranostic nanoplatforms provide another potential direction by integrating disease monitoring with controlled drug release. Wireless contact-lens-based systems for intraocular pressure sensing and triggered drug delivery illustrate how diagnostic and therapeutic functions can be combined in ocular applications (Yang et al., 2022). For DR, similar concepts could be adapted cautiously toward biomarker-responsive monitoring or localized release systems, but such applications remain conceptual unless supported by validated DR biomarkers, quantitative retinal exposure data, and functional efficacy endpoints.

**FIGURE 4:**
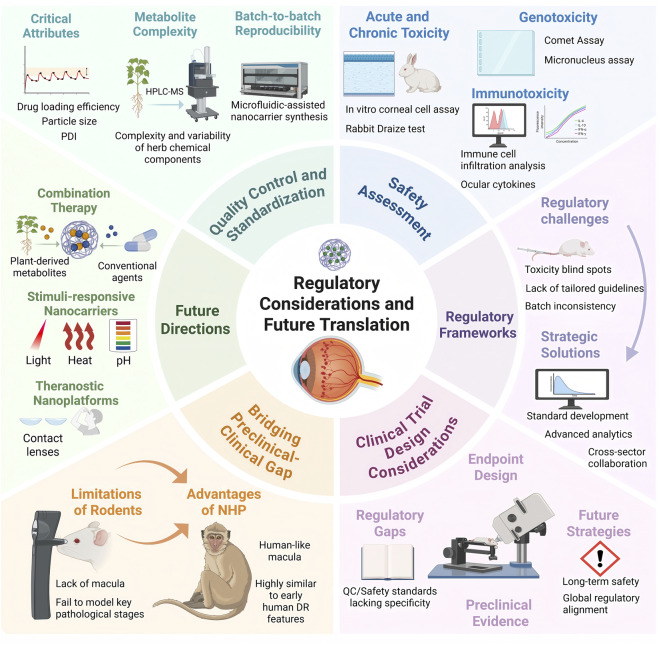
Translational roadmap for clinical implementation of nano-enabled plant-derived metabolite therapeutics. This schematic presents an integrated translational roadmap that organises the six interrelated domains governing the clinical implementation of nano-enabled plant-derived bioactive metabolite therapeutics for diabetic retinopathy (DR), corresponding to the structure of Section 6. (i) Quality control and standardisation addresses critical quality attributes such as drug loading, particle size, and polydispersity index (PDI); HPLC- and mass spectrometry-based authentication of metabolite identity, purity, and chemical complexity; and batch-to-batch reproducibility, with microfluidic-assisted nanocarrier synthesis highlighted as a representative scalable manufacturing approach. (ii) Safety assessment integrates acute and chronic toxicity evaluation (*in vitro* corneal cell assays and the Draize test), genotoxicity testing (comet and micronucleus assays), and immunotoxicity profiling (immune-cell infiltration analysis and ocular cytokine measurement). (iii) Regulatory frameworks identify current regulatory challenges—including toxicity blind spots, lack of tailored guidance for nano-enabled botanical products, and batch inconsistency—alongside strategic solutions such as standards development, advanced analytics, and cross-sector collaboration. (iv) Clinical trial design considerations encompass endpoint selection, regulatory gaps in QC and safety specifications, the use of preclinical evidence to inform trial design, and future strategies covering long-term safety monitoring and global regulatory alignment. (v) Bridging the preclinical-clinical gap acknowledges the inherent limitations of rodent DR models (absence of a macula and failure to model advanced disease stages) and the complementary value of non-human primate (NHP) models, which provide human-like macular anatomy and reproduce early human DR features. (vi) Future directions highlight combination therapies that pair plant-derived metabolites with conventional ophthalmic agents, stimuli-responsive nanocarriers triggered by light, heat, pH, reactive oxygen species, or enzymes, and theranostic nanoplatforms exemplified by wireless contact-lens-based systems. Collectively, these six domains define an evidence-driven framework for advancing candidate nanoformulations from preclinical proof-of-concept to clinically viable DR therapeutics. Abbreviations: DR, diabetic retinopathy; HPLC, high-performance liquid chromatography; MS, mass spectrometry; NHP, non-human primate; PDI, polydispersity index; QC, quality control; ROS, reactive oxygen species.

Finally, manufacturability should be considered early rather than after preclinical efficacy has been demonstrated. Complex nanomaterials require scalable and reproducible production methods, such as microfluidics, high-pressure homogenization, or microfluidizer-based high-shear processing, but scale-up may alter colloidal stability, particle-size distribution, encapsulation efficiency, and release kinetics (Kenneth and Thomai, 2010). The most realistic translational path is therefore not to nominate a universally superior carrier, but to match each metabolite with a delivery system according to its solubility, stability, required retinal exposure, intended route of administration, safety profile, and manufacturing feasibility (Figure 4).

## 7 Conclusion

Diabetic retinopathy remains a difficult therapeutic target because its progression involves vascular leakage, inflammation, oxidative stress, neurodegeneration, blood-retinal barrier dysfunction, and pathological angiogenesis. Plant-derived bioactive metabolites provide attractive multi-target pharmacological potential, but their translation is still limited by poor solubility, rapid metabolism, low and variable systemic exposure, insufficient posterior-segment delivery, and uneven preclinical evidence quality. Therefore, their therapeutic value should not be judged only by improved solubility, cellular uptake, or isolated *in vitro* pathway modulation.

Nanomaterial-enabled delivery systems offer a rational strategy to address these limitations by improving metabolite stability, ocular residence, controlled release, and potentially retinal exposure. However, no single nanocarrier platform is universally optimal. Polymeric nanoparticles, lipid-based carriers, exosomes, dendrimers, hydrogels, and stimuli-responsive systems each have distinct advantages and limitations in drug loading, release behavior, barrier penetration, safety, manufacturability, and regulatory complexity. The most defensible translational strategy is therefore to match each metabolite with an appropriate delivery platform according to its physicochemical properties, intended route of administration, required retinal exposure, safety profile, and scale-up feasibility.

Future studies should move beyond proof-of-concept formulation improvement toward rigorous translational validation. Priority should be given to standardized purified-metabolite interventions, well-characterized nanocarriers, free-drug and blank-carrier controls, dose-response assessment, quantitative ocular pharmacokinetics, retina-to-plasma exposure analysis, repeated-dose ocular safety, and disease-relevant functional endpoints in STZ, OIR, or other DR-relevant models. Advanced *in vitro* barrier systems, *ex vivo* screening, and *in vivo* imaging can help prioritize candidates, but clinical promise should ultimately depend on reproducible efficacy, retinal exposure, long-term tolerability, and manufacturability. Overall, nanomaterial-based delivery may help convert plant-derived bioactive metabolites from pharmacologically promising compounds into credible candidates for DR therapy, but this transition requires evidence-driven platform selection and stricter preclinical-to-clinical translation standards.

## References

[B1] Abramoff M. D. Whitestone N. Patnaik J. L. Rich E. Ahmed M. Husain L. (2023). Autonomous artificial intelligence increases real-world specialist clinic productivity in a cluster-randomized trial. NPJ Digit. Med. 6 (1), 184. 10.1038/s41746-023-00931-7 37794054 PMC10550906

[B2] Alam M. K. (2023). Nanocarrier-based drug delivery systems using microfluidic-assisted techniques. Adv. Nanobiomed Res. 3 (11), 2300041. 10.1002/anbr.202300041

[B3] Alkholief M. Albasit H. Alhowyan A. Alshehri S. Raish M. Abul Kalam M. (2019). Employing a PLGA-TPGS based nanoparticle to improve the ocular delivery of Acyclovir. Saudi Pharm. J. 27 (2), 293–302. 10.1016/j.jsps.2018.11.011 30766442 PMC6362158

[B4] Alom M. Z. Kapur R. P. Bowen T. Asari V. K. (2021). GanglionNet: objectively assess the density and distribution of ganglion cells with NABLA-N network. Inf. Med. Unlocked 23, 100518. 10.1016/j.imu.2021.100518

[B5] Amadio M. Bucolo C. Leggio G. M. Drago F. Govoni S. Pascale A. (2010). The PKCbeta/HuR/VEGF pathway in diabetic retinopathy. Biochem. Pharmacol. 80 (8), 1230–1237. 10.1016/j.bcp.2010.06.033 20599775

[B6] Ambwani S. Tandon R. Ambwani T. K. Malik Y. S. (2018). Current knowledge on nanodelivery systems and their beneficial applications in enhancing the efficacy of herbal drugs. J. Exp. Biol. Agric. Sci. 6, 87–107. 10.18006/2018.6(1).87.107

[B7] Anukunwithaya T. Poo P. Hunsakunachai N. Rodsiri R. Malaivijitnond S. Khemawoot P. (2018). Absolute oral bioavailability and disposition kinetics of puerarin in female rats. BMC Pharmacol. Toxicol. 19 (1), 25. 10.1186/s40360-018-0216-3 29801513 PMC5970530

[B8] Arifin S. AlShami A. Omar S. S. S. Jalil A. A. Khalid K. A. Hadi H. (2019). Impact of modern technology on the development of natural-based products. J. Ayurvedic Herb. Med. 5, 133–142. 10.31254/jahm.2019.5404

[B9] Arsude J. Chavan M. Joshi S. Pethakar S. Dama G. (2023). Herbal nano formulations for topical drug delivery: prospective for multiple skin disorders. Int. J. Drug Deliv. Technol. 13, 1568–1577. 10.25258/ijddt.13.4.69

[B10] Ashraf Mahmoud Khashaba N. Abdel Hafez S. M. N. Abdelzaher W. Y. Ahmed Rifaai R. Abdel Majeed N. A. M. (2025). Therapeutic effects of selenium nanoparticles versus selenium on experimentally induced diabetic retinopathy via modulation of TLR4/NF-kB P65/VEGF/connexin 43 signaling. Tissue Barriers 13, 2491911. 10.1080/21688370.2025.2491911 40247438 PMC12667640

[B11] Bapputty R. Talahalli R. Zarini S. Samuels I. Murphy R. Gubitosi-Klug R. (2019). Montelukast prevents early diabetic retinopathy in mice. Diabetes 68 (10), 2004–2015. 10.2337/db19-0026 31350303 PMC6754245

[B12] Barzaghi N. Crema F. Gatti G. Pifferi G. Perucca E. (1990). Pharmacokinetic studies on IdB 1016, a silybin-phosphatidylcholine complex, in healthy human subjects. Eur. J. Drug Metab. Pharmacokinet. 15 (4), 333–338. 10.1007/BF03190223 2088770

[B13] Beirampour N. Bustos-Salgado P. Garrós N. Mohammadi-Meyabadi R. Domènech Ò. Suñer-Carbó J. (2024). Formulation of polymeric nanoparticles loading baricitinib as a topical approach in ocular application. Pharmaceutics 16 (8), 1092. 10.3390/pharmaceutics16081092 39204436 PMC11360485

[B14] Bhattacharjee S. (2022). Craft of Co-encapsulation in nanomedicine: a struggle to achieve synergy through reciprocity. ACS Pharmacol. Transl. Sci. 5 (5), 278–298. 10.1021/acsptsci.2c00033 35592431 PMC9112416

[B15] Bidwai P. Gite S. Pahuja K. Kotecha K. (2022). A systematic literature review on diabetic retinopathy using an artificial intelligence approach. Big Data Cognitive Comput. 6, 152. 10.3390/bdcc6040152

[B16] Blass S. (2010). Permeability studies on the ocular absorbance of nanostructured materials across the cornea. Sci. Pharm. 78, 678. 10.3797/scipharm.cespt.8.pnm08

[B17] Bolz S. N. Adasme M. F. Schroeder M. (2021). Toward an understanding of pan-assay interference compounds and promiscuity: a structural perspective on binding modes. J. Chem. Inf. Model 61 (5), 2248–2262. 10.1021/acs.jcim.0c01227 33899463

[B18] Bora K. Kushwah N. Maurya M. Pavlovich M. C. Wang Z. Chen J. (2023). Assessment of inner blood-retinal barrier: animal models and methods. Cells 12 (20), 2443. 10.3390/cells12202443 37887287 PMC10605292

[B19] Bradley L. J. Ward A. Hsue M. C. Y. Liu J. Copland D. A. Dick A. D. (2021). Quantitative assessment of experimental ocular inflammatory disease. Front. Immunol. 12, 630022. 10.3389/fimmu.2021.630022 34220797 PMC8250853

[B20] Bremer-Hoffmann S. Halamoda-Kenzaoui B. Borgos S. E. F. (2018). Identification of regulatory needs for nanomedicines. J. Interdiscip. Nanomed. 3 (1), 4–15. 10.1002/jin2.34

[B21] Cammalleri M. Filippi L. Dal Monte M. Bagnoli P. (2024). A promising case of preclinical-clinical translation: β-adrenoceptor blockade from the oxygen-induced retinopathy model to retinopathy of prematurity. Front. Physiol. 15, 1408605. 10.3389/fphys.2024.1408605 38938747 PMC11208707

[B22] Caridi B. Doncheva D. Sivaprasad S. Turowski P. (2021). Galectins in the pathogenesis of common retinal disease. Front. Pharmacol. 12, 687495. 10.3389/fphar.2021.687495 34079467 PMC8165321

[B23] Dei Cas M. Ghidoni R. (2019). Dietary curcumin: correlation between bioavailability and health potential. Nutrients 11 (9), 2147. 10.3390/nu11092147 31500361 PMC6770259

[B24] Chai G. R. Liu S. Yang H.-W. Chen X.-L. (2021). Quercetin protects against diabetic retinopathy in rats by inducing heme oxygenase-1 expression. Neural Regen. Res. 16 (7), 1344–1350. 10.4103/1673-5374.301027 33318415 PMC8284280

[B25] Chakravarthy U. Pearce I. Banerjee S. Burton B. J. L. Downey L. Gale R. (2019). Patient-reported outcomes in the RELIGHT clinical trial of ranibizumab in diabetic macular oedema. BMJ Open Ophthalmol. 4 (1), e000226. 10.1136/bmjophth-2018-000226 31179389 PMC6528749

[B26] Chanphai P. Tajmir-Riahi H. A. (2018). Binding analysis of antioxidant polyphenols with PAMAM nanoparticles. J. Biomol. Struct. Dyn. 36 (13), 3487–3495. 10.1080/07391102.2017.1391124 29019428

[B27] Chao H. M. Chuang M. J. Liu J. H. Liu X. Q. Ho L. K. Pan W. H. T. (2013). Baicalein protects against retinal ischemia by antioxidation, antiapoptosis, downregulation of HIF-1α, VEGF, and MMP-9 and upregulation of HO-1. J. Ocul. Pharmacol. Ther. 29 (6), 539–549. 10.1089/jop.2012.0179 23537149 PMC3708628

[B28] Chen W. Miao Y. Q. Fan D. J. Yang S. S. Lin X. Meng L. K. (2011). Bioavailability study of berberine and the enhancing effects of TPGS on intestinal absorption in rats. AAPS PharmSciTech 12 (2), 705–711. 10.1208/s12249-011-9632-z 21637946 PMC3134654

[B29] Chen Y. Sawada O. Kohno H. Le Y. Z. Subauste C. Maeda T. (2013). Autophagy protects the retina from light-induced degeneration. J. Biol. Chem. 288 (11), 7506–7518. 10.1074/jbc.M112.439935 23341467 PMC3597791

[B30] Chen D. W. Narsineni L. Foldvari M. (2019). Multipotent stem cell-derived retinal ganglion cells in 3D culture as tools for neurotrophic factor gene delivery system development. Nanomedicine 21, 102045. 10.1016/j.nano.2019.102045 31255791

[B31] Chen N. Li Y. Huang N. Yao J. Luo W. F. Jiang Q. (2020). The Nrf2 activator MIND4-17 protects retinal ganglion cells from high glucose-induced oxidative injury. J. Cell Physiol. 235 (10), 7204–7213. 10.1002/jcp.29619 32020639

[B32] Chen Q. Yang Z. Liu H. Man J. Oladejo A. O. Ibrahim S. (2024). Novel drug delivery systems: an important direction for drug innovation research and development. Pharmaceutics 16 (5), 674. 10.3390/pharmaceutics16050674 38794336 PMC11124876

[B33] Chen X. Zhang W. Xu P. Zhao Z. Zheng Y. Shi D. (2024). FFA-GPT: an automated pipeline for fundus fluorescein angiography interpretation and question-answer. NPJ Digit. Med. 7 (1), 111. 10.1038/s41746-024-01101-z 38702471 PMC11068733

[B34] Cheng H. Chawla A. Yang Y. Li Y. Zhang J. Jang H. L. (2017). Development of nanomaterials for bone-targeted drug delivery. Drug Discov. Today 22 (9), 1336–1350. 10.1016/j.drudis.2017.04.021 28487069 PMC5644493

[B35] Cheng S. C. Huang W. C. S Pang J. H. Wu Y. H. Cheng C. Y. (2019). Quercetin inhibits the production of IL-1β-Induced inflammatory cytokines and chemokines in ARPE-19 cells via the MAPK and NF-κB signaling pathways. Int. J. Mol. Sci. 20 (12), 2957. 10.3390/ijms20122957 31212975 PMC6628093

[B36] Cheung A. K. L. Wong C. H. L. Ho L. Wu I. X. Y. Ke F. Y. T. Chung V. C. H. (2022). Methodological quality of systematic reviews on Chinese herbal medicine: a methodological survey. BMC Complement. Med. Ther. 22 (1), 48. 10.1186/s12906-022-03529-w 35197038 PMC8867833

[B37] Cimino C. Zingale E. Bonaccorso A. Musumeci T. Carbone C. Pignatello R. (2024). From preformulative design to *in vivo* tests: a complex path of requisites and studies for nanoparticle ocular application. Part 1: design, characterization, and preliminary *in vitro* studies. Mol. Pharm. 21 (12), 6034–6061. 10.1021/acs.molpharmaceut.4c00554 39441703

[B38] Di Costanzo A. Angelico R. (2019). Formulation strategies for enhancing the bioavailability of silymarin: the state of the art. Molecules 24 (11), 2155. 10.3390/molecules24112155 31181687 PMC6600503

[B39] Coty J. B. Vauthier C. (2018). Characterization of nanomedicines: a reflection on a field under construction needed for clinical translation success. J. Control Release 275, 254–268. 10.1016/j.jconrel.2018.02.013 29454063

[B40] D’Avenio G. Daniele C. Grigioni M. (2024). Nanostructured medical devices: regulatory perspective and current applications. Mater. (Basel) 17 (8), 1787. 10.3390/ma17081787 PMC11051465 38673144

[B41] Ding R. Hase Y. Ameen-Ali K. E. Ndung'u M. Stevenson W. Barsby J. (2020). Loss of capillary pericytes and the blood-brain barrier in white matter in poststroke and vascular dementias and Alzheimer’s disease. Brain Pathol. 30 (6), 1087–1101. 10.1111/bpa.12888 32705757 PMC8018063

[B42] Dludla S. B. K. Mashabela L. T. Ng'andwe B. Makoni P. A. Witika B. A. (2022). Current advances in nano-based and polymeric stimuli-responsive drug delivery targeting the ocular microenvironment: a review and envisaged future perspectives. Polym. (Basel) 14 (17), 3580. 10.3390/polym14173580 PMC9460529 36080651

[B43] Dong G. M. Yu H. Pan L. B. Ma S. R. Xu H. Zhang Z. W. (2021). Biotransformation of timosaponin BII into seven characteristic metabolites by the gut microbiota. Molecules 26 (13), 3861. 10.3390/molecules26133861 34202717 PMC8270264

[B44] Du W. Tao Y. Deng W. T. Zhu P. Li J. Dai X. (2015). Vitreal delivery of AAV vectored Cnga3 restores cone function in CNGA3-/-/Nrl-/- mice, an all-cone model of CNGA3 achromatopsia. Hum. Mol. Genet. 24 (13), 3699–3707. 10.1093/hmg/ddv114 25855802 PMC4459390

[B230] Duan H.‐H. Huang J.‐M. Yu S.‐Y. Li W. Qiao Y. Tang M.‐K. (2013). Vitreal delivery of AAV vectored Cnga3 restores cone function in CNGA3-/-/Nrl-/- mice, an all-cone model of CNGA3 achromatopsia. Hum. Mol. Genet. 24 (13), 3699–3707. 10.1093/hmg/ddv114 PMC4459390 25855802

[B45] Durak S. Esmaeili Rad M. Alp Yetisgin A. Eda Sutova H. Kutlu O. Cetinel S. (2020). Niosomal drug delivery systems for ocular disease-recent advances and future prospects. Nanomater. (Basel) 10 (6), 1191. 10.3390/nano10061191 32570885 PMC7353242

[B46] Dürr D. Stieger B. Kullak-Ublick G. A. Rentsch K. M. Steinert H. C. Meier P. J. (2000). St John's Wort induces intestinal P-glycoprotein/MDR1 and intestinal and hepatic CYP3A4. Clin. Pharmacol. Ther. 68 (6), 598–604. 10.1067/mcp.2000.112240 11180019

[B47] Eder K. M. Marzi A. Wågbø A. M. Vermeulen J. P. de la Fonteyne-Blankestijn L. J. J. Rösslein M. (2022). Standardization of an *in vitro* assay matrix to assess cytotoxicity of organic nanocarriers: a pilot interlaboratory comparison. Drug Deliv. Transl. Res. 12 (9), 2187–2206. 10.1007/s13346-022-01203-9 35794354 PMC9360155

[B48] van Eerden R. A. G. Mathijssen R. H. J. Koolen S. L. W. (2020). Recent clinical developments of nanomediated drug delivery systems of taxanes for the treatment of cancer. Int. J. Nanomedicine 15, 8151–8166. 10.2147/IJN.S272529 33132699 PMC7592152

[B49] Fosse V. Oldoni E. Bietrix F. Budillon A. Daskalopoulos E. P. Fratelli M. (2023). Recommendations for robust and reproducible preclinical research in personalised medicine. BMC Med. 21 (1), 14. 10.1186/s12916-022-02719-0 36617553 PMC9826728

[B50] Gabai A. Zeppieri M. Finocchio L. Salati C. (2023). Innovative strategies for drug delivery to the ocular posterior segment. Pharmaceutics 15 (7), 1862. 10.3390/pharmaceutics15071862 37514050 PMC10385847

[B51] Ganapathy D. Shanmugam R. Sekar D. (2020). Current status of nanoparticles loaded medication in the management of diabetic retinopathy. J. Evol. Med. Dent. Sci. 9, 1713–1718. 10.14260/jemds/2020/376

[B52] Gaudana R. Ananthula H. K. Parenky A. Mitra A. K. (2010). Ocular drug delivery. Aaps J. 12 (3), 348–360. 10.1208/s12248-010-9183-3 20437123 PMC2895432

[B53] Gawin-Mikołajewicz A. Nartowski K. P. Dyba A. J. Gołkowska A. M. Malec K. Karolewicz B. (2021). Ophthalmic nanoemulsions: from composition to technological processes and quality control. Mol. Pharm. 18 (10), 3719–3740. 10.1021/acs.molpharmaceut.1c00650 34533317 PMC8493553

[B54] Giap B. D. Likosky K. Srinivasan K. Khan N. W. Nallasamy N. (2024). Automated abnormality detection in patient retinal function: a deep learning-powered electroretinogram analysis system. Annu. Int. Conf. IEEE Eng. Med. Biol. Soc. 2024, 1–4. 10.1109/EMBC53108.2024.10782233 40039563

[B55] Gouin T. Ellis-Hutchings R. Thornton Hampton L. M. Lemieux C. L. Wright S. L. (2022). Screening and prioritization of nano- and microplastic particle toxicity studies for evaluating human health risks - development and application of a toxicity study assessment tool. Microplast Nanoplast 2 (1), 2. 10.1186/s43591-021-00023-x 35098152 PMC8760192

[B56] Guo J. W. Lee Y. H. Huang H. W. Tzou M. C. Wang Y. J. Tsai J. C. (2014). Development of Taiwan's strategies for regulating nanotechnology-based pharmaceuticals harmonized with international considerations. Int. J. Nanomed. 9, 4773–4783. 10.2147/IJN.S68134 25342901 PMC4206375

[B57] Gupta B. Mishra V. Gharat S. Momin M. Omri A. (2021). Cellulosic polymers for enhancing drug bioavailability in ocular drug delivery systems. Pharm. (Basel) 14 (11), 1201. 10.3390/ph14111201 PMC8621906 34832983

[B58] Halamoda-Kenzaoui B. Geertsma R. Pouw J. Prina-Mello A. Carrer M. Roesslein M. (2022). Future perspectives for advancing regulatory science of nanotechnology-enabled health products. Drug Deliv. Transl. Res. 12 (9), 2145–2156. 10.1007/s13346-022-01165-y 35691982 PMC9360093

[B59] Han M. Sha X. Wu Y. Fang X. (2006). Oral absorption of ginsenoside Rb1 using *in vitro* and *in vivo* models. Planta Med. 72 (5), 398–404. 10.1055/s-2005-916211 16557452

[B60] He X. Fang L. Chen X. Zhang Z. Zhou S. Qi D. (2025). Application of a 3D human cornea-like epithelium for eye irritation assessment of contact lens solutions. J. Appl. Toxicol. 45 (10), 1984–1994. 10.1002/jat.4820 40425172

[B61] Heinrich M. Jalil B. Abdel-Tawab M. Echeverria J. Kulić Ž. McGaw L. J. (2022). Best practice in the chemical characterisation of extracts used in pharmacological and toxicological research-The ConPhyMP-Guidelines. Front. Pharmacol. 13, 953205. 10.3389/fphar.2022.953205 36176427 PMC9514875

[B62] Hertig J. B. Shah V. P. Flühmann B. Mühlebach S. Stemer G. Surugue J. (2021). Tackling the challenges of nanomedicines: are we ready? Am. J. Health Syst. Pharm. 78 (12), 1047–1056. 10.1093/ajhp/zxab048 33599767 PMC7929390

[B63] Hines P. A. Gonzalez-Quevedo R. Lambert A. I. O. M Janssens R. Freischem B. Edo J. T. (2020). Regulatory science to 2025: an analysis of stakeholder responses to the european medicines agency’s strategy. Front Med (Lausanne) 7, 508. 10.3389/fmed.2020.00508 33072771 PMC7540226

[B64] Hollanders K. Hove I. V. Sergeys J. Bergen T. V. Lefevere E. Kindt N. (2017). AMA0428, A potent rock inhibitor, attenuates early and late experimental diabetic retinopathy. Curr. Eye Res. 42 (2), 260–272. 10.1080/02713683.2016.1183030 27399806

[B65] Hossen S. Hossain M. K. Basher M. K. Mia M. N. H. Rahman M. T. Uddin M. J. (2019). Smart nanocarrier-based drug delivery systems for cancer therapy and toxicity studies: a review. J. Adv. Res. 15, 1–18. 10.1016/j.jare.2018.06.005 30581608 PMC6300464

[B66] Hu J. Zhang J. Zhao W. Zhang Y. Zhang L. Shang H. (2011). Cochrane systematic reviews of Chinese herbal medicines: an overview. PLoS One 6 (12), e28696. 10.1371/journal.pone.0028696 22174870 PMC3235143

[B67] Hu Z. Wang X. Hu Q. Chen X. (2023). Exploring the protective effects of herbal monomers against diabetic retinopathy based on the regulation of autophagy and apoptosis: a review. Med. Baltim. 102 (43), e35541. 10.1097/md.0000000000035541 PMC10615407 37904448

[B68] Iglesias-López C. Agustí A. Obach M. Vallano A. (2019). Regulatory framework for advanced therapy medicinal products in Europe and United States. Front. Pharmacol. 10, 921. 10.3389/fphar.2019.00921 31543814 PMC6728416

[B69] Imperiale J. C. Acosta G. B. Sosnik A. (2018). Polymer-based carriers for ophthalmic drug delivery. J. Control Release 285, 106–141. 10.1016/j.jconrel.2018.06.031 29964135

[B70] Ioannides C. (2002). Pharmacokinetic interactions between herbal remedies and medicinal drugs. Xenobiotica 32 (6), 451–478. 10.1080/00498250210124147 12160480

[B71] Iordache T. A. Badea N. Mihaila M. Crisan S. Pop A. L. Lacatusu I. (2021). Challenges in coopted hydrophilic and lipophilic herbal bioactives in the same nanostructured carriers for effective bioavailability and anti-inflammatory action. Nanomater. (Basel) 11 (11), 3035. 10.3390/nano11113035 PMC8624441 34835798

[B72] Iqbal H. Razzaq A. Zhou D. Lou J. Xiao R. Lin F. (2024). Nanomedicine in glaucoma treatment; current challenges and future perspectives. Mater Today Bio 28, 101229. 10.1016/j.mtbio.2024.101229 PMC11409099 39296355

[B73] Jacob S. Nair A. B. Shah J. Gupta S. Boddu S. H. S. Sreeharsha N. (2022). Lipid nanoparticles as a promising drug delivery carrier for topical ocular Therapy-An overview on recent advances. Pharmaceutics 14 (3), 533. 10.3390/pharmaceutics14030533 35335909 PMC8955373

[B74] Jahangir M. (2022). Nanophytomedicine in clinical management: an introductory evidence-based review. J. Pharm. Res. Sci. and Technol. 6, 26–37. 10.31531/jprst.1000158

[B75] Jalili A. Bagherifar R. Nokhodchi A. Conway B. Javadzadeh Y. (2023). Current advances in nanotechnology-mediated delivery of herbal and plant-derived medicines. Adv. Pharm. Bull. 13 (4), 712–722. 10.34172/apb.2023.087 38022806 PMC10676547

[B76] Jarmila P. Veronika M. Peter M. (2024). Advances in the delivery of anticancer drugs by nanoparticles and chitosan-based nanoparticles. Int. J. Pharm. 8, 100281. 10.1016/j.ijpx.2024.100281 PMC11408389 39297017

[B77] Jelin E. Wisløff T. Jørstad Ø. K. Heiberg T. Moe M. C. (2019). Patient-reported outcome measures in the management of neovascular age-related macular degeneration: a 1-year prospective study. BMJ Open Ophthalmol. 4 (1), e000353. 10.1136/bmjophth-2019-000353 31673632 PMC6797267

[B78] Jin L. Li X. Chen X. Chen X. Liu Y. Xu H. (2023). A study on puerarin *in situ* gel eye drops: formulation optimization and pharmacokinetics on rabbits by microdialysis. Int. J. Pharm. 642, 123176. 10.1016/j.ijpharm.2023.123176 37364779

[B79] Joussen A. M. Doehmen S. Le M. L. Koizumi K. Radetzky S. Krohne T. U. (2009). TNF-alpha mediated apoptosis plays an important role in the development of early diabetic retinopathy and long-term histopathological alterations. Mol. Vis. 15, 1418–1428. 19641635 PMC2716944

[B80] Kannan R. M. Pitha I. Parikh K. S. (2023). A new era in posterior segment ocular drug delivery: translation of systemic, cell-targeted, dendrimer-based therapies. Adv. Drug Deliv. Rev. 200, 115005. 10.1016/j.addr.2023.115005 37419213

[B81] Kąpa M. Koryciarz I. Kustosik N. Jurowski P. Pniakowska Z. (2025). Future directions in diabetic retinopathy treatment: stem cell therapy, nanotechnology, and PPARα modulation. J. Clin. Med. 14 (3), 683. 10.3390/jcm14030683 39941353 PMC11818668

[B82] Kaštelan S. Konjevoda S. Sarić A. Urlić I. Lovrić I. Čanović S. (2025). Resveratrol as a novel therapeutic approach for diabetic retinopathy: molecular mechanisms, clinical potential, and future challenges. Molecules 30 (15), 3262. 10.3390/molecules30153262 40807437 PMC12348093

[B83] Kenneth J. C. Thomai P. P. D. (2010). Large scale nanomaterial production using microfluidizer high shear processing. MRS Online Proc. Libr. 1209 (1), 301. 10.1557/PROC-1209-P03-01

[B84] Kern T. S. Antonetti D. A. Smith L. E. H. (2019). Pathophysiology of diabetic retinopathy: contribution and limitations of laboratory research. Ophthalmic Res. 62 (4), 196–202. 10.1159/000500026 31362288 PMC6872907

[B85] Kesharwani P. Iyer A. K. (2015). Recent advances in dendrimer-based nanovectors for tumor-targeted drug and gene delivery. Drug Discov. Today 20 (5), 536–547. 10.1016/j.drudis.2014.12.012 25555748 PMC4433832

[B86] Khanal S. Adhikari U. Rijal N. P. Bhattarai S. R. Sankar J. Bhattarai N. (2016). pH-Responsive PLGA nanoparticle for controlled payload delivery of diclofenac sodium. J. Funct. Biomater. 7 (3), 21. 10.3390/jfb7030021 27490577 PMC5040994

[B87] Kim J. Kim K. M. Kim C. S. Sohn E. Lee Y. M. Jo K. (2012). Puerarin inhibits the retinal pericyte apoptosis induced by advanced glycation end products *in vitro* and *in vivo* by inhibiting NADPH oxidase-related oxidative stress. Free Radic. Biol. Med. 53 (2), 357–365. 10.1016/j.freeradbiomed.2012.04.030 22609359

[B88] Klotzsche-von Ameln A. Sprott D. (2022). Harnessing retinal phagocytes to combat pathological neovascularization in ischemic retinopathies? Pflugers Arch. 474 (6), 575–590. 10.1007/s00424-022-02695-7 35524802 PMC9117346

[B89] Kocak M. Ezazi Erdi S. Jorba G. Maestro I. Farrés J. Kirkin V. (2022). Targeting autophagy in disease: established and new strategies. Autophagy 18 (3), 473–495. 10.1080/15548627.2021.1936359 34241570 PMC9037468

[B90] Krishnaiah Y. (2010). Pharmaceutical technologies for enhancing oral bioavailability of poorly soluble drugs. J. Bioequivalence and Bioavailab. 2 (2), 28‐36. 10.4172/jbb.1000027

[B91] Kumar B. Gupta S. K. Nag T. C. Srivastava S. Saxena R. Jha K. A. (2014). Retinal neuroprotective effects of quercetin in streptozotocin-induced diabetic rats. Exp. Eye Res. 125, 193–202. 10.1016/j.exer.2014.06.009 24952278

[B92] Kumar R. Mirza M. A. Naseef P. P. Kuruniyan M. S. Zakir F. Aggarwal G. (2022). Exploring the potential of natural product-based nanomedicine for maintaining oral health. Molecules 27 (5), 1725. 10.3390/molecules27051725 35268826 PMC8911592

[B93] Kwon K. Hwang Y. Jung J. Tae G. (2021). Enhanced transport and permeation of a polymeric nanocarrier across the retina by mixing with ATP upon intravitreal injection. Pharmaceutics 13 (4), 463. 10.3390/pharmaceutics13040463 33805533 PMC8065980

[B94] de La Vega M.-A. Xiii A. Massey S. Spengler J. R. Kobinger G. P. Woolsey C. (2024). An update on nonhuman primate usage for drug and vaccine evaluation against filoviruses. Expert Opin. Drug Discov. 19 (10), 1185–1211. 10.1080/17460441.2024.2386100 39090822 PMC11466704

[B95] Lai A. K. Lo A. C. (2013). Animal models of diabetic retinopathy: summary and comparison. J. Diabetes Res. 2013, 106594. 10.1155/2013/106594 24286086 PMC3826427

[B96] Lee M. Yun S. Lee H. Yang J. (2017). Quercetin mitigates inflammatory responses induced by vascular endothelial growth factor in mouse retinal photoreceptor cells through suppression of nuclear factor kappa B. Int. J. Mol. Sci. 18 (11), 2497. 10.3390/ijms18112497 29165402 PMC5713462

[B97] Li H. Li M. Fu J. Ao H. Wang W. Wang X. (2021). Enhancement of oral bioavailability of quercetin by metabolic inhibitory nanosuspensions compared to conventional nanosuspensions. Drug Deliv. 28 (1), 1226–1236. 10.1080/10717544.2021.1927244 34142631 PMC8218931

[B98] Li M. Tang D. Yang T. Qian D. Xu R. (2021). Apoptosis triggering, an important way for natural products from herbal medicines to treat pancreatic cancers. Front. Pharmacol. 12, 796300. 10.3389/fphar.2021.796300 35222011 PMC8863938

[B99] Li Z. Zhao T. Li J. Yu Q. Feng Y. Xie Y. (2022). Nanomedicine based on natural products: improving clinical application potential. J. Nanomater. 2022 (1), 3066613. 10.1155/2022/3066613

[B100] Li S. Chen L. Fu Y. (2023). Nanotechnology-based ocular drug delivery systems: recent advances and future prospects. J. Nanobiotechnol. 21 (1), 232. 10.1186/s12951-023-01992-2 PMC10362606 37480102

[B101] Lin Y.-Y. Esswein P. Ramirez L. Warren E. Gerecht S. (2025). Derivation of functional retinal endothelial cells from human pluripotent stem cells for therapeutics and modeling. bioRxiv. 10.1101/2025.03.04.641453 PMC13526318 42380331

[B102] Liu H. Yang J. Du F. Gao X. Ma X. Huang Y. (2009). Absorption and disposition of ginsenosides after oral administration of Panax notoginseng extract to rats. Drug Metab. Dispos. 37 (12), 2290–2298. 10.1124/dmd.109.029819 19786509

[B103] Liu J. Li S. Li G. Li X. Yu C. Fu Z. (2019). Highly bioactive, bevacizumab-loaded, sustained-release PLGA/PCADK microspheres for intravitreal therapy in ocular diseases. Int. J. Pharm. 563, 228–236. 10.1016/j.ijpharm.2019.04.012 30959236

[B104] Liu J. Luo L. Xu F. Li G. Chen J. Teng L. (2020). Cyclic RGD peptide targeting coated nano drug Co-Delivery system for therapeutic use in age-related macular degeneration disease. Molecules 25 (21), 4897. 10.3390/molecules25214897 33113897 PMC7660171

[B105] Liu X. Huang K. Zhang F. Huang G. Wang L. Wu G. (2024). Multifunctional nano-in-micro delivery systems for targeted therapy in fundus neovascularization diseases. J. Nanobiotechnol. 22 (1), 354. 10.1186/s12951-024-02614-1 PMC11191225 38902775

[B106] Liu F. Zhao L. Wu T. Yu W. Li J. Wang W. (2024). Targeting autophagy with natural products as a potential therapeutic approach for diabetic microangiopathy. Front. Pharmacol. 15, 1364616. 10.3389/fphar.2024.1364616 38659578 PMC11039818

[B107] Loiseau A. Raîche-Marcoux G. Maranda C. Bertrand N. Boisselier E. (2023). Animal models in eye research: focus on corneal pathologies. Int. J. Mol. Sci. 24 (23), 16661. 10.3390/ijms242316661 38068983 PMC10706114

[B108] Long Y. Hu J. Liu Y. Wu D. Zheng Z. Gui S. (2024). Development of puerarin-loaded poly(lactic acid) microspheres for sustained ocular delivery: in vitro/vivo evaluation. Eur. J. Pharm. Biopharm. 204, 114524. 10.1016/j.ejpb.2024.114524 39370056

[B109] Lu M. Perez V. L. Ma N. Miyamoto K. Peng H. B. Liao J. K. (1999). VEGF increases retinal vascular ICAM-1 expression *in vivo* . Invest. Ophthalmol. Vis. Sci. 40 (8), 1808–1812. 10393052

[B110] Lu Y. Zhou N. Huang X. Cheng J. W. Li F. Q. Wei R. L. (2014). Effect of intravitreal injection of bevacizumab-chitosan nanoparticles on retina of diabetic rats. Int. J. Ophthalmol. 7 (1), 1–7. 10.3980/j.issn.2222-3959.2014.01.01 24634856 PMC3949450

[B111] Lu B. Lv X. Le Y. (2019). Chitosan-modified PLGA nanoparticles for control-released drug delivery. Polym. (Basel) 11 (2), 304. 10.3390/polym11020304 30960288 PMC6419218

[B112] Lu E. S. Cui Y. Le R. Zhu Y. Wang J. C. Laíns I. (2022). Detection of neovascularisation in the vitreoretinal interface slab using widefield swept-source optical coherence tomography angiography in diabetic retinopathy. Br. J. Ophthalmol. 106 (4), 534–539. 10.1136/bjophthalmol-2020-317983 33355148 PMC9092312

[B113] Lv T. Yu T. Fang Y. Zhang S. Jiang M. Zhang H. (2017). Role of generation on folic acid-modified poly(amidoamine) dendrimers for targeted delivery of baicalin to cancer cells. Mater Sci. Eng. C Mater Biol. Appl. 75, 182–190. 10.1016/j.msec.2016.12.134 28415453

[B114] Lv Y. Li H. Zhai B. T. Sun J. Cheng J. X. Zhang X. F. (2024a). Evidence of synergistic mechanisms of hepatoprotective botanical herbal preparation of Pueraria montana var. lobata and Schisandra sphenanthera. Front. Pharmacol. 15, 1412816. 10.3389/fphar.2024.1412816 38978983 PMC11228302

[B115] Lv Y. Zhai C. Sun G. He Y. (2024b). Chitosan as a promising materials for the construction of nanocarriers for diabetic retinopathy: an updated review. J. Biol. Eng. 18 (1), 18. 10.1186/s13036-024-00414-7 38388386 PMC10885467

[B116] Lv Y. Li W. Liao W. Jiang H. Liu Y. Cao J. (2024c). Nano-drug delivery systems based on natural products. Int. J. Nanomedicine 19, 541–569. 10.2147/ijn.s443692 38260243 PMC10802180

[B117] Lynch C. Kondiah P. P. D. Choonara Y. E. du Toit L. C. Ally N. Pillay V. (2019). Advances in biodegradable nano-sized polymer-based ocular drug delivery. Polym. (Basel) 11 (8), 1371. 10.3390/polym11081371 31434273 PMC6722735

[B118] Ma Y. Li T. (2021). Monitoring dynamic growth of retinal vessels in oxygen-induced retinopathy mouse model. J. Vis. Exp. 170, e62410. 10.3791/62410 33871456

[B119] Ma C. Yao M. D. Han X. Y. Shi Z. H. Yan B. Du J. L. (2022). Silencing of circular RNA-ZYG11B exerts a neuroprotective effect against retinal neurodegeneration. Int. J. Mol. Med. 50 (2), 106. 10.3892/ijmm.2022.5162 35730627 PMC9239035

[B120] Magalhães P. R. Reis P. B. P. S. Vila-Viçosa D. Machuqueiro M. Victor B. L. (2021). Identification of pan-assay INterference compoundS (PAINS) using an MD-Based protocol. Methods Mol. Biol. 2315, 263–271. 10.1007/978-1-0716-1468-6_15 34302681

[B121] Mahaling B. Low S. W. Y. Ch S. Addi U. R. Ahmad B. Connor T. B. (2023). Next-generation nanomedicine approaches for the management of retinal diseases. Pharmaceutics 15 (7), 2005. 10.3390/pharmaceutics15072005 37514191 PMC10383092

[B122] Malek G. Busik J. Grant M. B. Choudhary M. (2018). Models of retinal diseases and their applicability in drug discovery. Expert Opin. Drug Discov. 13 (4), 359–377. 10.1080/17460441.2018.1430136 29382242 PMC6192033

[B123] Mason R. H. Minaker S. A. Lahaie Luna G. Bapat P. Farahvash A. Garg A. (2022). Changes in aqueous and vitreous inflammatory cytokine levels in proliferative diabetic retinopathy: a systematic review and meta-analysis. Eye (Lond) 36, 2240‐2257. 10.1038/s41433-022-02127-x 35672457

[B124] Matter B. Bourne D. W. A. Kompella U. B. (2022). A high-throughput LC-MS/MS method for the simultaneous quantification of twenty-seven drug molecules in ocular tissues. AAPS PharmSciTech 23 (6), 192. 10.1208/s12249-022-02333-6 35819539

[B125] Maurya M. Liu C. H. Bora K. Kushwah N. Pavlovich M. C. Wang Z. (2024). Animal models of retinopathy of prematurity: advances and metabolic regulators. Biomedicines 12 (9), 1937. 10.3390/biomedicines12091937 39335451 PMC11428941

[B126] Mazzaferri J. Larrivée B. Cakir B. Sapieha P. Costantino S. (2018). A machine learning approach for automated assessment of retinal vasculature in the oxygen induced retinopathy model. Sci. Rep. 8 (1), 3916. 10.1038/s41598-018-22251-7 29500375 PMC5834630

[B127] McAnany J. J. Persidina O. S. Park J. C. (2022). Clinical electroretinography in diabetic retinopathy: a review. Surv. Ophthalmol. 67 (3), 712–722. 10.1016/j.survophthal.2021.08.011 34487740 PMC9158180

[B128] Méndez-Sánchez N. Dibildox-Martinez M. Sosa-Noguera J. Sánchez-Medal R. Flores-Murrieta F. J. (2019). Superior silybin bioavailability of silybin-phosphatidylcholine complex in oily-medium soft-gel capsules versus conventional silymarin tablets in healthy volunteers. BMC Pharmacol. Toxicol. 20 (1), 5. 10.1186/s40360-018-0280-8 30635055 PMC6330464

[B129] Metselaar J. M. Lammers T. (2020). Challenges in nanomedicine clinical translation. Drug Deliv. Transl. Res. 10 (3), 721–725. 10.1007/s13346-020-00740-5 32166632 PMC7228980

[B130] Meza-Rios A. Navarro-Partida J. Armendariz-Borunda J. Santos A. (2020). Therapies based on nanoparticles for eye drug delivery. Ophthalmol. Ther. 9 (3), 1–14. 10.1007/s40123-020-00257-7 32383107 PMC7406616

[B132] Mir M. Ahmed N. Rehman A. U. (2017). Recent applications of PLGA based nanostructures in drug delivery. Colloids Surf. B Biointerfaces 159, 217–231. 10.1016/j.colsurfb.2017.07.038 28797972

[B133] Mitton K. P. Deshpande M. Wong S. C. Guzman E. Cheng M. Dailey W. (2019). Retinal plasticity: functional recovery after bipolar cell loss in the oxygen induced retinopathy model. bioRxiv. 10.1101/2019.12.12.874271

[B134] Moiseev R. V. Morrison P. W. J. Steele F. Khutoryanskiy V. V. (2019). Penetration enhancers in ocular drug delivery. Pharmaceutics 11 (7), 321. 10.3390/pharmaceutics11070321 31324063 PMC6681039

[B135] Moiseev R. V. Kaldybekov D. B. Filippov S. K. Radulescu A. Khutoryanskiy V. V. (2022). Maleimide-decorated PEGylated mucoadhesive liposomes for ocular drug delivery. Langmuir 38 (45), 13870–13879. 10.1021/acs.langmuir.2c02086 36327096 PMC9671038

[B136] Mun E. A. Morrison P. W. J. Williams A. C. Khutoryanskiy V. V. (2014). On the barrier properties of the cornea: a microscopy study of the penetration of fluorescently labeled nanoparticles, polymers, and sodium fluorescein. Mol. Pharm. 11 (10), 3556–3564. 10.1021/mp500332m 25165886

[B137] Musazzi U. M. Franzè S. Condorelli F. Minghetti P. Caliceti P. (2023). Feeding next-generation nanomedicines to Europe: regulatory and quality challenges. Adv. Healthc. Mater 12 (30), e2301956. 10.1002/adhm.202301956 37718353 PMC11468706

[B138] Nair P. Aiello L. P. Gardner T. W. Jampol L. M. Ferris F. L. (2016). Report from the NEI/FDA diabetic retinopathy clinical trial design and endpoints workshop. Invest Ophthalmol. Vis. Sci. 57 (13), 5127–5142. 10.1167/iovs.16-20356 27699406 PMC6016432

[B139] Narayana S. Ahmed M. G. Gowda B. H. J. Shetty P. K. Nasrine A. Thriveni M. (2021). Recent advances in ocular drug delivery systems and targeting VEGF receptors for management of ocular angiogenesis: a comprehensive review. Future J. Pharm. Sci. 7 (1), 186. 10.1186/s43094-021-00331-2

[B140] Padule K. Shinde S. Chitlange S. Giram P. Nagore D. (2022). The advancement of herbal-based nanomedicine for hair. Cosmetics 9 (6), 118. 10.3390/cosmetics9060118

[B141] Pei K. Georgi M. Hill D. Lam C. F. J. Wei W. Cordeiro M. F. (2024). Review: neuroprotective nanocarriers in glaucoma. Pharm. (Basel) 17 (9), 1190. 10.3390/ph17091190 PMC11435059 39338350

[B142] Peltonen L. (2018). Practical guidelines for the characterization and quality control of pure drug nanoparticles and nano-cocrystals in the pharmaceutical industry. Adv. Drug Deliv. Rev. 131, 101‐115. 10.1016/j.addr.2018.06.009 29920294

[B143] Perlman J. I. Piltz J. Korte G. Tsai C. (2008). Endocytosis in the rat retinal pigment epithelium. Acta Anat. 135 (4), 354–360. 10.1159/000146781 2552737

[B144] Picaud S. Dalkara D. Marazova K. Goureau O. Roska B. Sahel J. A. (2019). The primate model for understanding and restoring vision. Proc. Natl. Acad. Sci. U. S. A. 116 (52), 26280–26287. 10.1073/pnas.1902292116 31871177 PMC6936588

[B145] Polewik K. Kosek M. Jamrozik D. Matuszek I. Smędowski A. Lewin-Kowalik J. (2023). Rodent models of diabetic retinopathy as a useful research tool to study neurovascular cross-talk. Biol. (Basel) 12 (2), 262. 10.3390/biology12020262 PMC9952991 36829539

[B146] Prajapati S. Maurya S. D. Das M. K. Tilak V. K. Verma K. K. Dhakar R. C. (2016). Dendrimers in drug delivery, diagnosis and therapy: basics and potential applications. J. Drug Deliv. Ther. 6, 67–92. 10.22270/jddt.v6i1.1190

[B147] Premanand C. Rema M. Sameer M. Z. Sujatha M. Balasubramanyam M. (2006). Effect of curcumin on proliferation of human retinal endothelial cells under *in vitro* conditions. Invest Ophthalmol. Vis. Sci. 47 (5), 2179–2184. 10.1167/iovs.05-0580 16639030

[B148] Qamar Z. Qizilbash F. F. Iqubal M. K. Ali A. Narang J. K. Ali J. (2019). Nano-based drug delivery system: recent strategies for the treatment of ocular disease and future perspective. Recent Pat. Drug Deliv. Formul. 13 (4), 246–254. 10.2174/1872211314666191224115211 31884933 PMC7499345

[B149] Qin S. Tan P. Xie J. Zhou Y. Zhao J. (2023). A systematic review of the research progress of traditional Chinese medicine against pulmonary fibrosis: from a pharmacological perspective. Chin. Med. 18 (1), 96. 10.1186/s13020-023-00797-7 37537605 PMC10398979

[B150] Qu X. Yu J. Bhagat G. Furuya N. Hibshoosh H. Troxel A. (2003). Promotion of tumorigenesis by heterozygous disruption of the beclin 1 autophagy gene. J. Clin. Invest 112 (12), 1809–1820. 10.1172/JCI20039 14638851 PMC297002

[B151] Rajan P. B. Koilpillai J. Narayanasamy D. (2024). Advancing ocular medication delivery with nano-engineered solutions: a comprehensive review of innovations, obstacles, and clinical impact. Cureus 16 (8), e66476. 10.7759/cureus.66476 39247042 PMC11381103

[B152] Raman R. Ramasamy K. Shah U. (2022). A paradigm shift in the management approaches of proliferative diabetic retinopathy: role of Anti-VEGF therapy. Clin. Ophthalmol. 16, 3005–3017. 10.2147/opth.s374165 36106093 PMC9467443

[B153] Ramos T. I. Villacis-Aguirre C. A. López-Aguilar K. V. Santiago Padilla L. Altamirano C. Toledo J. R. (2022). The Hitchhiker's guide to human therapeutic nanoparticle development. Pharmaceutics 14 (2), 247. 10.3390/pharmaceutics14020247 35213980 PMC8879439

[B154] Ranjbar S. Emamjomeh A. Sharifi F. Zarepour A. Aghaabbasi K. Dehshahri A. (2023). Lipid-based delivery systems for flavonoids and Flavonolignans: liposomes, nanoemulsions, and solid lipid nanoparticles. Pharmaceutics 15 (7), 1944. 10.3390/pharmaceutics15071944 37514130 PMC10383758

[B155] Rawat P. S. Ravi P. R. Mahajan R. R. (2024). Design, pharmacokinetic, and pharmacodynamic evaluation of a lecithin-chitosan hybrid nanoparticle-loaded dual-responsive *in situ* gel of nebivolol for effective treatment of glaucoma. Discov. Nano 19 (1), 156. 10.1186/s11671-024-04109-2 39331225 PMC11436582

[B156] Razavi M. S. Ebrahimnejad P. Fatahi Y. D’Emanuele A. Dinarvand R. (2022). Recent developments of nanostructures for the ocular delivery of natural compounds. Front. Chem. 10, 850757. 10.3389/fchem.2022.850757 35494641 PMC9043530

[B157] Reinboth M. Wolffram S. Abraham G. Ungemach F. R. Cermak R. (2010). Oral bioavailability of quercetin from different quercetin glycosides in dogs. Br. J. Nutr. 104 (2), 198–203. 10.1017/S000711451000053X 20230651

[B158] Rimpelä A. K. Garneau M. Baum-Kroker K. S. Schönberger T. Runge F. Sauer A. (2020). Quantification of drugs in distinctly separated ocular substructures of albino and pigmented rats. Pharmaceutics 12 (12), 1174. 10.3390/pharmaceutics12121174 33276439 PMC7760391

[B159] Robinson R. Barathi V. A. Chaurasia S. S. Wong T. Y. Kern T. S. (2012). Update on animal models of diabetic retinopathy: from molecular approaches to mice and higher mammals. Dis. Model Mech. 5 (4), 444–456. 10.1242/dmm.009597 22730475 PMC3380708

[B160] Rode F. Almholt K. Petersen M. Kreilgaard M. Kjalke M. Karpf D. M. (2018). Preclinical pharmacokinetics and biodistribution of subcutaneously administered glycoPEGylated recombinant factor VIII (N8-GP) and development of a human pharmacokinetic prediction model. J. Thromb. Haemost. 16 (6), 1141–1152. 10.1111/jth.14013 29582559

[B161] Rodríguez F. Caruana P. De la Fuente N. Español P. Gámez M. Balart J. (2022). Nano-based approved pharmaceuticals for cancer treatment: present and future challenges. Biomolecules 12 (6), 784. 10.3390/biom12060784 35740909 PMC9221343

[B162] Ruzafa N. Pereiro X. Lepper M. F. Hauck S. M. Vecino E. (2018). A proteomics approach to identify candidate proteins secreted by müller glia that protect ganglion cells in the retina. Proteomics 18 (11), 1700321. 10.1002/pmic.201700321 29645351

[B163] Sarkar T. Gogoi N. R. Jana B. K. Mazumder B. (2025). Formulation advances in posterior segment ocular drug delivery. J. Ocul. Pharmacol. Ther. 41 (3), 101–130. 10.1089/jop.2024.0153 39842469

[B164] Satyanarayana S. D. Abu Lila A. S. Moin A. Moglad E. H. Khafagy E. S. Alotaibi H. F. (2023). Ocular delivery of Bimatoprost-Loaded solid lipid nanoparticles for effective management of glaucoma. Pharm. (Basel) 16 (7), 1001. 10.3390/ph16071001 PMC10385266 37513913

[B165] Scheive M. Yazdani S. Hajrasouliha A. R. (2021). The utility and risks of therapeutic nanotechnology in the retina. Ther. Adv. Ophthalmol. 13, 25158414211003381. 10.1177/25158414211003381 33817552 PMC7989128

[B166] Semeraro F. Cancarini A. dell'Omo R. Rezzola S. Romano M. R. Costagliola C. (2015). Diabetic retinopathy: vascular and inflammatory disease. J. Diabetes Res. 2015, 582060. 10.1155/2015/582060 26137497 PMC4475523

[B167] Shan X. Gong X. Li J. Wen J. Li Y. Zhang Z. (2022). Current approaches of nanomedicines in the market and various stage of clinical translation. Acta Pharm. Sin. B 12 (7), 3028–3048. 10.1016/j.apsb.2022.02.025 35865096 PMC9293719

[B168] Sheng J. Tian X. Xu G. Wu Z. Chen C. Wang L. (2015). The hepatobiliary disposition of timosaponin b2 is highly dependent on influx/efflux transporters but not metabolism. Drug Metab. Dispos. 43 (1), 63–72. 10.1124/dmd.114.059923 25336752

[B169] Singh V. Ahmad R. (2011). THE challenges of ophthalmic drug delivery: a review. Int. J. Drug Deliv. 3, 56–62.

[B170] Slakter J. S. Schneebaum J. Shah S. A. (2015). Digital algorithmic diabetic retinopathy severity scoring system (An American ophthalmological Society Thesis). Trans. Am. Ophthalmol. Soc. 113, T9. 26681813 PMC4671510

[B171] Slimane M. (2017). Développement d’une approche multi-échelle pour l'étude de la solubilité des flavonoïdes et leur assemblage avec les polymères. Physics. 10.70675/32212cacz8b7ez4bfbz87f2zb9b7bae88b40

[B172] Smail S. S. (2024). *Ex Vivo* irritation evaluation of a novel brimonidine nanoemulsion using the Hen’s egg test on chorioallantoic membrane (HET-CAM). Cureus 16 (8), e68280. 10.7759/cureus.68280 39350816 PMC11440450

[B173] Suk J. S. Xu Q. Kim N. Hanes J. Ensign L. M. (2016). PEGylation as a strategy for improving nanoparticle-based drug and gene delivery. Adv. Drug Deliv. Rev. 99, 28–51. 10.1016/j.addr.2015.09.012 26456916 PMC4798869

[B174] Sull A. C. Vuong L. N. Price L. L. Srinivasan V. J. Gorczynska I. Fujimoto J. G. (2010). Comparison of spectral/Fourier domain optical coherence tomography instruments for assessment of normal macular thickness. Retina 30 (2), 235–245. 10.1097/IAE.0b013e3181bd2c3b 19952997 PMC2819609

[B175] Sun W. J. An X. D. Zhang Y. H. Zhao X. F. Sun Y. T. Yang C. Q. (2023). The ideal treatment timing for diabetic retinopathy: the molecular pathological mechanisms underlying early-stage diabetic retinopathy are a matter of concern. Front. Endocrinol. (Lausanne) 14, 1270145. 10.3389/fendo.2023.1270145 38027131 PMC10680169

[B176] Sun Y. Li Q. Huang Y. Yang Z. Li G. Sun X. (2024). Natural products for enhancing the sensitivity or decreasing the adverse effects of anticancer drugs through regulating the redox balance. Chin. Med. 19 (1), 110. 10.1186/s13020-024-00982-2 39164783 PMC11334420

[B177] Swetledge S. Jung J. P. Carter R. Sabliov C. (2021). Distribution of polymeric nanoparticles in the eye: implications in ocular disease therapy. J. Nanobiotechnology 19 (1), 10. 10.1186/s12951-020-00745-9 33413421 PMC7789499

[B178] Tabanelli R. Brogi S. Calderone V. (2021). Improving curcumin bioavailability: current strategies and future perspectives. Pharmaceutics 13 (10), 1715. 10.3390/pharmaceutics13101715 34684008 PMC8540263

[B179] Tang Z. Fan X. Chen Y. Gu P. (2022). Ocular nanomedicine. Adv. Sci. (Weinh) 9 (15), e2003699. 10.1002/advs.202003699 35150092 PMC9130902

[B180] Teo Z. L. Tham Y. C. Yu M. Chee M. L. Rim T. H. Cheung N. (2021). Global prevalence of diabetic retinopathy and projection of burden through 2045: systematic review and meta-analysis. Ophthalmology 128 (11), 1580–1591. 10.1016/j.ophtha.2021.04.027 33940045

[B181] Tewari A. K. Upadhyay S. C. Kumar M. Pathak K. Kaushik D. Verma R. (2022). Insights on development aspects of polymeric nanocarriers: the translation from bench to clinic. Polym. (Basel) 14 (17), 3545. 10.3390/polym14173545 PMC9459741 36080620

[B182] Ting D. S. W. Cheung C. Y. L. Lim G. Tan G. S. W. Quang N. D. Gan A. (2017). Development and validation of a deep learning system for diabetic retinopathy and related eye diseases using retinal images from multiethnic populations with diabetes. JAMA 318 (22), 2211–2223. 10.1001/jama.2017.18152 29234807 PMC5820739

[B183] Tsai-hsuan Ku S. (2012). Forming interdisciplinary expertise: one organization's journey on the road to translational nanomedicine. Wiley Interdiscip. Rev. Nanomed Nanobiotechnol 4 (4), 366–377. 10.1002/wnan.1172 22517677 PMC3380180

[B184] Tyagi P. Kadam R. S. Kompella U. B. (2012). Comparison of suprachoroidal drug delivery with subconjunctival and intravitreal routes using noninvasive fluorophotometry. PLoS One 7 (10), e48188. 10.1371/journal.pone.0048188 23118950 PMC3485142

[B185] Vähätupa M. Järvinen T. A. H. Uusitalo-Järvinen H. (2020). Exploration of oxygen-induced retinopathy model to discover new therapeutic drug targets in retinopathies. Front. Pharmacol. 11, 873. 10.3389/fphar.2020.00873 32595503 PMC7300227

[B186] Velumani R. Bama S. Jose M. V. (2021). “Deep image prior and structural variation-based super-resolution network for Fluorescein fundus angiography images,” in Computational Intelligence Methods for Super-Resolution in Image Processing Applications. Editors Deshpande A. Estrela V. V. Razmjooy N. (Cham: Springer International Publishing), 191–208.

[B187] Vezina M. (2013). Pre-clinical testing: good laboratory practice and the requirements imposed by the agencies. Acta Ophthalmol. 91 (252). 10.1111/j.1755-3768.2013.4412.x

[B188] Villacampa P. Menger K. E. Abelleira L. Ribeiro J. Duran Y. Smith A. J. (2017). Accelerated oxygen-induced retinopathy is a reliable model of ischemia-induced retinal neovascularization. PLoS One 12 (6), e0179759. 10.1371/journal.pone.0179759 28650964 PMC5484470

[B189] Vujosevic S. Limoli C. Nucci P. (2024). Novel artificial intelligence for diabetic retinopathy and diabetic macular edema: what is new in 2024? Curr. Opin. Ophthalmol. 35 (6), 472–479. 10.1097/ICU.0000000000001084 39259647 PMC11426980

[B190] Walle T. (2011). Bioavailability of resveratrol. Ann. N. Y. Acad. Sci. 1215, 9–15. 10.1111/j.1749-6632.2010.05842.x 21261636

[B191] Walle T. Hsieh F. DeLegge M. H. Oatis J. E. Walle U. K. (2004). High absorption but very low bioavailability of oral resveratrol in humans. Drug Metab. Dispos. 32 (12), 1377–1382. 10.1124/dmd.104.000885 15333514

[B192] Wan Z. W. Sun P. Song S. Y. Wang Y. Song Y. Deng B. (2025). Resveratrol ameliorates early retinal neurodegeneration in diabetic retinopathy via microRNA-29b/specificity protein 1/apoptosis pathway by enhancing autophagy. Eur. J. Nutr. 64 (5), 232. 10.1007/s00394-025-03751-5 40608078

[B193] Wang W. Lo A. C. Y. (2018). Diabetic retinopathy: pathophysiology and treatments. Int. J. Mol. Sci. 19 (6), 1816. 10.3390/ijms19061816 29925789 PMC6032159

[B194] Wang N. Wei L. Liu D. Zhang Q. Xia X. Ding L. (2022). Identification and validation of autophagy-related genes in diabetic retinopathy. Front. Endocrinol. (Lausanne) 13, 867600. 10.3389/fendo.2022.867600 35574010 PMC9098829

[B195] Wang Z. Zhang N. Lin P. Xing Y. Yang N. (2024). Recent advances in the treatment and delivery system of diabetic retinopathy. Front. Endocrinol. (Lausanne) 15, 1347864. 10.3389/fendo.2024.1347864 38425757 PMC10902204

[B196] Wei Q. Y. Xu Y. M. Lau A. T. Y. (2020). Recent progress of nanocarrier-based therapy for solid malignancies. Cancers (Basel) 12 (10), 2783. 10.3390/cancers12102783 32998391 PMC7600685

[B197] Wei L. Sun X. Fan C. Li R. Zhou S. Yu H. (2022). The pathophysiological mechanisms underlying diabetic retinopathy. Front. Cell Dev. Biol. 10, 963615. 10.3389/fcell.2022.963615 36111346 PMC9468825

[B198] Wu J. H. Li Y. N. Chen A. Q. Hong C. D. Zhang C. L. Wang H. L. (2020). Inhibition of Sema4D/PlexinB1 signaling alleviates vascular dysfunction in diabetic retinopathy. EMBO Mol. Med. 12 (2), e10154. 10.15252/emmm.201810154 31943789 PMC7005627

[B199] Wu C. Lee S. L. Taylor C. Li J. Chan Y. M. Agarwal R. (2020). Scientific and regulatory approach to botanical drug development: a U.S. FDA perspective. J. Nat. Prod. 83 (2), 552–562. 10.1021/acs.jnatprod.9b00949 31977211

[B200] Wu X. Chua J. Ho C. Yao X. Muralidharan A. R. Najjar R. P. (2022). In-vivo imaging of ocular microvasculature using swept-source optical coherence tomography angiography in seven types of lab animals. Front. Photonics 3, 867594. 10.3389/fphot.2022.867594

[B201] Wu Y. Li X. Fu X. Huang X. Zhang S. Zhao N. (2024). Innovative nanotechnology in drug delivery systems for advanced treatment of posterior segment ocular diseases. Adv. Sci. (Weinh) 11 (32), e2403399. 10.1002/advs.202403399 39031809 PMC11348104

[B202] Xiao S. Bucher F. Wu Y. Rokem A. Lee C. S. Marra K. V. (2017). Fully automated, deep learning segmentation of oxygen-induced retinopathy images. JCI Insight 2 (24), e97585. 10.1172/jci.insight.97585 29263301 PMC5752269

[B203] Xu Q. F. Fang X. L. Chen D. F. (2003). Pharmacokinetics and bioavailability of ginsenoside Rb1 and Rg1 from Panax notoginseng in rats. J. Ethnopharmacol. 84 (2-3), 187–192. 10.1016/s0378-8741(02)00317-3 12648814

[B204] Xu X. Tang Q. Gao Y. Chen S. Yu Y. Qian H. (2025). Recent developments in the fabrication of food microparticles and nanoparticles using microfluidic systems. Crit. Rev. Food Sci. Nutr. 65 (12), 2199–2213. 10.1080/10408398.2024.2329967 38520155

[B205] Yang C. Merlin D. (2023). Challenges to safe nanomedicine treatment. Nanomater. (Basel) 13 (7), 1171. 10.3390/nano13071171 PMC10096857 37049268

[B206] Yang L. P. Sun H. l. Wu L. m. Guo X. j. Dou H. l. Tso M. O. M. (2009). Baicalein reduces inflammatory process in a rodent model of diabetic retinopathy. Invest Ophthalmol. Vis. Sci. 50 (5), 2319–2327. 10.1167/iovs.08-2642 19011009

[B207] Yang F. Yu J. Ke F. Lan M. Li D. Tan K. (2018). Curcumin alleviates diabetic retinopathy in experimental diabetic rats. Ophthalmic Res. 60 (1), 43–54. 10.1159/000486574 29597206

[B208] Yang C. Wu Q. Liu J. Mo J. Li X. Yang C. (2022). Intelligent wireless theranostic contact lens for electrical sensing and regulation of intraocular pressure. Nat. Commun. 13 (1), 2556. 10.1038/s41467-022-29860-x 35581184 PMC9114010

[B209] Yang C. Yu Y. An J. (2024). Effect of high-sucrose diet on the occurrence and progression of diabetic retinopathy and dietary modification strategies. Nutrients 16 (9), 1393. 10.3390/nu16091393 38732638 PMC11085904

[B210] Ye X. Fung N. S. K. Lam W. C. Lo A. C. Y. (2024). Nutraceuticals for diabetic retinopathy: recent advances and novel delivery systems. Nutrients 16 (11), 1715. 10.3390/nu16111715 38892648 PMC11174689

[B211] Younis M. A. Tawfeek H. M. Abdellatif A. A. Abdel-Aleem J. A. Harashima H. (2022). Clinical translation of nanomedicines: challenges, opportunities, and keys. Adv. Drug Deliv. Rev. 181, 114083. 10.1016/j.addr.2021.114083 34929251

[B212] Zamboulis A. Nanaki S. Michailidou G. Koumentakou I. Lazaridou M. Ainali N. M. (2020). Chitosan and its derivatives for ocular delivery formulations: recent advances and developments. Polym. (Basel) 12 (7), 1519. 10.3390/polym12071519 32650536 PMC7407599

[B213] Zeng L. Ma W. Shi L. Chen X. Wu R. Zhang Y. (2019). Poly(lactic-co-glycolic acid) nanoparticle-mediated interleukin-12 delivery for the treatment of diabetic retinopathy. Int. J. Nanomedicine 14, 6357–6369. 10.2147/IJN.S214727 31496691 PMC6690602

[B214] Zeng M. Guo D. Fernández-Varo G. Zhang X. Fu S. Ju S. (2023). The integration of nanomedicine with traditional Chinese medicine: drug delivery of natural products and other opportunities. Mol. Pharm. 20 (2), 886–904. 10.1021/acs.molpharmaceut.2c00882 36563052

[B215] Zhang J. Diamond J. S. (2006). Distinct perisynaptic and synaptic localization of NMDA and AMPA receptors on ganglion cells in rat retina. J. Comp. Neurol. 498 (6), 810–820. 10.1002/cne.21089 16927255 PMC2577313

[B216] Zhang B. Fang C. Y. Chang C. C. Peterson R. Maswadi S. Glickman R. D. (2012). Photoacoustic emission from fluorescent nanodiamonds enhanced with gold nanoparticles. Biomed. Opt. Express 3 (7), 1662–1669. 10.1364/BOE.3.001662 22808436 PMC3395489

[B217] Zhang H. T. Shi K. Baskota A. Zhou F. L. Chen Y. X. Tian H. M. (2014). Silybin reduces obliterated retinal capillaries in experimental diabetic retinopathy in rats. Eur. J. Pharmacol. 740, 233–239. 10.1016/j.ejphar.2014.07.033 25066112

[B218] Zhang H. W. Zhang H. Grant S. J. Wan X. Li G. (2018). Single herbal medicine for diabetic retinopathy. Cochrane Database Syst. Rev. 12 (12), Cd007939. 10.1002/14651858.CD007939.pub2 30566763 PMC6517038

[B219] Zhang X. Aixinjueluo Q. Y. Li S. Y. Song L. L. Lau C. T. Tan R. (2019). Reporting quality of Cochrane systematic reviews with Chinese herbal medicines. Syst. Rev. 8 (1), 302. 10.1186/s13643-019-1218-y 31796121 PMC6892158

[B220] Zhang J. Jiao J. Niu M. Gao X. Zhang G. Yu H. (2021). Ten years of knowledge of nano-carrier based drug delivery systems in ophthalmology: current evidence, challenges, and future prospective. Int. J. Nanomed. 16, 6497–6530. 10.2147/ijn.s329831 PMC8473849 34588777

[B221] Zhang M. Zhou M. Cai X. Zhou Y. Jiang X. Luo Y. (2022). VEGF promotes diabetic retinopathy by upregulating the PKC/ET/NF-κB/ICAM-1 signaling pathway. Eur. J. Histochem. 66 (4), 3255. 10.4081/ejh.2022.3522 PMC9667573 36305269

[B222] Zhang Z. Deng C. Paulus Y. M. (2024). Advances in structural and functional retinal imaging and biomarkers for early detection of diabetic retinopathy. Biomedicines 12 (7), 1405. 10.3390/biomedicines12071405 39061979 PMC11274328

[B223] Zhao L. Chen G. Li J. Fu Y. Mavlyutov T. A. Yao A. (2017). An intraocular drug delivery system using targeted nanocarriers attenuates retinal ganglion cell degeneration. J. Control Release 247, 153–166. 10.1016/j.jconrel.2016.12.038 28063892 PMC5323250

[B224] Zheng T. Jiang T. Huang Z. Ma H. Wang M. (2023). Role of traditional Chinese medicine monomers in cerebral ischemia/reperfusion injury:a review of the mechanism. Front. Pharmacol. 14, 1220862. 10.3389/fphar.2023.1220862 37654609 PMC10467294

[B225] Zhong M. Tian X. Chen S. Chen M. Guo Z. Zhang M. (2019). Identifying the active components of Baihe-Zhimu decoction that ameliorate depressive disease by an effective integrated strategy: a systemic pharmacokinetics study combined with classical depression model tests. Chin. Med. 14, 37. 10.1186/s13020-019-0254-9 31572489 PMC6757420

[B226] Zhu X. Ding G. Ren S. Xi J. Liu K. (2024). The bioavailability, absorption, metabolism, and regulation of glucolipid metabolism disorders by quercetin and its important glycosides: a review. Food Chem. 458, 140262. 10.1016/j.foodchem.2024.140262 38944925

[B227] Zhu J. Lee H. Huang R. Zhou J. Zhang J. Yang X. (2024). Harnessing nanotechnology for cancer treatment. Front. Bioeng. Biotechnol. 12, 1514890. 10.3389/fbioe.2024.1514890 39902172 PMC11788409

[B228] Zielińska A. Soles B. B. Lopes A. R. Vaz B. F. Rodrigues C. M. Alves T. F. R. (2020). Nanopharmaceuticals for eye administration: sterilization, depyrogenation and clinical applications. Biol. (Basel) 9 (10), 336. 10.3390/biology9100336 33066555 PMC7602230

[B229] Zingg R. Fischer M. (2019). The consolidation of nanomedicine. Wiley Interdiscip. Rev. Nanomed Nanobiotechnol 11 (6), e1569. 10.1002/wnan.1569 31240855 PMC6852524

