# Supplementary material for: Nanomaterial-enabled delivery of plant-derived bioactive metabolites for diabetic retinopathy: from evidence appraisal to preclinical translation
Source: Front Pharmacol. 2026 Jun 26;17:1809139. doi: 10.3389/fphar.2026.1809139 (PMC13349831; doi:10.3389/fphar.2026.1809139)
Supplement: Supplementary file 1 [file Table1.docx]

### Table S1. Taxonomic verification and botanical-material reporting of representative plant-derived metabolites or botanical sources discussed in this review

*Plant species names were validated against Medicinal Plant Names Services (MPNS, Royal Botanic Gardens, Kew), Plants of the World Online (POWO), and/or World Flora Online (WFO). For purified metabolites without a specified botanical source, taxonomic verification was treated as not applicable; for botanical preparations, extracts, and multi-component formulations, accepted scientific name, family, taxonomic authority, plant part, and preparation details were required.*

| **Metabolite** | **Botanical source reported or commonly associated source to be verified** | **Accepted scientific name** | **Family** | **Taxonomic authority** | **Plant part / source material** | **Verification source** | **Reporting status in the reviewed manuscript** | **Comment for evidence appraisal** |
| --- | --- | --- | --- | --- | --- | --- | --- | --- |
| Quercetin | No single botanical source specified in the manuscript; often discussed as a purified flavonoid | N/A for purified compound. | N/A | N/A | N/A for purified compound | N/A unless original study specifies source | Purified metabolite; botanical source not defined | Keep as quercetin, a purified plant-derived flavonoid. Do not imply a specific botanical origin unless the original study reports it. |
| Puerarin | Gegen / kudzu root source; manuscript discusses puerarin as a purified metabolite | Pueraria montana var. lobata (Willd.) Maesen & S.M.Almeida ex Sanjappa & Predeep | Fabaceae | (Willd.) Maesen & S.M.Almeida ex Sanjappa & Predeep | Root | POWO | Partly defined; article focuses on purified puerarin rather than extract details | If original studies use “Pueraria lobata” or “kudzu,” verify synonymy and accepted name. POWO lists Pueraria montana var. lobata as accepted. |
| Baicalin | Huangqin / Chinese skullcap source; manuscript discusses baicalin as purified metabolite and nanodrops/liposomes | Scutellaria baicalensis Georgi | Lamiaceae | Georgi | Root | POWO / WFO | Partly defined; botanical source details not systematically reported | If original study uses purified baicalin, chemical identity/purity is more important; if extract-based, root source and extraction method must be reported. POWO lists Scutellaria baicalensis Georgi as accepted; WFO reports it as accepted in Lamiaceae. |
| Silibinin / silymarin | Milk thistle | Silybum marianum (L.) Gaertn. | Asteraceae | (L.) Gaertn. | Fruit / seed | POWO | Partly defined; manuscript mentions silibinin/silymarin as lipophilic metabolites | Distinguish purified silibinin from silymarin extract. POWO lists Silybum marianum (L.) Gaertn. as accepted and notes medicinal use. |
| Curcumin / curcuminoids | Turmeric | Curcuma longa L. | Zingiberaceae | L. | Rhizome | POWO | Partly defined; curcumin appears mainly as purified metabolite or nanoformulation payload | If “curcuminoids” or turmeric extract is discussed, report extract profile and marker compounds. POWO identifies Curcuma longa L. as accepted; Kew notes it belongs to Zingiberaceae and its rhizomes are the source of turmeric. |
| Resveratrol | No single source specified; possible source includes grape or Japanese knotweed depending on original study | N/A for purified compound unless source is specified | N/A | N/A | N/A | N/A unless original study specifies source | Purified metabolite; botanical source not defined | Keep as purified resveratrol unless original study states grape-, knotweed-, or other plant-derived material. |
| Resveratrol: possible grape source if specified | Grape | Vitis vinifera L. | Vitaceae | L. | Fruit / skin / seed depending on preparation | POWO | Not specified in manuscript | Use only if the original study reports grape-derived resveratrol. POWO lists Vitis vinifera L. as accepted; Vitaceae is the family. |
| Resveratrol: possible knotweed source if specified | Japanese knotweed / Hu Zhang | Reynoutria japonica Houtt. | Polygonaceae | Houtt. | Rhizome / root | POWO | Not specified in manuscript | If original study uses “Polygonum cuspidatum,” update to accepted name Reynoutria japonica Houtt. POWO lists Polygonum cuspidatum as a synonym under accepted Reynoutria japonica. |
| Berberine | No single source specified; possible source includes Huanglian / Coptis or Berberis depending on original study | N/A for purified berberine unless source is specified | N/A | N/A | N/A | N/A unless original study specifies source | Purified metabolite in manuscript context | Keep as purified berberine unless the original study reports an extract or source species. |
| Berberine: possible Coptis source if specified | Huanglian | Coptis chinensis Franch. | Ranunculaceae | Franch. | Rhizome / root | POWO | Not specified in manuscript | Use when berberine is sourced from Huanglian/Coptis. POWO lists Coptis chinensis Franch. as an accepted name. |
| Berberine: possible Berberis source if specified | Barberry | Berberis vulgaris L. | Berberidaceae | L. | Root bark / stem bark depending on preparation | POWO | Not specified in manuscript | Use only if the original study specifies Berberis vulgaris. POWO lists Berberis vulgaris L. as accepted. |
| Tetramethylpyrazine / ligustrazine | Chuanxiong source; manuscript discusses tetramethylpyrazine PK / retinal accumulation | Conioselinum anthriscoides ‘Chuanxiong’ | Apiaceae | Cultivar name under Conioselinum anthriscoides; check cultivar authority if required | Rhizome | POWO / MPNS | Partly defined; manuscript discusses purified TMP, not full extract | Avoid older ambiguous naming such as Ligusticum chuanxiong without verification. POWO treats Ligusticum chuanxiong as a synonym of Conioselinum anthriscoides ‘Chuanxiong’; POWO lists this as accepted. |
| Tetramethylpyrazine: possible older synonym in literature | Chuanxiong sometimes reported as Ligusticum striatum or Ligusticum wallichii | Oreocome striata (DC.) Pimenov & Kljuykov | Apiaceae | (DC.) Pimenov & Kljuykov | Root / rhizome depending on study | POWO | Not used as preferred source in manuscript | Use only if original study explicitly uses Ligusticum striatum; POWO treats Ligusticum striatum as synonym of Oreocome striata. This is a taxonomic ambiguity and should be noted. |
| Notoginsenosides / Panax notoginseng-derived compounds | Sanqi / notoginseng | Panax notoginseng (Burkill) F.H.Chen | Araliaceae | (Burkill) F.H.Chen | Root / rhizome | POWO | Partly defined; manuscript notes poor oral absorption and lack of retinal-targeting data | For notoginsenoside mixtures, report marker compounds such as notoginsenoside R1, ginsenoside Rg1, and ginsenoside Rb1 where applicable. POWO lists Panax notoginseng as accepted. |
| Hydroxysafflor yellow A (HSYA) | Safflower / Honghua | Carthamus tinctorius L. | Asteraceae | L. | Flower | POWO / WFO | Mentioned as HSYA; botanical source details not provided | If HSYA is discussed as a purified compound, chemical identity/purity should be reported; if safflower extract is used, flower source and marker profile are required. POWO and WFO list Carthamus tinctorius L. as accepted. |
| Gypenoside XVII / Gyp-17 | Jiaogulan | Gynostemma pentaphyllum (Thunb.) Makino | Cucurbitaceae | (Thunb.) Makino | Aerial parts / whole herb depending on study | POWO | Partly defined; manuscript discusses Gyp-17 in nanocarriers | If gypenoside fraction or extract is used, marker profile and batch consistency are required. POWO lists Gynostemma pentaphyllum (Thunb.) Makino as accepted. |
| Norkurarinone | Kushen-associated flavonoid source | Sophora flavescens Aiton | Fabaceae | Aiton | Root | POWO / WFO | Partly defined; manuscript discusses norkurarinone mechanistically | If pure norkurarinone is used, source species is less critical than purity; if extract-derived, root source and chemical profiling are required. POWO lists Sophora flavescens Aiton as accepted; WFO reports it as accepted in Fabaceae. |
| Epigallocatechin-3-gallate / EGCG | Tea | Camellia sinensis (L.) Kuntze | Theaceae | (L.) Kuntze | Leaf | POWO | Partly defined; manuscript discusses EGCG / epigallocatechin-conjugated nanoparticles | Distinguish purified EGCG from green tea extract. POWO lists Camellia sinensis (L.) Kuntze as accepted. |
| Hyperforin / St. John’s wort | St. John’s wort | Hypericum perforatum L. | Hypericaceae | L. | Flowering aerial parts | POWO / WFO | Mentioned for CYP3A4 / P-gp interaction; not a DR therapeutic candidate | Included because manuscript uses St. John’s wort as an herb–drug interaction example. POWO and WFO list Hypericum perforatum L. as accepted; POWO gives family as Hypericaceae. |
| Timosaponin AIII / timosaponins BII and BIII | Zhimu source commonly used in TCM literature | Anemarrhena asphodeloides Bunge | Asparagaceae | Bunge | Rhizome | POWO / original-study verification needed | Manuscript discusses timosaponin metabolism / CAR activation, but botanical source is not defined | Add this row if the original cited timosaponin study derives compounds from Zhimu or discusses botanical source. If source is synthetic / commercial purified compound, mark plant source as N/A. |
| Genistein | Soybean or other legume source depending on original study | Glycine max (L.) Merr. | Fabaceae | (L.) Merr. | Seed | POWO / source-specific verification needed | Manuscript mentions genistein only as an example of hydrophobic payload for dendrimers | Do not imply soybean origin unless original study specifies it. Use as a representative source only. |
| Kaempferol | No source specified in manuscript | N/A for purified compound | N/A | N/A | N/A | N/A unless original study specifies source | Mentioned as flavonoid example; source not defined | Keep as purified flavonoid or plant-derived compound; do not assign a plant species without original-study evidence. |
| Taxifolin dehydrate nanoparticles | No source specified in manuscript | N/A for purified compound | N/A | N/A | N/A | N/A unless original study specifies source | Used as solubility / nanoencapsulation example | Taxifolin occurs in multiple species; do not assign a botanical source unless original study reports it. |
| Lutein-loaded nanoemulsions | No single source specified; lutein can be dietary / plant-derived carotenoid | N/A for purified lutein unless source is specified | N/A | N/A | N/A | N/A unless original study specifies source | Mentioned as nanoformulated compound in DR model | Keep as purified lutein. If from marigold or another botanical source, verify that source separately. |
| PHF-dia / polyherbal formulation | Multi-component formulation | Full list of species required | Full family list required | Full taxonomic authorities required | Plant parts and ratios required | MPNS / POWO / original formulation paper | Insufficiently defined in current manuscript | This formulation should not be used as strong pharmacological evidence unless all plant species, plant parts, extraction / preparation method, marker compounds, and batch consistency are reported. |
| Chinese botanical medicine interventions / formulations in systematic reviews | Multi-component or variable formulations | Full accepted names required for all species | Required for all species | Required for all species | Required for all ingredients | MPNS / POWO | Not individually resolved in current manuscript | These studies should be discussed as low-certainty or heterogeneous evidence unless composition, dosage, and botanical identity are fully reported. |

**Table S2. Plant material and initial-processing reporting checklist**

| **Section / Topic** | **Checklist item** | **Applicability / Reporting status** | **Comment for this review** |
| --- | --- | --- | --- |
| Plant identity | Full scientific name, family, and taxonomic authority reported | Applicable to botanical preparations; partly addressed / assessed where source was reported | This review focuses mainly on purified metabolites. For extract/formulation studies, plant identity was considered during evidence appraisal. |
| Plant part / source | Plant part and source/geographical origin reported | Applicable to extracts/formulations; often NR in purified-metabolite studies | Not applicable to purified commercial metabolites unless the original study reported botanical source. Required for botanical preparations/extracts. |
| Initial processing | Harvesting, drying, storage, and processing described | Mostly N/A for purified metabolites; NR or downgraded for poorly defined extracts | Not applicable to purified metabolites. Extract-based studies lacking this information were downgraded. |
| Extraction / preparation | Extraction solvent, method, ratio, yield, and preparation procedure reported | Applicable to extract studies; NR led to evidence downgrading | Not applicable to purified metabolites. Required for botanical extracts or multi-component formulations. |
| Formulation | Final preparation/formulation and batch consistency described | Partly addressed; nanoformulation studies assessed for particle characterization and batch consistency where reported | Nanoformulation studies were assessed for particle size, PDI, zeta potential, drug loading, release profile, sterility, and batch consistency. |

**Table S3. Analytical-methods reporting checklist for extract type A**

| **Section / Topic** | **Checklist item** | **Yes / Partly** | **Location** | **Comment for this review** |
| --- | --- | --- | --- | --- |
| Extract type | Confirm whether the botanical drug is covered by a national/regional pharmacopoeial monograph | Partly | Section 2 | This is a review, not a new extract study. Applied only when original studies involved botanical preparations/extracts. |
| Pharmacopoeial compliance | Active ingredients or marker compounds described according to monograph | Partly | Section 2 | For purified metabolites, chemical identity and purity were prioritized instead. For extracts, lack of marker information was considered a limitation. |
| Analytical method | Chemical fingerprinting or chromatographic/spectroscopic characterization reported | Partly | Section 2; Section 6.1 | Studies lacking HPLC/LC-MS/HPTLC/NMR or equivalent profiling were downgraded. |
| Marker quantification | At least two marker compounds quantified where applicable | Partly | Section 2 | Not applicable to single purified compounds. For extracts/formulations, absence of marker quantification limited reproducibility. |
| Reference standards | Reference standards or direct comparison with official standards reported | Partly | Section 2 | Required for strong evidence. Studies without reference standards were treated as lower-quality evidence. |

### Table S4. Pharmacological and translational appraisal framework for included studies, by study / material category

| **Study / material category** | **Material definition required** | **Dose / concentration information required** | **Lowest effective dose / concentration** | **Model used and model relevance** | **Required controls** | **PK / biodistribution requirement** | **Safety requirement** | **Main limitation if missing** | **Interpretation in this review** |
| --- | --- | --- | --- | --- | --- | --- | --- | --- | --- |
| Purified plant-derived bioactive metabolites | Chemical identity, purity, source, analytical confirmation, and stability of the purified compound should be reported. Representative examples include quercetin, puerarin, baicalein, silibinin, curcumin, resveratrol, berberine, tetramethylpyrazine, and notoginsenosides. | In vitro concentration range or in vivo dose range should be reported, together with exposure duration, dosing frequency, and route of administration. | Should be extracted when reported. If not reported, record as NR; no lowest effective dose/concentration identified. | High relevance when tested in retinal endothelial cells, RPE cells, Müller cells, retinal ganglion cells, iBRB models, STZ diabetic rodents, OIR models, or other DR-relevant systems. Lower relevance when tested only in generic non-retinal cell lines or non-DR disease models. | Vehicle control, disease-model control, positive comparator where available, and ideally a clinically relevant comparator such as anti-VEGF or corticosteroid therapy. | Required when retinal delivery, ocular exposure, or posterior-segment activity is claimed. Retina/choroid, vitreous, aqueous humor, cornea, and plasma concentrations should be compared over time. | Cytotoxicity, ocular tolerance, inflammation, and systemic toxicity should be assessed, especially when high systemic doses are used. | Without dose-response, model relevance, or ocular exposure, pharmacological claims cannot establish clinically meaningful retinal activity. | Treated as preclinical or hypothesis-generating evidence unless supported by dose-response, target engagement, ocular PK, and in vivo validation. |
| Metabolite-loaded nanoformulations | Both the active metabolite and carrier system must be defined. Required carrier attributes include composition, particle size, PDI, zeta potential, morphology, encapsulation efficiency, drug loading, release kinetics, stability, and sterility/endotoxin status where relevant. | Dose of the active metabolite and amount/concentration of the carrier should both be reported. Release medium and release duration should also be specified. | Should be reported as active-metabolite equivalent dose/concentration, not only nanoparticle mass. If unavailable, record as NR. | High relevance when tested in retinal barrier models, ex vivo ocular penetration models, STZ/OIR models, or in vivo ocular delivery systems. Limited relevance if only formulation characterization or generic uptake assays are reported. | Free metabolite, blank carrier, untreated/disease control, and non-targeted carrier control when active targeting is claimed. | Essential. Retinal targeting or enhanced delivery should not be claimed without quantitative ocular biodistribution, ideally including retina/choroid-to-plasma or vitreous-to-plasma comparison. | Blank-carrier toxicity, metabolite-loaded carrier toxicity, ocular irritation, inflammation, histology, and repeated-dose tolerance should be assessed. | Without blank-carrier and free-metabolite controls, the contribution of the carrier cannot be separated from the active compound. Without ocular PK, enhanced uptake does not prove retinal targeting. | Considered enabling formulation evidence unless linked to retinal exposure, DR-relevant efficacy endpoints, and safety data. |
| Botanical extracts or botanical preparations | Full botanical definition is required: accepted scientific name, family, taxonomic authority, plant part, geographical origin, extraction/processing method, extract type, chemical profile, marker compounds, and batch-to-batch consistency. | Extract dose, marker-compound content, solvent system, extraction yield, and treatment duration should be reported. | Should be expressed as both total extract dose and marker-compound equivalent where possible. If marker content is absent, record as NR; pharmacological attribution limited. | Relevant only when tested in DR-related in vitro or in vivo models. Studies using poorly defined extracts in non-retinal models have limited translational value. | Vehicle control, disease control, positive comparator, and preferably purified-marker comparator. | Required if retinal or systemic pharmacological activity is claimed. Marker-compound PK is preferred. | Acute and repeated-dose toxicity, ocular tolerance, systemic toxicity, and potential herb-drug interaction assessment are needed. | Undefined extracts cannot be reproduced, compared, or confidently linked to specific mechanisms. Evidence is downgraded if taxonomy, extraction method, chemical profile, or marker quantification is missing. | Used only as supportive contextual evidence unless composition and pharmacological attribution are adequately defined. |
| Multi-component botanical formulations | Complete formulation composition is required, including all species, family, taxonomic authority, plant parts, component ratios, processing method, chemical profile, and marker compounds. Species names should be verified using MPNS/POWO or equivalent databases. | Total formulation dose and individual marker-compound doses should be reported. Batch consistency should be demonstrated. | Should be reported for the formulation and, where possible, for major marker compounds. If not reported, record as NR. | Clinical or preclinical DR relevance depends on whether the model reflects retinal vascular leakage, inflammation, neurodegeneration, or BRB dysfunction. | Placebo/vehicle, standard-care comparator, disease control, and ideally formulation-minus-one or marker-compound controls. | Required for mechanistic or retinal-targeting claims. Without systemic and ocular exposure data, tissue-level activity remains uncertain. | Systemic toxicity, liver/kidney function, ocular safety, immunogenicity, and interaction risk should be evaluated. | Multi-component formulations cannot support compound-specific mechanisms unless composition, marker compounds, and exposure are defined. | Used only as supportive contextual evidence unless composition and pharmacological attribution are adequately defined. |
| Single-concentration in vitro studies | Compound identity and purity must be defined; cell type and culture conditions must be specified. | Exact concentration, exposure duration, solvent concentration, and cytotoxicity threshold should be reported. | Not applicable unless multiple concentrations are tested. If only one concentration is used, record as not determined. | Useful for preliminary screening when retinal cells, iPSC-derived retinal endothelial cells, RPE cells, Müller cells, or iBRB models are used. Limited relevance for non-retinal cell lines. | Vehicle control, untreated control, positive comparator, and cytotoxicity control. | Usually absent; therefore retinal exposure cannot be inferred. | Cell viability, membrane integrity, and assay-interference controls are needed. | Single readouts such as ROS fluorescence, VEGF ELISA, cytokine suppression, or viability assays may be vulnerable to PAINS or non-specific effects. | Interpreted as hypothesis-generating only, especially for polyphenolic or PAINS-prone metabolites. |
| Dose-response in vitro studies | Compound or nanoformulation must be chemically and physically defined. | Multiple concentrations and exposure durations should be reported; solvent and carrier concentrations must be controlled. | Can be extracted if the study identifies the lowest concentration that significantly changes a DR-relevant endpoint without cytotoxicity. | Moderate relevance when retinal cells or barrier models are used and endpoints reflect oxidative stress, inflammation, VEGF signaling, apoptosis, autophagy, or barrier integrity. | Vehicle, free compound, blank carrier, and positive comparator where applicable. | Not sufficient for retinal targeting unless followed by ocular PK or transport studies. | Cytotoxicity and barrier integrity should be assessed in parallel. | Dose-response improves pharmacological confidence but does not prove ocular delivery or clinical relevance. | Considered moderate-priority mechanistic evidence when paired with appropriate controls and orthogonal assays. |
| STZ-induced diabetic rodent studies | Active compound/formulation, dose, route, treatment duration, animal strain, sex, diabetes induction protocol, and hyperglycemia confirmation should be reported. | In vivo dose range, frequency, route, treatment start time, and treatment duration should be stated. | Lowest effective dose should be extracted when multiple doses are tested; otherwise record as NR. | Relevant to early NPDR-like hyperglycemia-associated vascular leakage, inflammation, BRB dysfunction, pericyte loss, and retinal neurodegeneration. STZ models mainly represent T1DM-like injury and usually do not progress to full human PDR. | Non-diabetic control, diabetic untreated control, vehicle/blank carrier, free compound, and positive comparator. | Retina/choroid/vitreous/plasma PK is needed when retinal targeting or enhanced delivery is claimed. | Body weight, glucose, liver/kidney markers, ocular histology, ERG/OCT/FFA where possible, and repeated-dose tolerance. | Without ocular exposure and functional endpoints, improved molecular markers alone do not establish translational efficacy. | Considered higher-priority preclinical evidence only when linked to retinal exposure, functional/vascular endpoints, and safety. |
| OIR rodent studies | Active compound/formulation, dose, route, oxygen exposure protocol, treatment timing, and animal strain should be reported. | Dose range and treatment window relative to vaso-obliteration and neovascularization phases should be specified. | Extract if multiple doses are tested; otherwise NR. | Relevant to ischemia-driven pathological neovascularization and proliferative-like retinal angiogenesis, but not a chronic hyperglycemic DR model. | Normoxia control, OIR untreated control, vehicle/blank carrier, free compound, and positive anti-angiogenic comparator. | Required for claims of retinal accumulation or targeted delivery to neovascular retina. | Retinal toxicity, vascular development effects, inflammation, and systemic toxicity should be considered. | OIR efficacy does not prove activity in chronic diabetic retinal disease. | Considered stage-specific preclinical evidence for angiogenesis/neovascularization, not general proof of DR efficacy. |
| Barrier penetration / ex vivo ocular transport studies | Carrier composition, size, surface chemistry, charge, ligand modification, and active compound loading must be defined. | Donor concentration, exposure time, tissue/model type, and assay conditions should be specified. | Lowest effective penetration-enhancing concentration can be reported if multiple doses are tested. | Relevant when using corneal models, RPE/iBRB Transwell models, vitreous diffusion systems, or ex vivo ocular tissues. | Free compound, blank carrier, non-modified carrier, and permeability marker controls. | Transport across cornea/RPE/vitreous should ideally be linked to tissue concentration measurements. | Barrier integrity, TEER, tight-junction markers, and cytotoxicity are required. | Increased permeability may reflect barrier damage rather than safe delivery. | Considered supportive delivery evidence, not proof of therapeutic efficacy. |
| PK / biodistribution studies | Compound/formulation identity, dose, route, sampling matrix, sampling time points, and analytical method must be reported. | Dose-normalized exposure should be provided where possible. | Not primarily applicable, but exposure threshold linked to efficacy should be explored. | Highly relevant when ocular matrices are quantified, including retina/choroid, vitreous, aqueous humor, cornea, and plasma. | Free compound and nanocarrier formulation comparison; blank matrix and validated analytical controls. | Essential endpoints include Cmax, Tmax, AUC, t1/2, tissue/plasma ratios, and retention time. LC-MS/MS or validated imaging mass spectrometry is preferred. | Toxic tissue accumulation and off-target systemic exposure should be assessed. | Without quantitative ocular PK, claims of retinal targeting or improved ocular bioavailability remain speculative. | Treated as essential translational evidence for delivery claims. |
| Safety and toxicity studies | Active compound, carrier, excipients, surfactants, dose, route, and repeated-dose schedule should be reported. | Maximum tested dose, no-observed-adverse-effect level if available, and exposure duration should be provided. | Lowest toxic concentration/dose and therapeutic window should be extracted when available. | Relevant when ocular irritation, retinal toxicity, inflammatory response, ERG, OCT, histology, and systemic toxicity are evaluated. | Untreated control, vehicle control, blank carrier, free compound, and positive irritant/toxicity control where appropriate. | Biodistribution helps determine whether toxicity relates to local ocular accumulation or systemic exposure. | Cytotoxicity, HET-CAM or equivalent irritation tests, 3D ocular models, retinal histology, inflammatory cytokines, ERG, IOP, liver/kidney markers, and repeated-dose tolerance. | Acute viability or irritation assays alone cannot establish long-term ocular safety. | Required before any claim of translational readiness or clinical potential. |

*Abbreviations: BRB, blood-retinal barrier; DR, diabetic retinopathy; ERG, electroretinography; FFA, fundus fluorescein angiography; iBRB, inner blood-retinal barrier; MPNS, Medicinal Plant Names Services; NLCs, nanostructured lipid carriers; NR, not reported; OIR, oxygen-induced retinopathy; PDI, polydispersity index; PK, pharmacokinetics; POWO, Plants of the World Online; RPE, retinal pigment epithelium; SLNs, solid lipid nanoparticles; STZ, streptozotocin.*

**Table S5. Representative pharmacological evidence and key limitations of studies discussed in this review**

| **Compound / formulation** | **Material definition** | **Model** | **Model relevance to DR** | **Main pharmacological or translational endpoint** | **PK / biodistribution evidence** | **Key limitations identified** | **Evidence interpretation** |
| --- | --- | --- | --- | --- | --- | --- | --- |
| Co-encapsulated silibinin + puerarin in nanostructured lipid carriers (NLCs) | Purified lipophilic silibinin and hydrophilic puerarin loaded into NLCs; carrier composition should be fully defined. | In vitro inflammatory model | Moderate: inflammation is DR-relevant, but in vitro evidence alone cannot establish retinal efficacy. | Reported 98.2% TNF-α inhibition in vitro. | “Optimized biodistribution” is mentioned, but detailed retinal/choroidal/vitreous/plasma exposure is NR. | Dose-response, lowest effective concentration, ocular exposure, and in vivo DR validation are not clearly provided. | Promising formulation-level and anti-inflammatory evidence, but should be interpreted as preclinical and hypothesis-generating unless supported by ocular PK and in vivo DR endpoints. |
| Baicalin-loaded liposomes | Baicalin-loaded liposomal formulation; active compound and liposome characteristics should be specified. | DR rodent model | High if model and endpoints were prespecified; DR-relevant vascular endpoints are reported. | Reported reductions in retinal VEGF expression and vascular leakage compared with free-drug controls. | Retinal efficacy reported; quantitative retina/plasma PK not clearly provided. | Dose range, lowest effective dose, time-course, randomization/masking, and full ocular PK are not specified in the current text. | Stronger than in vitro-only studies because it uses DR-relevant in vivo endpoints, but retinal targeting claims still require quantitative ocular PK. |
| Quercetin nanoemulsions | Quercetin-loaded nanoemulsion; droplet size, PDI, zeta potential, loading, and release profile should be defined. | Preclinical ocular/DR-relevant model | Moderate to high depending on original model details. | Extended retinal residence time 4.2-fold. | Retinal residence time reported. | Disease-modifying efficacy, dose-response, safety, and retina/plasma ratios are not fully described. | Useful delivery evidence, but extended residence time alone does not prove therapeutic efficacy. |
| Quercetin-loaded solid lipid nanoparticles (SLNs) | Quercetin encapsulated in SLNs; formulation attributes should include size/PDI/zeta potential/loading/release. | Retinal endothelial cells (RECs) under hypoxic / DR-relevant stress | Moderate: RECs and VEGF/HIF-1α are DR-relevant, but in vitro only. | Reduced VEGF expression by targeting HIF-1α stability | No ocular PK; cellular efficacy only. | In vitro system; potential PAINS/interference concerns for flavonoids; no retinal exposure or in vivo validation. | Mechanistically suggestive evidence, not sufficient to claim clinical or retinal therapeutic efficacy. |
| Curcumin- or resveratrol-loaded polymeric nanocarriers | Polymeric nanocarriers loaded with curcumin or resveratrol; active identity and carrier parameters should be defined. | RPE cells exposed to hyperglycemia-induced inflammatory conditions | Moderate: RPE inflammation is DR-relevant, but cell-only evidence is limited. | Suppressed IL-6 and TNF-α release compared with free compounds; linked to NF-κB pathway inhibition. | No ocular PK / biodistribution. | Single-pathway cytokine endpoints; dose-response and orthogonal validation unclear; curcumin/resveratrol may be PAINS-prone. | Hypothesis-generating anti-inflammatory evidence requiring dose-response, target engagement, and in vivo ocular exposure. |
| Gypenoside XVII (Gyp-17) in chitosan-alginate nanocarriers | Natural product loaded into chitosan-alginate nanocarriers; formulation and purity should be defined. | Müller cells under high-glucose stress | Moderate: Müller-cell dysfunction is relevant to DR neurovascular pathology. | Activated AMPK/mTOR pathways, increased autophagosome formation by 40%, and mitigated high-glucose-induced apoptosis. | No ocular PK / biodistribution. | In vitro cell model; autophagy flux interpretation requires rigorous controls; no retinal exposure or in vivo confirmation. | Mechanistic screening evidence, not confirmatory pharmacological evidence. |
| Norkurarinone | Purified plant-derived metabolite; formulation unclear or not specified in current text. | RPE viability / autophagy model | Moderate: RPE is DR-relevant, but model details and disease context are limited. | Inhibited excessive autophagy via PI3K/AKT and preserved RPE viability. | NR | No nanoformulation detail, no ocular PK, dose-response unclear. | Mechanistic evidence only; should not be used to support delivery or retinal targeting claims. |
| Epigallocatechin-conjugated gold nanoparticles (AuNP-EGCG) | Epigallocatechin conjugated to gold nanoparticles; nanoparticle size, surface chemistry, loading, and stability should be defined. | Retinal endothelial cells and RGC co-culture / translational ocular model | Moderate: retinal cells are relevant, but disease-model specificity is not fully defined in the manuscript. | Demonstrated 80% VEGF reduction in RECs, sustained RGC protection in co-culture, and >12-hour retinal retention. | Retinal retention >12 h reported. | Gold carrier safety, long-term retention, dose-response, and DR-specific in vivo validation require clarification. | Promising but requires careful safety and DR-specific validation before strong translational claims. |
| Puerarin: oral vs. localized administration | Purified puerarin; botanical source and purity should be defined. | Rat oral bioavailability study; rabbit retinal tissue PK after localized administration | PK-relevant; not necessarily disease-model efficacy. | Oral bioavailability reported as 7% in rats; localized route achieved retinal Cmax of 0.21 ± 0.05 μg/mg in rabbits. | Retinal Cmax reported for localized administration. | No direct retinal-to-plasma comparison across clinically relevant topical/intravitreal routes; efficacy endpoints not included. | Useful PK evidence showing delivery barrier, but not proof of DR efficacy. |
| Tetramethylpyrazine | Purified compound; source/purity should be defined. | Rat intraocular and intraperitoneal administration studies | PK / barrier-relevance; limited direct DR efficacy relevance. | Intraocular administration reportedly yielded greater systemic bioavailability than oral route; intraperitoneal injection allowed BRB penetration and retinal accumulation. | Retinal accumulation mentioned. | Quantitative retina/plasma ratios and clinically relevant topical/intravitreal comparisons are lacking. | Supports need for route-specific PK evaluation, but not sufficient for therapeutic claims. |
| Notoginsenosides / Panax notoginseng-derived compounds | Multi-component saponin fraction or botanical-derived compounds; marker compounds and source should be defined. | Oral absorption / PK studies | Low to moderate: systemic PK relevance, but limited retinal specificity. | Poor oral absorption reported. | No retinal targeting or ocular PK information. | Composition may be multi-component; retinal exposure, dose-response, and DR efficacy not established. | Evidence mainly identifies a knowledge gap; should not be used to claim retinal therapeutic potential. |
| Lutein-loaded nanoemulsions (Lutein-NEL) | Lutein-loaded nanoemulsion; formulation parameters should be defined. | Preclinical DR model | Moderate to high depending on original model details. | Enhanced retinal antioxidant capacity and reduced GFAP overexpression. | Ocular exposure not clearly described. | Dose-response, lowest effective dose, retinal/plasma PK, and long-term safety unclear. | Supportive preclinical antioxidant/neuroglial evidence, but translational strength is limited by missing PK and dose details. |
| Baicalin nanodrops | Baicalin-containing nanodrop formulation; formulation parameters should be defined. | Preclinical DR model | Moderate to high depending on original model details. | Enhanced retinal antioxidant capacity and reduced GFAP overexpression. | Ocular exposure not clearly described. | Dose-response, retinal/choroidal/vitreous/plasma concentration data, and safety endpoints are not fully described. | Supports potential ocular delivery benefit, but requires quantitative ocular PK and controlled in vivo validation. |
| Berberine-loaded chitosan-based nanocarriers | Berberine-loaded chitosan nanocarrier; particle size/PDI/zeta potential/loading/release should be defined. | Rabbit ocular / retinal bioavailability model | PK-relevant; not necessarily DR disease efficacy. | Improved sustained release and showed 2.3-fold higher retinal bioavailability than oral administration. | Retinal bioavailability measured by HPLC analysis of vitreous samples. | DR efficacy endpoints, dose-response, comparator design, and long-term ocular tolerance are not fully described. | Stronger delivery / PK evidence than cell-only studies, but disease-modifying efficacy remains unproven. |

*Abbreviations: BRB, blood-retinal barrier; DR, diabetic retinopathy; ERG, electroretinography; NLCs, nanostructured lipid carriers; NR, not reported in the source description; PAINS, pan-assay interference compounds; PDI, polydispersity index; PK, pharmacokinetics; RECs, retinal endothelial cells; RGCs, retinal ganglion cells; RPE, retinal pigment epithelium; SLNs, solid lipid nanoparticles; STZ, streptozotocin.*
